# An “expressionistic” look at serrated precancerous colorectal lesions

**DOI:** 10.1186/s13000-020-01064-1

**Published:** 2021-01-10

**Authors:** Giancarlo Marra

**Affiliations:** Institute of Molecular Cancer Research, University of Zurich, Winterthurerstrasse 190, 8057 Zurich, Switzerland

**Keywords:** Sessile serrated lesion, Hyperplastic polyp, Traditional serrated adenoma, Adenomatous polyp, Colorectal cancer, Gene expression, In situ hybridization, Tissue staining markers

## Abstract

**Background:**

Approximately 60% of colorectal cancer (CRC) precursor lesions are the genuinely-dysplastic conventional adenomas (cADNs). The others include hyperplastic polyps (HPs), sessile serrated lesions (SSL), and traditional serrated adenomas (TSAs), subtypes of a class of lesions collectively referred to as “serrated.” Endoscopic and histologic differentiation between cADNs and serrated lesions, and between serrated lesion subtypes can be difficult.

**Methods:**

We used in situ hybridization to verify the expression patterns in CRC precursors of 21 RNA molecules that appear to be promising differentiation markers on the basis of previous RNA sequencing studies.

**Results:**

SSLs could be clearly differentiated from cADNs by the expression patterns of 9 of the 12 RNAs tested for this purpose (*VSIG1*, *ANXA10*, *ACHE*, *SEMG1*, *AQP5*, *LINC00520*, *ZIC5/2*, *FOXD1*, *NKD1*). Expression patterns of all 9 in HPs were similar to those in SSLs. Nine putatively HP-specific RNAs were also investigated, but none could be confirmed as such: most (e.g., *HOXD13* and *HOXB13*), proved instead to be markers of the normal mucosa in the distal colon and rectum, where most HPs arise. TSAs displayed mixed staining patterns reflecting the presence of serrated and dysplastic glands in the same lesion.

**Conclusions:**

Using a robust in situ hybridization protocol, we identified promising tissue-staining markers that, if validated in larger series of lesions, could facilitate more precise histologic classification of CRC precursors and, consequently, more tailored clinical follow-up of their carriers. Our findings should also fuel functional studies on the pathogenic significance of specific gene expression alterations in the initiation and evolution of CRC precursor subtypes.

**Supplementary Information:**

The online version contains supplementary material available at 10.1186/s13000-020-01064-1.

## Background

The World Health Organization’s GLOBOCAN database currently shows colorectal cancer (CRC) as the third most commonly diagnosed cancer in males and the second in females, with 1.8 million new cases and almost 861,000 deaths in 2018. But it is also singularly preventable. Its onset is preceded by an interval of approximately 10–15 years, during which benign lesions with different malignant potentials are present in the colon and can be effectively eliminated during screening colonoscopy [1–4]. Decades of screening colonoscopy data have provided us with a fairly reliable estimate of the cancer risk posed by conventional colorectal adenomas (cADNs), genuinely-dysplastic lesions that account for around 60% of precancerous colon tumors [5, 6]. Less is known about the malignancy risk of the more recently defined “serrated” precancerous lesions, so called because of the saw-tooth-like epithelial infolding found in their crypt lumens [7, 8]. The past 20 years have witnessed active efforts to characterize these lesions, endoscopically, histologically, and molecularly. Three types of serrated lesions are currently recognized: hyperplastic polyps (HPs), traditional serrated adenomas (TSAs), and sessile serrated lesions (SSL) (the term currently recommended by the World Health Organization for lesions previously referred to as sessile serrated adenoma/polyps) [6, 9–11]. HPs and TSAs arise mainly in the distal colon and rectum. HPs account for ~ 30% of all benign colorectal lesions, and their risk of transformation is considered to be very low. They are generally believed, however, to be precursors to at least some SSLs and TSAs. (The putative pathways underlying these progressions are discussed in a recent review [10].) The cancer risk of TSAs is probably similar to that of cADNs, but they represent only ~ 1% of precancerous colorectal lesions [10]. As for SSLs, which are usually found proximal to the splenic flexure, they represent about 10% of all precancerous colorectal tumors detected with high-performance colonoscopy [4], and yet they appear to give rise to almost 20% of all CRCs. The long-term risk of developing CRC after endoscopic removal of a large or “advanced” SSL (i.e., one measuring ≥10 mm) is as high as that associated with removal of a similarly sized cADN [12, 13].

Most CRCs thus arise from cADNs or SSLs. The latter are easier to miss during colonoscopy, partly because of their propensity for the proximal colon. Endoscopic visibility in this area is often reduced due to an insufficient bowel prep, and the examination is frequently marred by technical shortcomings (e.g., omission of cecal intubation, excessively rapid scope withdrawal times). Morphology also plays a role [7, 14]. SSLs are nearly always flat or sessile lesions with indistinct borders and colors resembling that of the normal mucosa. They can also be obscured by a mucus cap. Studies conducted using same-day tandem examinations found that ~ 25% of all precancerous colon lesions are missed on colonoscopy [15]. The miss rate dropped to ~ 10% for all lesions measuring ≥10 mm, but it remained high (~ 25%) for the lesions that were sessile or flat, the subset that includes SSLs. Most CRCs detected within 3 years of a negative colonoscopy derive from missed or incompletely-excised lesions, and these “interval” or “post-colonoscopy” malignancies are characterized by an over-representation of proximal-colon locations and cancers that develop along the serrated tumorigenic pathway [16–19].

Three-quarters of the CRCs arising in SSLs (10–15% of all CRCs) have a well-defined phenotype characterized by location in the proximal colon, the BRAFV600E gain-of-function mutation, and methylation of CpG islands cytosines that are generally unmethylated in the DNA of the normal colorectal mucosa [10, 20, 21]. Most of these CIMP (CpG island methylator phenotype) cancers are also DNA mismatch repair (MMR)-deficient owing to methylation of the promoter CpG island of the MMR gene *MLH1* (a well-known CIMP target) [20, 22]. As a result, *MLH1* expression is silenced, and MMR becomes deficient, as reflected by increased mutation rates and DNA microsatellite instability.

Precancerous lesions can be differentiated endoscopically to some extent, but the process is by no means simple. The location of the lesion within the colon is poorly informative. Conventional adenomas can arise anywhere in the colon. The serrated lesions display some degree of segmental preference (SSLs for the proximal colon, HPs and TSAs for the distal colon and rectum), but these preferences are by no means absolute. Lesion morphology is also of limited value. The Paris classification [23] distinguishes polypoid (i.e., stalked or sessile) precancerous colorectal lesions from those that are nonpolypoid (less elevated than a sessile lesion or flat or depressed relative to the surrounding non-lesional tissue). However, sessile lesions can be cADNs, SSLs, or HPs, whereas TSAs usually resemble cADNs in terms of their elevation above the mucosal plane. The “pit pattern” of a precancerous lesion, as defined during high-magnification endoscopic examination of the crypt openings on its surface, can reliably predict the histologic diagnosis [24]. However, this approach is not used routinely in most gastroenterology centers, and the results still require histologic confirmation.

Histologic diagnosis itself is also far from being straightforward. Disagreement arises among pathologists regarding several types of resected precancerous colorectal lesions, and the issues underling this inter-examiner variability have been well-reviewed elsewhere [6, 10]. Suffice it to say here that the two most frequently encountered high-risk lesions, cADNs and SSLs, can be readily differentiated, since SSLs rarely exhibit dysplasia. Doubts can arise, however, with “advanced” lesions: SSLs (and TSAs) measuring ≥10 mm can harbor cytologic and architectural features of dysplasia. In this case, differential diagnosis with cADNs might be an issue, especially when the dysplasia resembles that typically found in cADNs [6, 10, 25, 26]. Some lesions also display intratumoral heterogeneity, with certain areas resembling cADN and others more typical of SSL or TSA. TSAs are rare lesions with fairly typical histologic features consisting of eosinophilic cells with elongated nuclei, ectopic crypt foci, and slit-like serration. However, they can be misdiagnosed as cADNs, especially when they are large, polypoid and dysplastic, and located in the distal colon. Large, relatively flat TSAs in the proximal colon can also be mistaken for SSLs.

Problems can also arise in differentiating SSLs and HPs [6, 10, 27–29]. Both display crypt lumens with the characteristic saw-toothed pattern. But in HPs the serration does not extend to the base of the crypts, whereas in SSLs it involves the entire longitudinal axis of the gland, disrupting the simple tubular architecture with asymmetric dilatations that results in bizarre boot- or anchor-shaped crypts. Differential diagnosis is facilitated if the histologic section has been cut parallel to the longitudinal axis of the serrated crypts, but attention is rarely devoted to proper orientation of the specimens resected during endoscopy. Given the markedly different cancer risks associated with SSLs (high) and HPs (very low), an additional tissue staining procedure that would aid pathologists in reliably differentiating between these two types of serrated lesions seems desirable.

Driver gene mutations can also be somewhat informative for typing precancerous colorectal lesions. *APC* mutations, for example, are typical of cADNs, but these lesions (and in rare cases SSLs) can also harbor *KRAS* mutations, which are characteristic of TSAs. And while the BRAFV600E mutation is considered typical of SSLs, it can also be found in HPs [10]. As for TSAs, they are generally thought to progress along the *KRAS*-mutated molecular pathway, but progression also appears to occur along the BRAFV600E or *KRAS/BRAF*-wild type pathway [30]. Recent molecular studies have revealed that signaling pathways typically involved in colorectal tumorigenesis (e.g., Wnt signaling) have different pathogenic trajectories during the evolution of different CRC precursor types [31–33]. For example, somatic *APC* mutations lead to early constitutive activation of canonical Wnt signaling in cADNs, whereas aberrant Wnt signaling occurs later in SSL and TSA tumorigenesis and is triggered by epigenetic silencing (via CIMP) or genetic mutations affecting Wnt-signaling modulators or antagonists (e.g., *SFRPs, AXIN2*, *RNF43,* or *RSPOs*) [10, 34–36]. These differences are also reflected in the transcriptional outputs of the aberrant Wnt signaling, and characterization of the various gene expression profiles would therefore allow more precise classification of precancerous colorectal lesions.

Attempts have recently been made to differentiate CRC precursor lesions based on high-throughput transcriptome profiling data. RNA sequencing studies of serrated lesions and cADNs by our group [21] and that of Delker [37, 38] have identified a large number of putative gene expression markers that could be used for this purpose, following their verification and validation with in situ hybridization (ISH) and immunohistochemistry. In the study described below, we used ISH to verify the expression profiles of 21 transcriptome-based RNA molecules that appeared to be promising markers for differentiation between CRC precursors. Such markers could be exploited to create simple tissue-staining tools for refining their routine histologic diagnosis. They could also help pinpoint the molecular pathways active in a given lesion, as a proxy for more complicated genetic and epigenetic analyses. Paradigmatic is the immunostaining for MLH1: negative results indicate that an SSL is dysplastic, almost invariably *BRAF*-mutated, CIMP-positive, and microsatellite instable. And close endoscopic follow-up is required after excision of this type of lesion [10].

## Methods

### Tissues

Formalin-fixed, paraffin-embedded colorectal tissues were obtained from the Zurich University Hospital Pathology Archives with local ethics committee approval (No. 2015–00185). Donors provided written consent to tissue testing and data publication. Samples were coded to protect donors’ rights to confidentiality and privacy. None of the donors had a family history of colorectal cancer. SSLs displayed no evidence of dysplasia and exhibited normal staining for the MLH1 protein. The routine histologic diagnosis was confirmed with a second evaluation by a gastrointestinal pathologist. Low magnification, H&E (hematoxylin and eosin) images of each colorectal lesion are shown in supplementary material.

### In-situ hybridization (ISH)

**Fig. 1:**
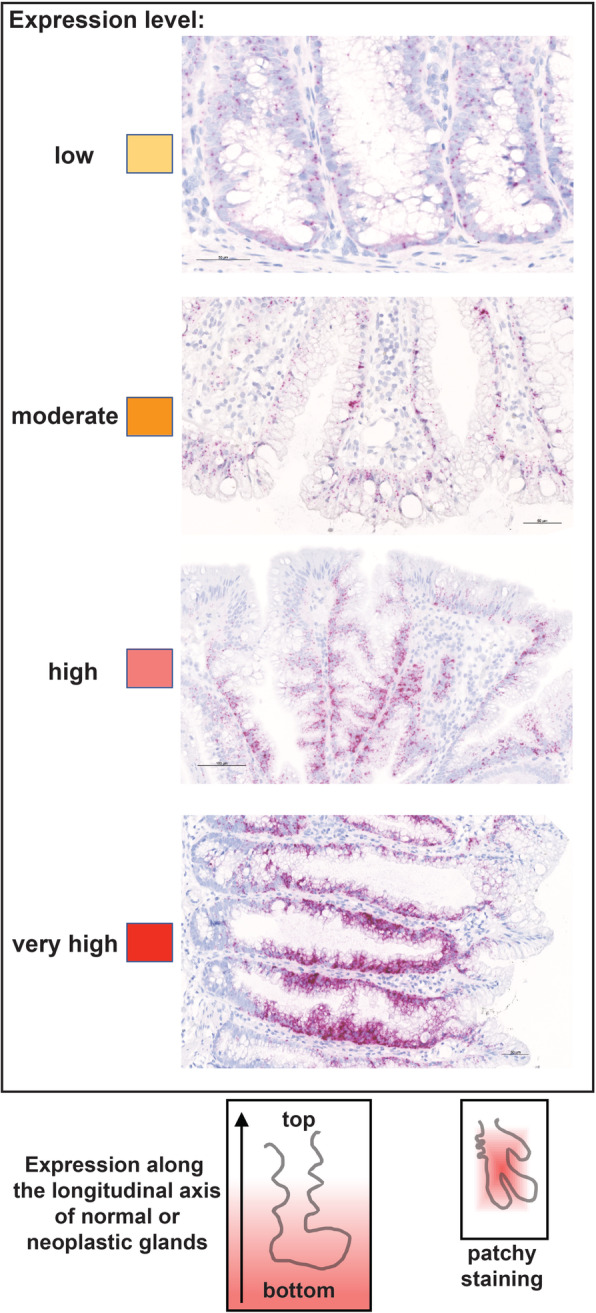
RNA-expression scoring of colorectal tissue sections. Intensity and distribution of epithelial cell expression of target RNAs were assessed in the entire section. IHC staining intensity was classified as *low*, *moderate*, *high,* or *very high* based on the number and size of dots and dot aggregates in the epithelial cells. Each score is color-coded according to the yellow-to-red scale used in Table 1. ISH staining distribution patterns were classified as shown in the schematics at the bottom of this figure: (*left*) staining distributed along the entire length of the longitudinal axis of normal and neoplastic glands, from the bottom to the luminal portion, in regions of the section where the specimen was properly oriented; (*right*) “patchy staining” consisting of stained and unstained regions within the same section, which is depicted as a rectangular gradient fill whose area is proportional to the prevalence and size of the stained patches across the section. Both staining distribution features are also depicted in Table 1 for each RNA. Distribution of staining along the longitudinal crypt axis was generally more common in normal mucosa, HPs, and SSLs (well-oriented specimens of flat or slightly elevated lesions); patchy staining patterns were found mainly in polypoid cADNs and TSAs, whose thick epithelium often contains intricately-folded, dysplastic glands

Three-micrometer-thick tissue sections were mounted on SuperFrost Plus slides (Thermo Scientific, Reinach, Switzerland), stored at 4 °C, and analyzed with ISH analysis within 1 month after sectioning. Deparaffinized sections were processed manually using the *RNAscope 2.5 HD Red* reagents from Advanced Cell Diagnostics (presently Bio-Techne, Abingdon, United Kingdom), according to a protocol based on branched-DNA technology [39, 40]. In brief, after blockade of endogenous peroxidase activity, epitope retrieval, and protease digestion steps, the sections were subjected to ISH with 20 pairs of primary oligonucleotide probes for each mRNA of interest. Each probe pair targeted two consecutive 20-to-30-nt regions at a given position within the transcript. (Hybridization of only three of the 20 pairs is sufficient to obtain a signal that can be detected with standard microscopy.) Each hybridized probe pair was then bound by a series of complementary amplification molecules and labeled probes containing a chromogenic enzyme, which markedly enhance signal detection sensitivity. High specificity is also ensured since the amplification cascade begins only after both members of the primary oligonucleotide probe pair have hybridized to the target transcript. The chromogenic reaction generates a single punctate signal per RNA molecule. Dot size is generally proportional to the number of primary oligonucleotide probe pairs hybridized to the target RNA molecule, and dot aggregates indicate high concentrations of the target. Finally, nuclei were weakly stained with hematoxylin and the slides scanned with an Axio Scan.Z1 (Zeiss, Feldbach, Switzerland). Images shown in this report were obtained from these scannings using the ZEN 2 microscope software (Zeiss, Feldbach, Switzerland). As illustrated in Fig. 1 and Table 1, the staining intensity was arbitrarily classified as low, moderate, high, or very high, while the staining distribution was reported both along the longitudinal axis of glands and across the whole section.

**Table 1:**
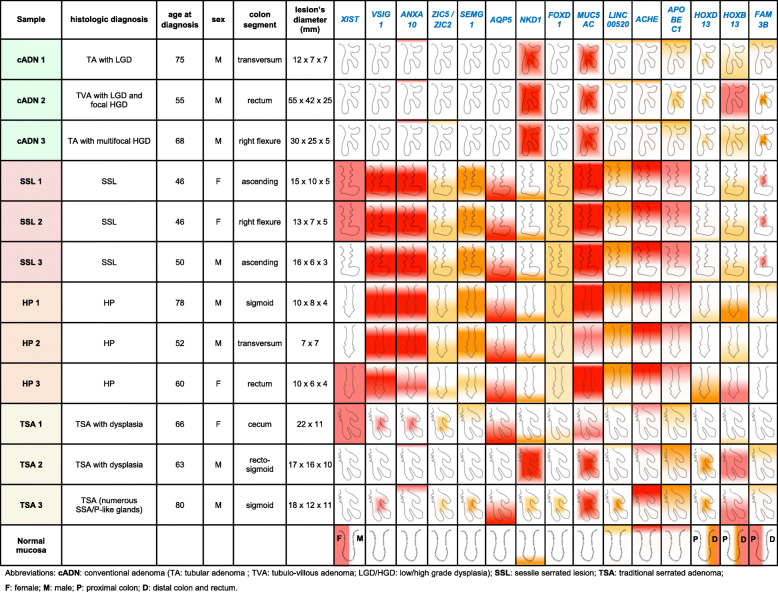
Clinical and in situ hybridization data of the analyzed tumors

## Results

We analyzed 12 premalignant colorectal tumor samples (3 cADNs; 3 SSLs; 3 HPs; and 3 TSAs) from the pathology archives of Zurich University Hospital (Table 1**)**. All but one (HP 2) measured ≥10 mm and were therefore considered “advanced” lesions.

Sections cut from each tumor block were processed for ISH. Each section was hybridized with primary oligonucleotide probes for one of the 21 RNA targets investigated (**Supplementary Table** **1**) or one of the three RNAs used as staining controls (**Supplementary Figure** **1**). In each section, staining of the normal mucosal crypts at the border of each lesion was assessed as an internal normal-tissue control. The choice of the RNA targets to be verified was based on RNA-sequencing data on precancerous colorectal lesions previously published by our group [21] (**Supplementary Figure** **2****)** and others [38] (**Supplementary Figure** **3****)**.

**Fig. 2:**
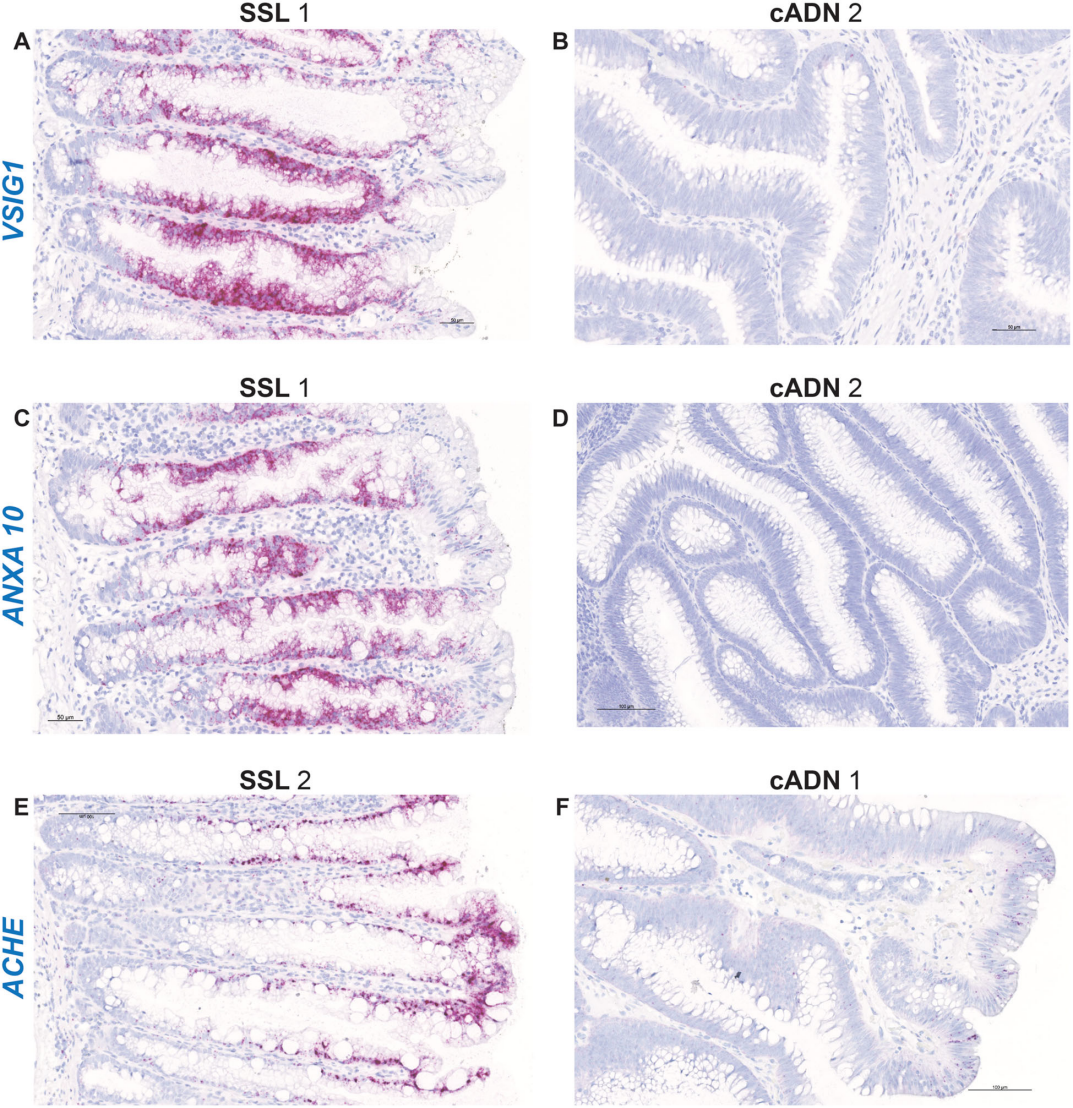
In situ hybridization analysis of *VSIG1*, *ANXA10,* and *ACHE* mRNAs in SSLs and cADNs. All three markers are strongly positive in SSLs (dense red punctate labeling in panels **A**, **C**, and **E**, reflecting “very high” expression (see scoring system depicted in Fig. 1) but absent in cADNs (panels **B**, **D**, and **F**) with the exception of few *ANXA10*- or *ACHE*-positive cells on their surface. While *VSIG1* and *ANXA10* are expressed in most of the longitudinal axis of the serrated glands, except their bottoms and tops, *ACHE* expression involves mainly the upper half of these crypts. Details on the expression patterns of these three mRNAs in the investigated lesions and in normal mucosa are shown in Supplementary Figs. 4, 5 and 6. Lesions are numbered as in Table 1

ISH verification of RNA target expression patterns was undertaken to identify bona fide markers for distinguishing SSLs from cADNs. To this end, we focused our analysis on 12 mRNA targets (Supplementary Table 1). As shown in Table 1, SSLs and cADNs could be readily differentiated from one another using 9 of the 12 candidate markers (8 whose expression was SSL-specific [*VSIG1*, *ANXA10*, *ACHE*, *SEMG1*, *AQP5*, *LINC00520*, *ZIC5*, *FOXD1*] and 1 with cADN-specific expression [*NKD1*]). The ISH expression patterns of these mRNAs in SSLs and cADNs are shown in **Figs.** **2****,** **3**, **4** and **5**, and **Supplementary Figures** **4**, **5**, **6**, **7**, **8**, **9**, **10**, **11** and **14****.** Two of the three remaining candidates (*APOBEC1* and *MUC5AC*) were expressed more intensively in SSLs than in cADNs, but their expression was by no means SSL-specific (**Figs.** **4** and **5****,** and **Supplementary Figures** **12** and **13**). As for the third, the putatively SSL-specific marker *KLK8* (Supplementary Table 1), its staining pattern was uninformative (results not shown), probably due to cross-hybridization with other KLK-family members.

**Fig. 3:**
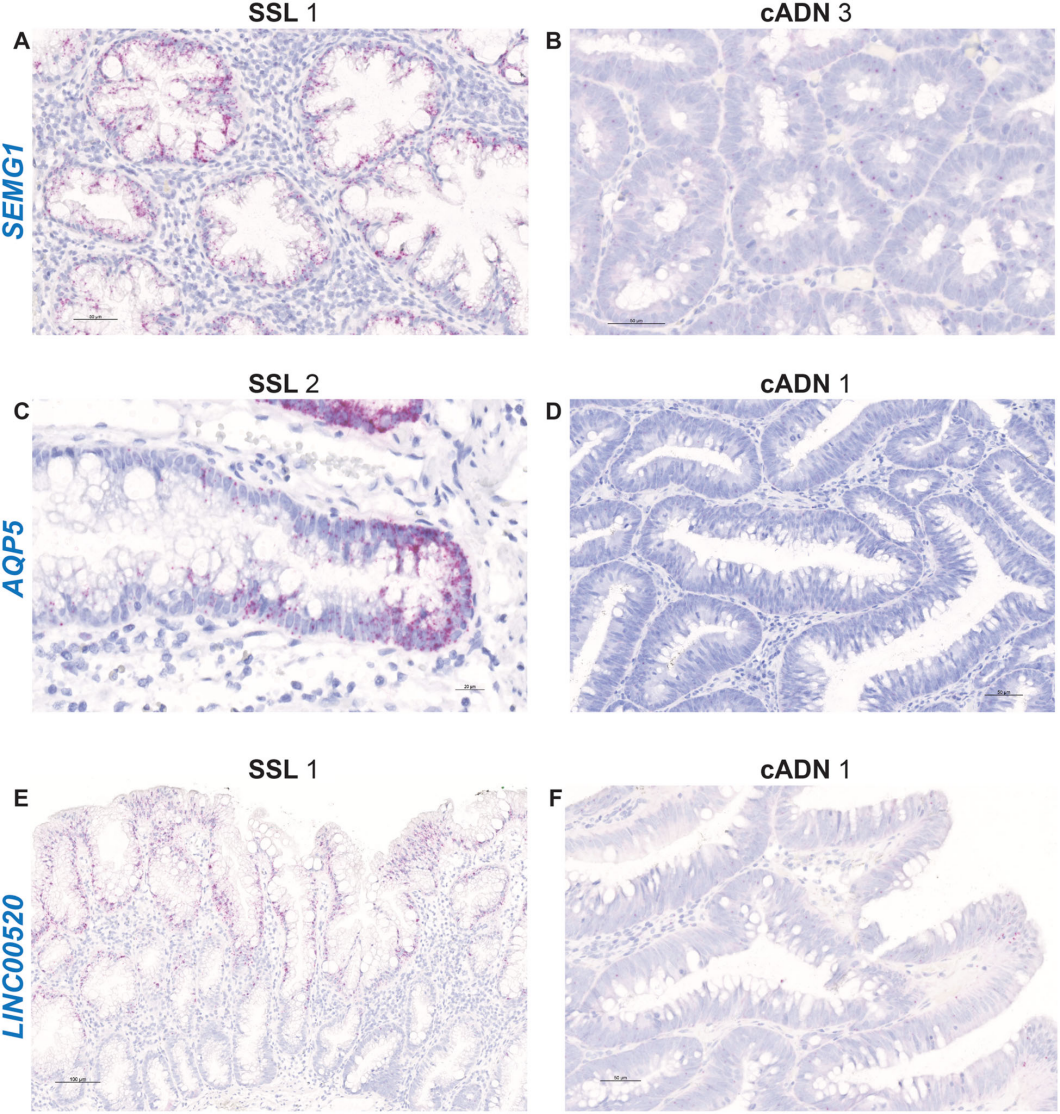
In situ hybridization analysis of *SEMG1*, *AQP5,* and *LINC00520* RNAs in SSLs and cADNs. These three RNAs are “moderately” to “very-highly” expressed in SSLs, but absent in cADNs with the exception of few *LINC00520*-positive superficial cells. Details on the expression patterns of these three RNAs in the investigated lesions and in normal mucosa are shown in Supplementary Figs. 7, 8, and 9, and summarized in Table 1

**Fig. 4:**
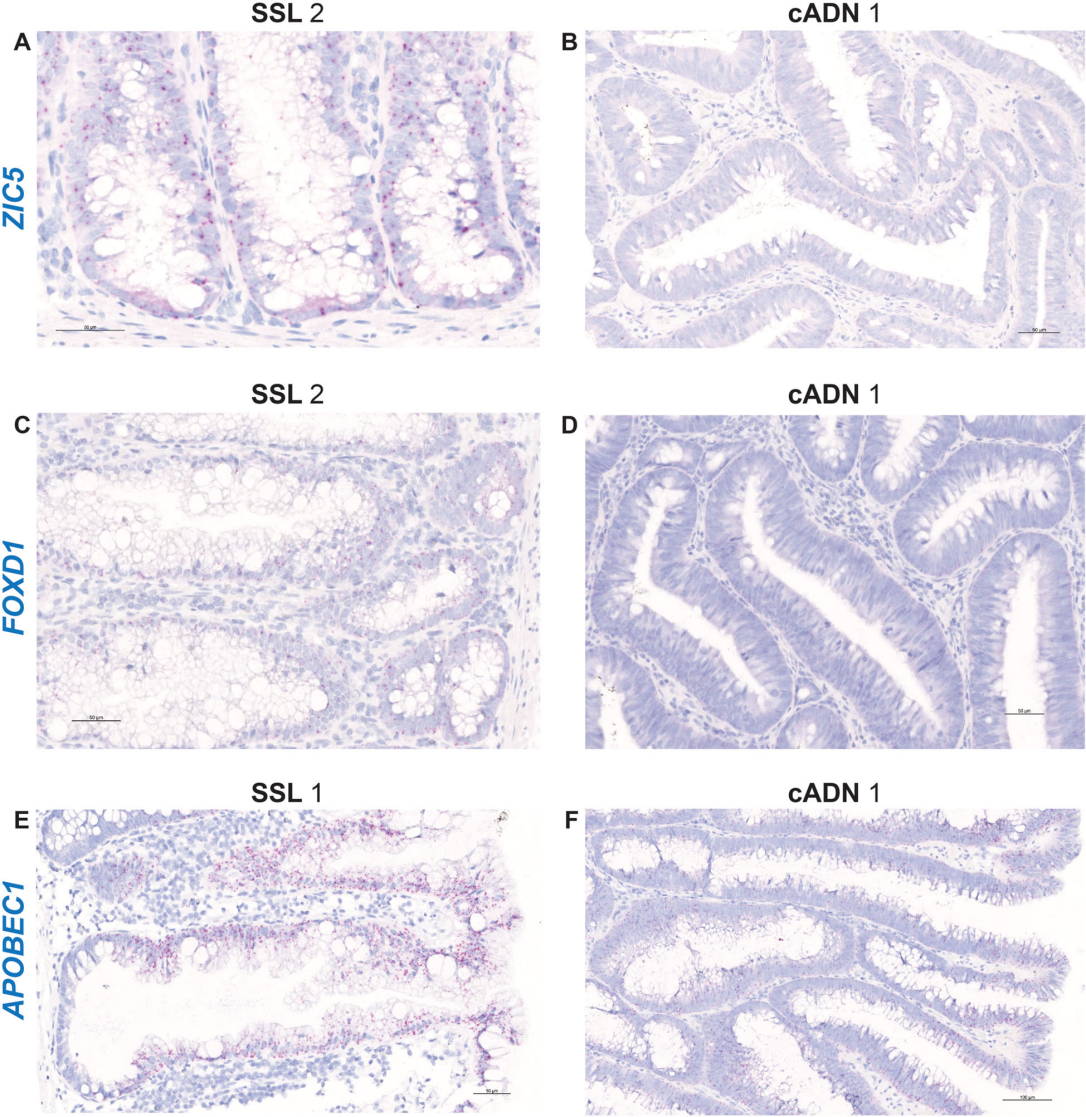
In situ hybridization analysis of *ZIC5*, *FOXD1,* and *APOBEC1* mRNAs in SSLs and cADNs. While *ZIC5* and *FOXD1* are expressed in SSLs but not in cADNs, *APOBEC1* is moderately expressed in cADNs (superficially or irregularly) albeit less abundantly than in SSLs. Details on the expression patterns of these three RNAs in the investigated lesions and in normal mucosa are shown in Supplementary Figs. 10, 11, and 12, and summarized in Table 1

**Fig. 5:**
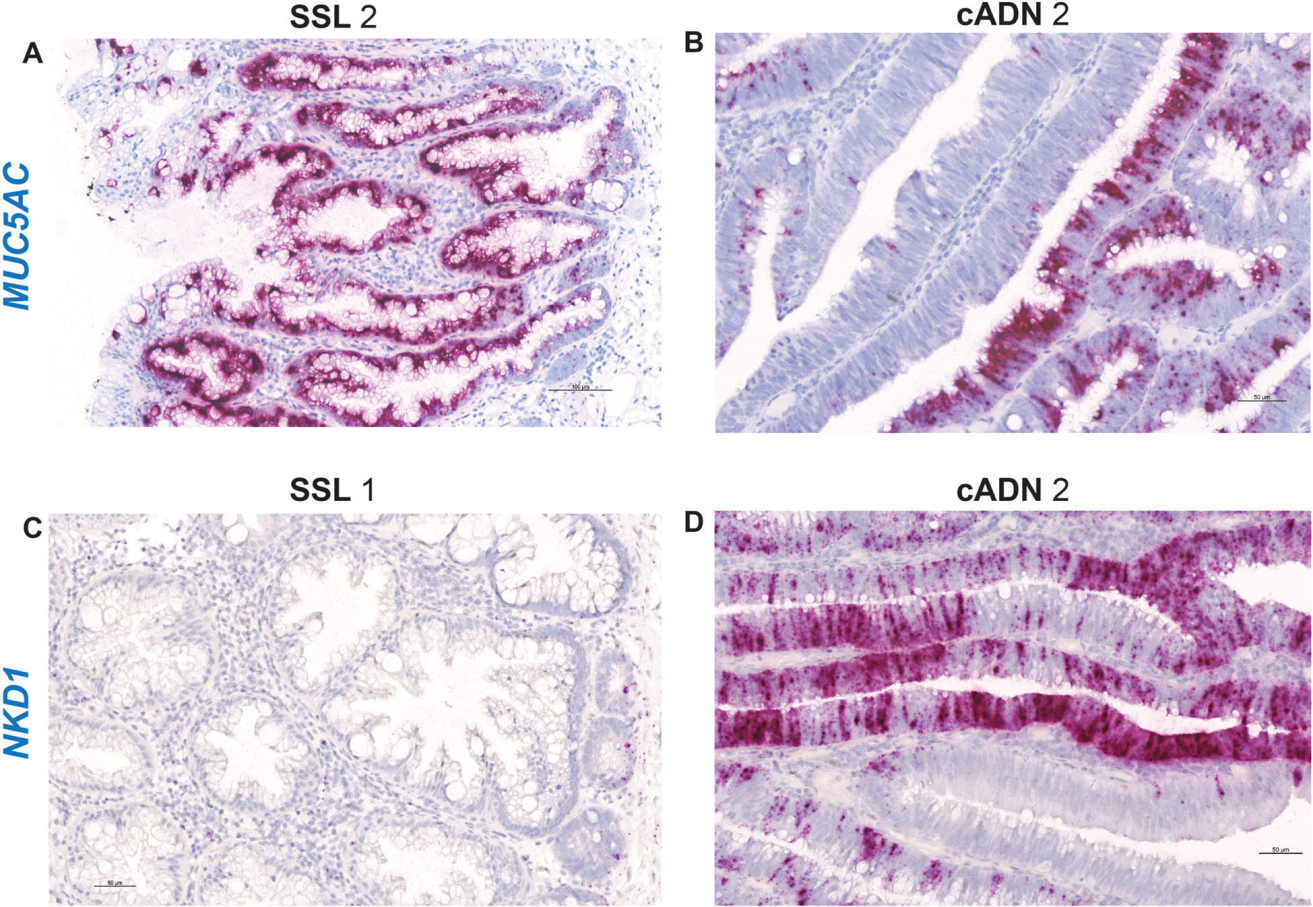
In situ hybridization analysis of *MUC5AC* and *NKD1* mRNAs in SSLs and cADNs. *MUC5AC* is not a specific marker of SSLs as suggested by RNA-sequencing data: it is very highly expressed also in cADNs, although less extensively than in SSLs (i.e, patchy staining). *NKD1* is very highly expressed in cADNs, but not in SSLs with the exception of a few cells at the bottom of their serrated glands. Details on the expression patterns of *MUC5AC* and *NKD1* mRNAs in the investigated lesions and in normal mucosa are shown in Supplementary Figs. 13 and 14, respectively, and summarized in Table 1

**Fig. 6:**
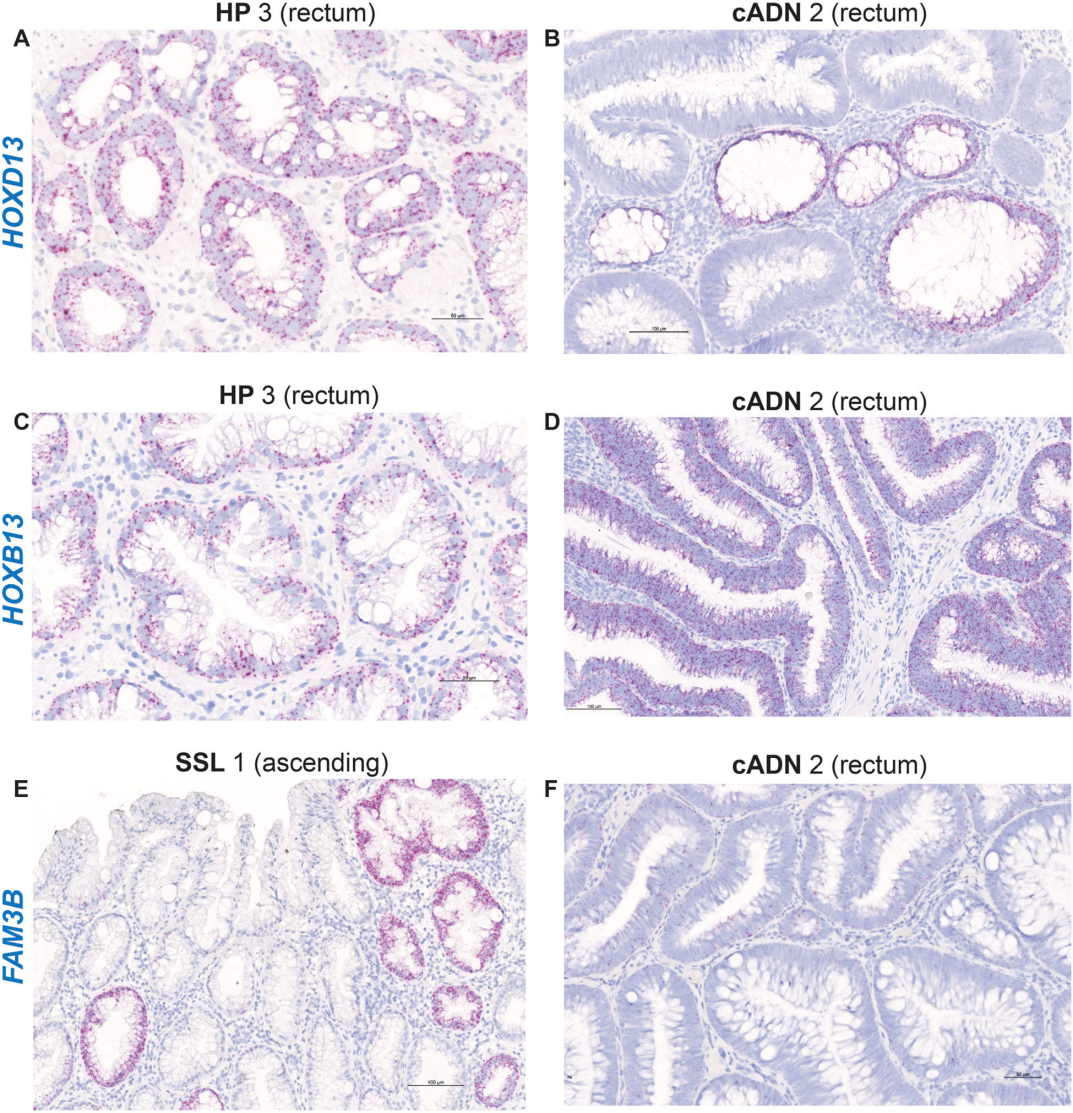
In situ hybridization analysis of *HOXD13*, *HOXB13*, and *FAM3A* mRNAs in serrated lesions and cADNs. These three mRNAs are not specifically expressed in serrated lesions versus cADNs or vice versa. While *HOXD13* and *HOXB13* are expressed prevalently in lesions of the distal colon and rectum, *FAM3A* is prevalently found in proximal colon lesions: these patterns reflect the expression specificity in the normal mucosa in the distal and proximal segments of the large intestine. Details on the expression patterns of these three mRNAs in normal mucosa and in the investigated lesions are shown in Supplementary Figs. 15, 16, and 17, and summarized in Table 1

The ISH data summarized in Table 1 also highlight the similarity between the expression patterns of the SSL-specific markers in SSLs and HPs (Supplementary Figures 4, 5, 6, 7, 8, 9, 10 and 11 and 14). These two serrated lesion types could not be distinguished even with the 9 RNAs that were chosen (on the basis of RNA sequencing data) to differentiate HPs from all other precancerous lesions [38] (e.g., *HOXD13*, *HOXB13,* and *FAM3A* [Supplementary Table 1, Table 1, Fig. 6 and **Supplementary Figures** **15****,** **16** and **17**]). *HOXD13* and HOXB13, in fact, seem to be genuine markers not of HPs but of the normal mucosa in the distal colon and rectum, where HPs usually arise. These findings suggest that the specific expression of *HOXD13* and *HOXB13* in the distal half of the colon (vs. the proximal colon) is retained in lesions typically occurring in this colorectal segment (Table 1). The upregulated expression of these two genes in HP biopsies processed for RNA sequencing might also have stemmed from normal mucosal contamination of the tumor sample (not uncommon with biopsy of lesions as small as most HPs). These interpretations are also consistent with the expression patterns of *EVX2* (a *HOXD13* neighbor), *PRAC1* (a *HOXB13* neighbor), *INSL5*, *OR51E2*, *CPB1*, and *ST6GAL2* (Supplementary Table 1, Supplementary Figure 3, ISH data not shown). Along similar—albeit directionally opposite—lines, *FAM3B*, which was selected for verification because it was specifically unexpressed in HPs, proved instead to be a marker of the normal mucosa of the proximal-colon (HPs are generally located in the distal colon and rectum) (Fig. 6, Supplementary Figure 17). Therefore, none of the nine RNAs that were putatively HP-specific could be verified as such.

As shown in Table 1 and all figures, the ISH expression patterns of TSAs were heterogeneous and frequently characterized by a mixture of SSL- and cADN-specific staining patterns.

H&E-stained images of all 12 lesions investigated in this study are shown in **Supplementary Figures** **18**–**29**.

## Discussion

RNA-based gene expression profiles generated by our group have revealed numerous RNA markers that are differentially expressed in SSLs and cADNs [21]. Here, using ISH, we verified the accuracy of 9 of the 12 markers putatively capable of distinguishing between these two major premalignant tumor types. Those that appeared to be SSL-specific, however, were unable to differentiate these lesions from HPs. Evidently, the gene-expression trajectories underlying the early stages of serrated tumorigenesis in these two serrated precursor lesions are common (see Introduction). HP-specific markers were also not found among additional 9 RNAs investigated in this study. The advanced TSAs (> 10 mm diameter) we investigated showed mixed staining patterns reflecting the coexistence in each lesion of serrated and cADN-like histologic features (Table 1).

One of the three genes that displayed particularly high expression in SSLs and HPs was *VSIG1* (V-set and immunoglobulin domain containing 1) (Fig. 2, Supplementary Figure 4). Absent in cADNs and the normal colon mucosa, *VSIG1* expression was very high along almost the entire length of the serrated crypts in SSLs and HPs, except the very bottom of the crypt and its surface. TSAs, in contrast, display very little *VSIG1* expression or none at all (e.g., TSA 2 in Table 1). The expression that is observed is reflected by patchy staining confined to glands with an SSL-like phenotype. The strikingly different *VSIG1* expression patterns in serrated crypts (highly expressed) and those of the normal colorectal mucosa (unexpressed) might 1 day be exploited to improve detection of flat serrated lesions using fluorescein-labeled anti-VSIG1 antibodies during colonoscopy [41].

The VSIG1 protein, a member of the junctional adhesion molecule family, is normally expressed in the gastric mucosa and testis [42, 43]. Its ectopic expression in serrated colorectal lesions, which has been documented at both the transcript and protein levels [21, 37, 44], is thought to reflect aberrant differentiation toward a gastric-cell phenotype during the development of these tumors. SSLs and HPs also acquire expression of other molecules typically found in the gastric mucosa, e.g., ANXA10 (Annexin 10), a known marker of the normal mucosa of the stomach [45–47]. ANXA10 belongs to the calcium-dependent phospholipid-binding annexin protein family, and its function is currently unknown. Like VSIG1, ANXA10 is unexpressed in the normal colon mucosa and in most cADNs. (In rare cases, moderate expression can be observed in a few cells or crypts on the surface of cADNs.) *ANXA10* can be considered a bona fide marker of serrated glands in SSLs and HPs (Fig. 2 and Supplementary Figure 5), and it is also encountered fairly often in cells on the surface of TSAs and in their SSL-like glands.

The third gene that was highly expressed in SSLs and HPs, *ACHE* (acetylcholinesterase), is instead typically expressed in conducting tissues, including those of the enteric nervous system, and at neuromuscular junctions [48]. It terminates signal transmission by hydrolyzing the neurotransmitter acetylcholine at cholinergic synapses in the brain and at neuromuscular junctions, and pharmacologic inhibition of this enzymatic activity is used to treat colonic pseudo-obstruction [49]. We found high levels of *ACHE* mRNA in epithelial cells at the surface of the normal colorectal mucosa, but even higher levels were found in serrated glands, extending about half-way down toward the base of the crypts (Fig. 2, Supplementary Figure 6). By contrast, *ACHE* expression is markedly lower in cADNs, although it may be found in some cells on the surface of adenomatous villi. In TSAs, some serrated glands are strongly positive for *ACHE* expression, and this feature might be used to better visualize the serrated component of these polyps. *ACHE* is also expressed in some stromal cells—probably lymphocytes—and in some cells of lymphocytic folliculi. As expected, it is also strongly expressed in the submucosal plexi (Supplementary Figure 6).

Like *VISG1*, *ANXA10* and *ACHE*, *SEMG1* (Semenogelin 1) also emerged as a good marker of the serrated pathway of tumorigenesis [21, 37, 38]. It is not expressed in cADNs or in the normal colon mucosa, but moderate levels are found in SSLs and HPs, along the length of serrated crypts and to a somewhat lesser extent at the crypt bases and mouths (Fig. 3 and Supplementary Figure 7). *SEMG1* expression is also appreciable in regions of TSAs where the serrated glandular differentiation is more obvious. This gene, too, is involved in a curious form of dysregulated cell-fate differentiation that occurs during serrated tumorigenesis. *SEMG1* (like *SEMG2,* which is also strongly expressed is some SSLs [21]), is typically expressed in seminal vesicles, and the SEMG1 protein is a major component of the semen coagulum [50]. Prostate specific antigen-mediated cleavage of SEMG1 yields functional polypeptides that favor semen liquefaction and enhanced sperm motility, and increased sperm levels of SEMG1 are often associated with asthenospermia [51]. It is tempting to hypothesize that an abundant ectopic secretion of semenogelins into the lumens of serrated colon crypts might favor the formation of a tenacious mucus matrix, which would explain the presence of the adhesive mucus cap that often covers SSLs [7].

*AQP5* (Aquaporin 5) was also confirmed as a very good marker of serrated tumors: completely absent in the normal mucosa and in cADNs, *AQP5* transcript is highly or very highly expressed at the bases of all serrated crypts in SSLs and HPs (Fig. 3 and Supplementary Figure 8), and patchy, high-level expression was also observed in two of the three TSAs we investigated (TSAs 1 and 3 in Table 1). There was no evidence of *AQP5* expression in TSA 2, which was characterized by a more pervasive dysplastic, cADN-like histology than that seen in TSAs 1 and 3. Ectopic expression of this gene in serrated colorectal glands is another example of tumor-associated, phenotypic dys-differentiation. *AQP5* encodes a water-channel membrane protein normally expressed in the bronchi, salivary glands, stomach, and testis [48]. *AQP5* mutations and polymorphisms are associated with palmoplantar keratoderma [52] and with outcomes in patients with acute respiratory distress syndrome [53]. Differentiation of alveolar epithelial cells from type II to type I in the lungs is transcriptionally regulated by the p300/beta-catenin complex (but not by the CREB-binding protein/beta-catenin complex), with a concomitant increase in the expression of *AQP5* [54]. This finding suggests a functional relationship between *APQ5* and Wnt signaling, but evidently not with the canonical Wnt signaling pathway, which is constitutionally active at the base of normal colorectal crypts. The fact that *AQP5* is unexpressed in the normal colorectal mucosa and highly expressed at the bottom of serrated crypts (Fig. 3 and Supplementary Figure 8) suggests that a variant form of Wnt signaling, likely resembling that reported for alveolar epithelial cells, is active during serrated tumorigenesis. Kleeman et al. [31] reported that the Wnt signaling activation observed in CRCs arising through the serrated pathway is ligand-dependent, i.e., resulting from mutations in genes encoding RNF43 or RSPOs proteins, which amplify Wnt signal transmembrane transduction. Increased expression of *AQP5* mRNA has also been demonstrated in MMR-deficient CRCs arising via the serrated pathway [55], suggesting that such variant Wnt signaling might be upregulating the expression of this gene in the bases of serrated crypts.

In contrast, Wnt signaling activation is ligand-independent in CRCs arising along the conventional tumorigenic process acting in cADNs, i.e., tumors with mutations in *APC* or *CTNNB1* genes encoding the intracellular signal transduction proteins Adenomatous polyposis coli or β-catenin, respectively. The constitutive activation in cADNs of this canonical Wnt signaling at the base of normal colorectal crypts upregulates the expression of well-known Wnt target genes, such as *CMYC*, *CD44*, *NKD1* and *AXIN2* [31, 32]. *NKD1* (naked cuticle homolog 1) encodes a protein that negatively regulates canonical Wnt signaling via mechanisms that are still incompletely understood [56, 57]. For these reasons, we tested *NKD1* mRNA expression in this study for its potential to distinguish cADNs (where high levels were expected) from SSLs and HPs (where expression was very low and confined to a few cells at the bases of serrated crypts) (Table 1, Fig. 5 and Supplementary Figure 14).

Immunohistochemical staining patterns are sometimes difficult to interpret owing to the low specificity of the available antibodies, and this limitation would have been highly relevant for many of the targets we investigated in this study. The ISH protocol we used involves hybridization of multiple probes that are complementary to the RNA targets, thereby providing highly specific results, and its sensitivity is also high thanks to the use of a series of complementary amplification molecules (see Methods). Unlike immunohistochemistry, ISH also allows visualization within the tissue of noncoding RNAs, such as *LINC00520*, which we found to be upregulated in serrated lesions using RNA sequencing [21]. Our present findings verify the validity of *LINC00520* as a new marker of the serrated pathway: it is moderately to highly expressed in the upper half of the serrated crypts in SSLs and HPs, virtually absent in cADNs, except in a few cells at the mouth of the glands, and expressed at low to moderate levels at the surface of normal crypts (Table 1, Fig. 3 and Supplementary Figure 9). Therefore, like *ACHE*, the *LINC00520* gene is normally expressed in the superficial epithelium of normal colorectal crypts, and this expression is markedly upregulated in serrated lesions, where it extends deep into the abnormal crypts. This long noncoding RNA regulates endothelial nitric oxide synthase expression [58] and may play role in breast tumorigenesis [59], but its epigenetic regulatory function in the colorectal epithelium is completely unknown.

Two of the serrated-specific targets investigated in this study encode transcription factors, *ZIC5* (Zinc finger protein of the cerebellum) and *FOXD1* (Forkhead box D1). They are essential for embryonic development of specific tissues [60, 61] but absent in most adult tissues, including the normal intestinal mucosa. Developmental transcription factors like these are often found to be ectopically re-expressed in specific tumor cells, and this is the case for *ZIC5* and *FOXD1* in serrated colorectal tumor cells (Table 1, Fig. 4 and Supplementary Figures 10 and 11). Their mRNAs were consistently present in the serrated lesions we investigated: *FOXD1* labeling was observed along the entire longitudinal axis of serrated crypts, whereas *ZIC5* was generally confined to the lower half*. ZIC5* and *FOXD1* expression levels in serrated lesion were both low, probably because their mRNAs (like those of most transcription factors) are relatively unstable [62].

Interestingly, *ZIC2* and its neighbor, *ZIC5,* displayed the same staining patterns in serrated lesions (Supplementary Figure 10). Expression of these two genes inhibits the transcriptional activity of beta-catenin/TCF (i.e., the canonical Wnt signaling that occurs in the adult stem cell compartment of the intestinal epithelium), thereby disrupting intestinal epithelial homeostasis [63]. Their re-expression during serrated tumorigenesis once again points to a switch from the canonical Wnt signaling active during conventional adenomatous tumorigenesis to a fundamentally different variant form of this signaling cascade, as previously discussed for *AQP5* and *NKD1*. Indeed, ZIC5/2 expression has been reported in *APC*-wildtype and MMR-deficient colon cancer cell lines, but levels were almost undetectable in *APC*-mutant and MMR-proficient lines [63].

RNA-sequencing data [21] revealed SSL-specific upregulation of *APOBEC1* (Apolipoprotein B mRNA editing catalytic subunit 1) and, as reported by others [37, 44], *MUC5AC* (Mucin 5 AC) expression (in comparison with normal mucosa). Topographical analysis of the ISH tissue-staining patterns confirmed the RNA-sequencing data, but it also highlighted a risk of error associated with conclusions based exclusively on this type of data. As shown in Table 1, Figs. 4 and 5, and Supplementary Figures 12 and 13, neither *APOBEC1* nor *MUC5AC* can be considered a bona fide marker of the serrated pathway: both are more strongly expressed in SSLs than they are in cADNs, but cADNs do consistently express both genes, albeit in more restricted areas of the glands or in a patchy pattern. (These staining patterns explain why random selection of endoscopic biopsies for RNA extraction and sequencing can lead to underestimated expression levels of certain genes in some tumor types.)

The RNA-editing enzyme APOBEC1, which deaminates apolipoprotein B mRNA Cytosine666 > Uracil in the small intestine [64], is moderately expressed in the surface epithelium of the colorectal mucosa (Supplementary Figure 12). Its high-level expression in SSLs extends down into the serrated crypts but stops short of the crypt base. It remains to be seen whether apolipoprotein B editing and/or APOBEC1-mediated DNA mutagenesis (i.e., C > T transitions stemming from unrepaired cytosine deaminations) are increased in these neoplastic crypts [65, 66]. It is interesting to note that C > T transitions at CpG dinucleotides are over-represented in the DNA mutation signature of CRCs displaying MMR-deficiency [67, 68], which, as discussed above, is caused by CIMP-mediated silencing of *MLH1* expression.

As for *MUC5AC,* its tumor-associated expression represents another example of dysregulated neoplastic cell-fate differentiation. *MUC5AC* encodes a typical gel-forming glycoprotein found in normal gastric and respiratory tract epithelial cells [69], and it proved to be an excellent marker of goblet cells in all the tumors we investigated (especially SSLs, which are typically goblet-cell-rich) but not of the goblet cells found in the normal colorectal mucosa (Supplementary Figure 13). The goblet-cell differentiation that occurs in serrated lesions (and in some areas of cADNs and TSAs) thus appears to be epigenetically distinct from that seen in normal colorectal crypts.

Whole-section analysis with ISH or immunohistochemistry facilitates characterization of expression pattern heterogeneity within a tumor, a feature that can be missed with RNA sequencing analysis of random biopsies, as exemplified by our experience with *MUC5AC* and *APOBEC1*. When used with reliable antibodies, immunohistochemistry can also identify tumor-specific changes that can escape detection by ISH. A recent example involves AGRN protein expression in the muscularis mucosae of SSLs, which has been shown to distinguish SSLs from HPs despite the fact that the two lesions display similar levels of *AGRN* mRNA expression levels in the lower half of their serrated crypts [70] (see also Supplementary Figure 2 and 3**:** *AGRN* mRNA expression patterns from [21, 38], respectively).

Nine targets were chosen as putative HP-specific tissue staining markers [38] (RNA-sequencing data shown in Supplementary Figure 3), but none of the nine were verified as such by our ISH findings. Table 1, Fig. 6 and Supplementary Figures 15 and 16 show two examples, the *HOXD13* and *HOXB13* genes. They belong to two different homeobox gene families of transcription factors that play crucial roles in vertebrate embryonic development [71, 72]. Their expression in adult tissues is restricted to the distal colon and prostate (both genes), the vagina (*HOXD13*), and the urinary bladder (*HOXB13*) [48]. ISH confirmed that *HOXD13* is expressed in the normal mucosa of the distal colon, the rectum in particular. It is also expressed at crypt bases in HPs, especially those located in the rectum (Table 1), but low expression is detectable also in the other tumor types investigated. Like *HOXD13, HOXB13* expression is restricted to the distal colon and rectum (generally at higher levels than *HOXD13*), and it is expressed in HPs as well as all other tumor types, with levels in distal-colon tumors that are far higher than those in their proximal-colon counterparts. These two genes are more appropriately considered bona fide markers of the normal epithelium of the distal colon and rectum rather than of HPs. As discussed above, the fact that HPs are much more likely to arise in these segments than SSLs probably explains why these genes would appear to be HP-specific on the basis of RNA-sequencing data.

*FAM3B*, which encodes a signaling protein normally expressed in the endocrine pancreas and gastrointestinal tract [48, 73], was chosen as a putative negative marker of HPs, i.e., one whose nonexpression is specific to these lesions (Supplementary Figure 3). This assumption was also explained by the staining pattern of the normal mucosa: unlike the previously discussed mRNAs, *FAM3B* is expressed only in the proximal segments of the normal colon, where HPs are rare. It was also variably expressed in the other lesion types from all colorectal segments, but the highest levels were found in tumor glands of proximal-colon lesions (Fig. 6, Supplementary Figure 17).

The search for markers that can clearly distinguish SSLs from HPs is obviously going to be difficult. Kanth et al. [38] have identified additional candidate markers that were not included in our investigation. Using RT-PCR, they recently assessed the performance of a 7-marker panel that included five of those candidates, as well as *SEMG1* and *ZIC5/2.* The panel differentiated SSLs from HPs with 89% sensitivity and 88% specificity [74]. However, the gene expression differences between these two types of serrated tumors were less significant when distal- rather than proximal-colon SSLs were considered, suggesting that distinguishing between serrated lesions arising in the same colon segment is still likely to be problematic.

One of the obvious limitations of our study is that it was conducted on an undeniably small number of colorectal lesions. Our aim here was to verify biomarker candidates on the basis of our previously reported RNA-sequencing data. Becasue the ISH protocol entails synthesis of branched DNA molecules, it is expensive, at least when done manually, as it was in this study, where the priority was to test a relatively large number of promising markers instead of evaluating a few markers in numerous lesions. However, this technique could easily be used with the automatic robotic instruments routinely used for immunohistochemistry in all pathology laboratories. This would reduce costs considerably and greatly facilitate next-step efforts to provide the reliable validation of the most promising markers in larger series of colorectal tumors representing all histologic types, sizes, and colorectal segments of origin.

Descriptive findings like ours can clearly have impact in the clinics, but they can (and should) also serve as springboards for research into the functional significance during serrated colorectal tumorigenesis of the dysregulated gene expression discussed above. The cascade of molecular events that characterizes this process appears to involve a dramatic epigenetic reprogramming, whose early stages are reflected by the recently described proto-CIMP phenotype [21] and intriguing forms of aberrant differentiation at the cellular and tissue levels.

## Supplementary Information

**Additional file 1: Supplementary Figure 1.** In situ hybridization experiments: positive and negative controls. Staining controls included mRNA of the human housekeeping gene *PPIB* (positive control; the punctate labeling in this panel is brown since a different chromogen was used for this hybridization) (A), the bacterial RNA *DapB* (negative control) (B), and the long noncoding RNA of the X chromosome-located gene *XIST* (control for tissue-donor sex: positive for female, negative for male, panels C and D, respectively, and Table 1).

**Additional file 2: Supplementary Figure 2.** (Panels A through O). RNA-sequencing-based expression profiles of the targets included in this study based on data published by Parker et al. (reference [21]). Integrative Genomics Viewer snapshots are shown for the serrated lesions and cADNs investigated by Parker et al. All lesions assessed in this study were from the *proximal* colon (details in reference [21]). Seventeen SSLs are compared with 15 cADNs, and *each track* in the snapshot shows the level of a given RNA (i.e., peaks across exons proportional to the number of sequencing reads) in the lesion (SSLs: red track: cADNs: blue track). Below each of these tracks is a track showing the expression level of the same RNA in a patient-matched sample of normal mucosa from the proximal colon (i.e., cecum, ascending, hepatic flexure or transversum) harboring the precancerous lesion (pink track: normal mucosa of an SSL carrier; light blue track: normal mucosa of a cADN carrier).

**Additional file 3: Supplementary Figure 3.** (Panels A through K). RNA-sequencing-based expression profiles of the targets included in this study based on data published by Kanth et al. (reference [38]). Clinical data on the lesions and normal mucosa samples investigated by Kanth et al. are in panel A, while the RNA expression level (i.e., logCPM: log2 counts per million) of the targets in each tissue is graphically shown in the following panels.

**Additional file 4: Supplementary Figure 4.** In situ hybridization analysis of *VSIG1* expression in serrated precursor lesions, cADNs, and normal colorectal mucosa. *VSIG1* expression is a bona fide marker of the crypt serration found in SSLs and HPs (A-D), where very high levels (according to the scoring system depicted in Fig. 1) are present along the entire longitudinal axis of the serrated crypts, except the bases and mouths. The three TSAs included in this study were largely *VSIG1*-negative (E), but patchy staining of SSL-type glands within two of these lesions was noted (F) (TSA 3, shown here, and TSA 1, as reported in Table 1). cADNs (G) and normal mucosa (H) were negative. Lesions are numbered as in Table 1.

**Additional file 5: Supplementary Figure 5.** In situ hybridization analysis of *ANXA10* expression in serrated precursor lesions, cADNs, and normal colorectal mucosa. *ANXA10* is a specific marker of SSLs (A and B) and HPs (Table 1), where its expression pattern is similar to that of *VSIG1* (Supplementary Figure 4). High expression is also seen in a few glands in TSAs, on the surfaces of these lesions, or distributed in irregular patches (C, D and E, Table 1). Isolated positive cells or glands can also be seen very rarely on the surface of cADNs (F and G), but the normal mucosa is consistently negative (H).

**Additional file 6: Supplementary Figure 6.** In situ hybridization analysis of *ACHE* expression in serrated precursor lesions, cADNs, and normal colorectal mucosa. In SSLs (A and B) and HPs (C) (Table 1), *ACHE* is very highly expressed at the lesion surface and in the upper half of the serrated crypts. Numerous *ACHE*-positive crypts are also seen on the surfaces of TSAs, especially in TSA 3 (F and G, Table 1). cADNs are negative with the exception of a few cells with low-to-moderate expression on the surface of adenomatous villi (D and E). Moderate to high *ACHE* expression was observed in the superficial epithelium of normal crypts (H), submucosa plexi (inset in panel H), some stromal cells (example in panel E), and in lymphocytic folliculi (not shown).

**Additional file 7: Supplementary Figure 7.** In situ hybridization analysis of *SEMG1* in serrated precursor lesions, cADNs and normal colorectal mucosa. *SEMG1* is moderately expressed in SSLs and HPs (A-D) along most of the longitudinal axis of serrated crypts, with lower-level expression at their bases and mouths (Table 1). Patches of low expression were also seen in TSAs (E and F). In cADNs and normal mucosa, *SEMG1* was virtually absent (G and H, respectively) with the exception of a few cells with one or two dot-like signals each reflecting a single RNA molecule.

**Additional file 8: Supplementary Figure 8.** In situ hybridization analysis of *AQP5* in serrated precursor lesions, cADNs and normal colorectal mucosa. *AQP5* is also a bona fide marker of serrated tumors: it is very highly expressed in the lower half of serrated crypts in SSLs and HPs (A-C) and in 2 of the 3 TSAs we analyzed (D-F), but no expression was observed in cADNs (G) or normal mucosa (H) (Table 1).

**Additional file 9: Supplementary Figure 9.** In situ hybridization analysis of *LINC00520* in serrated precursor lesions, cADNs and normal colorectal mucosa. The long noncoding *LINC00520* RNA is also a good marker of serrated crypts in SSLs (A and B) and HPs (C and D), where it is moderately but consistently expressed in their upper half (Table 1). It is essentially absent in cADNs (E) and TSAs (F) with the exception of rare cells with low expression at the surfaces of these lesions and a few positive SSL-like glands in TSA 3 (G). Moderate expression was also observed in the uppermost epithelial layer of the normal colorectal mucosa (H).

**Additional file 10: Supplementary Figure 10.** In situ hybridization analysis of *ZIC5 and ZIC2* in serrated precursor lesions, cADNs and normal colorectal mucosa. *ZIC5* and *ZIC2* are neighboring transcription factor-encoding genes with similar expression profiles in the colorectal tissues investigated (Supplementary Figure 2G). They are consistently expressed at low levels at the bases of serrated crypts in SSLs and HPs (A, B, and F) (Table 1). Neither gene is expressed in cADNs (D), the normal mucosa (E), or TSAs (C) with the exception of a few SSL-like glands in the latter lesions (Table 1). The brown (instead of red) punctate labeling in panel F reflects the use of a different chromogen from that used in other hybridizations.

**Additional file 11: Supplementary Figure 11.** In situ hybridization analysis of *FOXD1* in serrated precursor lesions, cADNs and normal colorectal mucosa. *FOXD1* is another marker of SSLs and HPs, where it is lowly expressed along the entire length of the serrated crypts (A-C). Limited areas of positivity were observed in 2 of the 3 TSAs (D and E) (Table 1), but no expression was found in cADNs (F and G) or in normal mucosa (H).

**Additional file 12: Supplementary Figure 12.** In situ hybridization analysis of *APOBEC1* in serrated precursor lesions, cADNs and normal colorectal mucosa. *APOBEC1* was expressed in all the lesion types investigated, especially in the upper portions of glands, and also in the superficial epithelium of the normal mucosa (H) (Table 1). However, the expression was higher in SSLs, where it was absent only at the bases of serrated glands (A and B), and in HPs, where high expression was more confined to the surface of the lesions (Table 1). In TSAs and cADNs, moderate expression of *APOBEC1* was seen in glands with more evident goblet-cell differentiation (C-G).

**Additional file 13: Supplementary Figure 13.** In situ hybridization analysis of *MUC5AC* in serrated precursor lesions, cADNs and normal colorectal mucosa. *MUC5AC* expression, like that of *APOBEC1*, is not specific to serrated lesions. Very high levels were found in the mucous cells of all the lesions we tested, but not in those of the normal mucosa (H) (excluding rare positive cells in its surface; not shown). Very high *MUC5AC* expression was more extensive in SSLs and HPs (i.e., along the entire length of serrated crypts with lower levels only at their bases) (A-C), while patchier expression was observed in cADNs (D and E) and TSAs (F and G) (Table 1). *MUC5AC* therefore represents a marker of neoplastic goblet cells (e.g., panel E) but not of their normal mucosal counterparts (panel H).

**Additional file 14: Supplementary Figure 14.** In situ hybridization analysis of *NKD1* in serrated precursor lesions, cADNs and normal colorectal mucosa. *NKD1* is expressed only in a few cells within the stem-cell compartment at the bottom of serrated crypts in SSLs (A) and HPs (B) and at the bases of normal mucosal crypts (I). In cADNs, it is very highly and extensively expressed, with patchy variation of intensity (C and D), and similarly high expression was also observed in one of the TSAs (E and F). Its expression is much more limited in the other two TSAs (G and H, and Table 1). *NKD1* is also expressed in some stromal cells and quite extensively in the muscularis mucosae (I).

**Additional file 15: Supplementary Figure 15.** In situ hybridization analysis of *HOXD13* in serrated precursor lesions, cADNs and normal colorectal mucosa. In proximal-colon SSLs, *HOXD13* is expressed only in a few cells at crypt bases (A) (Table 1). The two distal-colon HPs (especially HP 3, which arose in the rectum) displayed moderate expression at the crypt bases (C). Patchy, low-to-moderate *HOXD13* expression was also present in cADNs and TSAs (B and D), and only a few sporadic positive crypts were noted in the normal mucosa of the proximal colon (E and F). In contrast, it was expressed in the normal mucosa of the distal colon and rectum at moderate levels, although patches of negative crypts were also seen (G and H).

**Additional file 16: Supplementary Figure 16.** In situ hybridization analysis of *HOXB13* in serrated precursor lesions, cADNs and normal colorectal mucosa. *HOXB13* was generally more highly expressed than *HOXD13* in colorectal tissues (Table 1; see also Supplementary Figures 2 and 3), but, similarly to *HOXD13*, it is a typical marker of the normal mucosal of the distal colon and rectum (H). In the normal mucosa of the proximal colon, expression was low or absent (G). Moderate-to-high *HOXB13* expression was found in SSLs (A), HPs (B), TSAs (E and F), and cADNs (C and D), but in all four lesion types, more abundant expression was found in tumors taken from the distal colon and rectum (Table 1).

**Additional file 17: Supplementary Figure 17.** In situ hybridization analysis of *FAM3B* in serrated precursor lesions, cADNs, and normal colorectal mucosa. In contrast to *HOXD13* and *HOXB13* of the two previous supplementary figures, *FAM3B* is highly expressed in the normal epithelium of the proximal colon (C and G) but unexpressed in the distal colon (H) (Table 1; see also Supplementary Figures 2 and 3). Moderate and localized (patchy or superficial) staining was seen in all tumors regardless of type and colorectal segment of origin (A, B, D, E, and F), although higher levels were often found in lesions from the proximal colon.

**Additional file 18: Supplementary Figures 18–29.** H&E-stained sections of each of the 12 lesions investigated in this study.

**Additional file 19: Supplementary Table 1.** mRNA targets chosen for ISH based on RNA sequencing data from Parker H. et al (ref. [21]) and Kanth P. et al (ref. [38]).

## Data Availability

Not applicable.

## Abbreviations

cADNs: Conventional adenomas
CRC: Colorectal cancer
CIMP: CpG island methylator phenotype
H&E: Hematoxylin and eosin
HP: Hyperplastic polyp
ISH: In situ hybridization
MMR: DNA mismatch repair
SSL: Sessile serrated lesions
TSA: Traditional serrated adenoma
