# Supplementary figures and images for: An “expressionistic” look at serrated precancerous colorectal lesions
Source: Diagn Pathol. 2021 Jan 10;16:4. doi: 10.1186/s13000-020-01064-1 (PMC7797135; doi:10.1186/s13000-020-01064-1)

**A**

**PPIB**

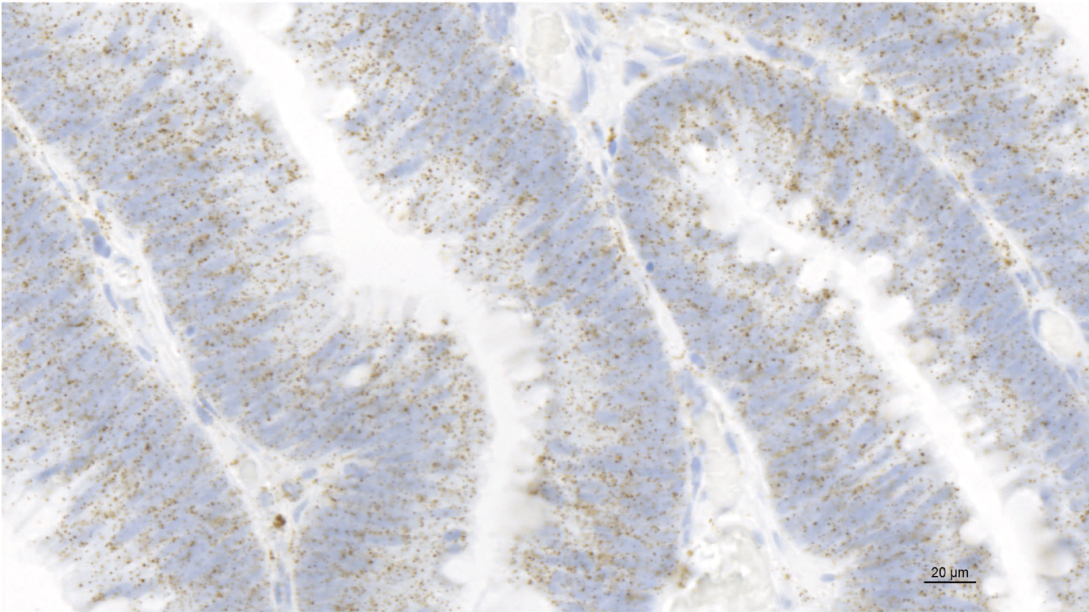

**B**

**DapB**

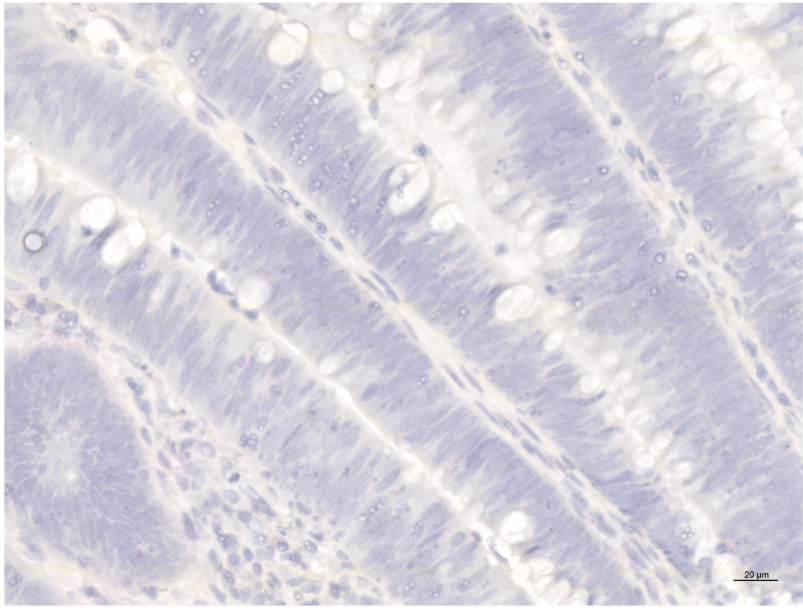

**C**

**XIST**

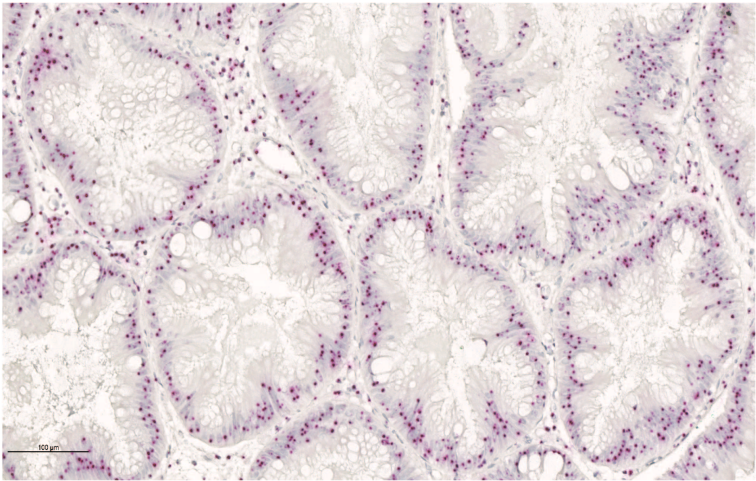

female

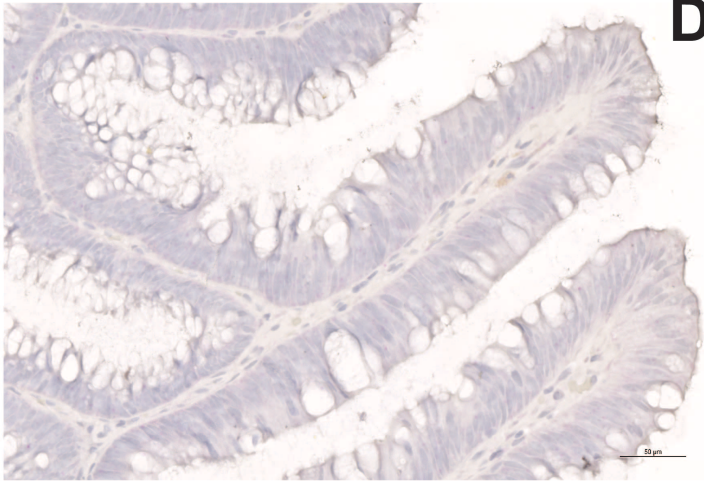

male

**D**

Supplement: Supplementary file 1 — Additional file 1: Supplementary Figure 1. In situ hybridization experiments: positive and negative controls. Staining controls included mRNA of the human housekeeping gene PPIB (positive control; the punctate labeling in this panel is brown since a different chromogen was used for this hybridization) (A), the bacterial RNA DapB (negative control) (B), and the long noncoding RNA of the X chromosome-located gene XIST (control for tissue-donor sex: positive for female, negative for male, panels C and D, respectively, and Table 1). [file 13000_2020_1064_MOESM1_ESM.pdf]

***VSIG1***

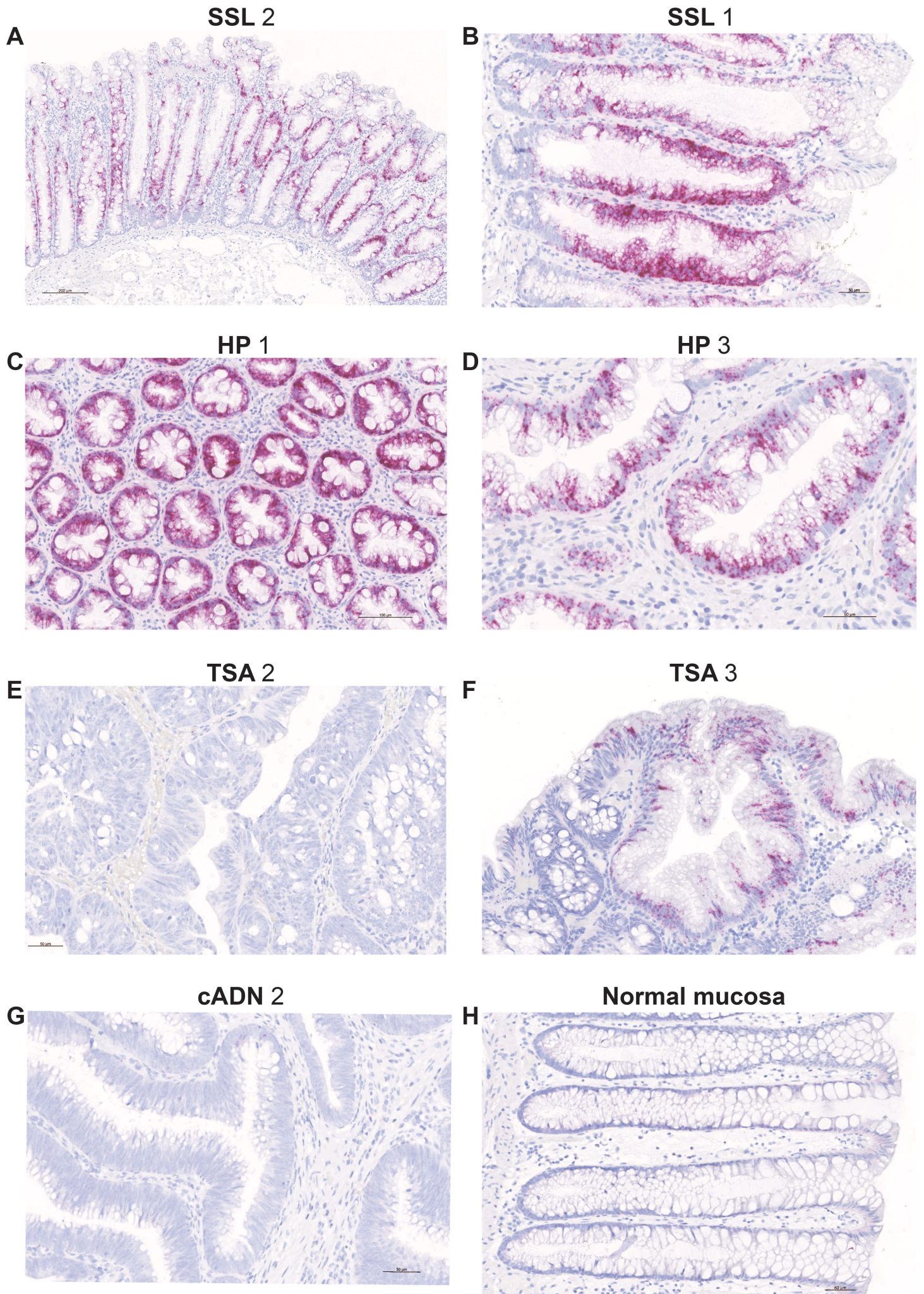

Supplement: Supplementary file 4 — Additional file 4: Supplementary Figure 4. In situ hybridization analysis of VSIG1 expression in serrated precursor lesions, cADNs, and normal colorectal mucosa. VSIG1 expression is a bona fide marker of the crypt serration found in SSLs and HPs (A-D), where very high levels (according to the scoring system depicted in Fig. 1) are present along the entire longitudinal axis of the serrated crypts, except the bases and mouths. The three TSAs included in this study were largely VSIG1-negative (E), but patchy staining of SSL-type glands within two of these lesions was noted (F) (TSA 3, shown here, and TSA 1, as reported in Table 1). cADNs (G) and normal mucosa (H) were negative. Lesions are numbered as in Table 1. [file 13000_2020_1064_MOESM4_ESM.pdf]

## *ANXA10*

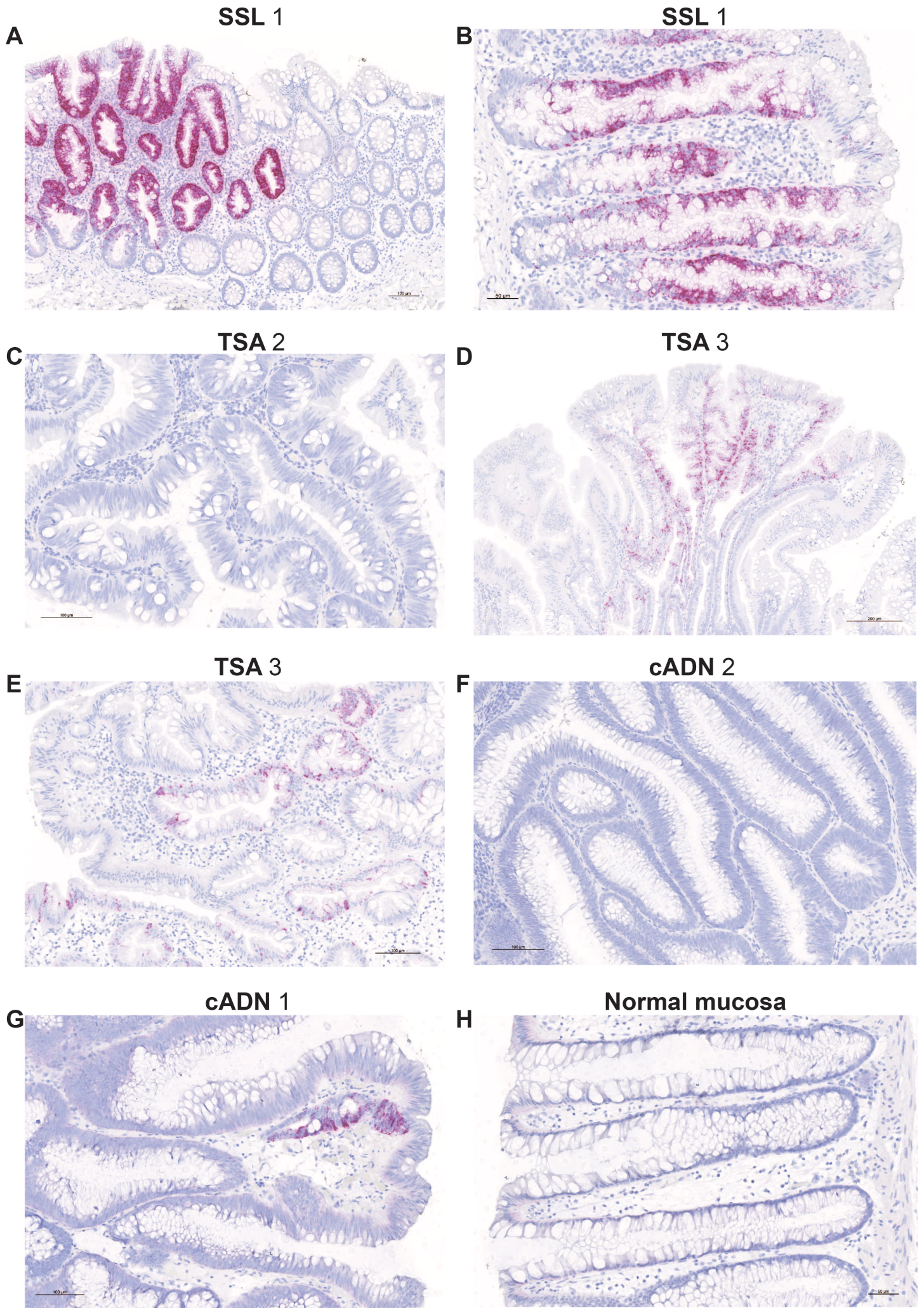

Supplement: Supplementary file 5 — Additional file 5: Supplementary Figure 5. In situ hybridization analysis of ANXA10 expression in serrated precursor lesions, cADNs, and normal colorectal mucosa. ANXA10 is a specific marker of SSLs (A and B) and HPs (Table 1), where its expression pattern is similar to that of VSIG1 (Supplementary Figure 4). High expression is also seen in a few glands in TSAs, on the surfaces of these lesions, or distributed in irregular patches (C, D and E, Table 1). Isolated positive cells or glands can also be seen very rarely on the surface of cADNs (F and G), but the normal mucosa is consistently negative (H). [file 13000_2020_1064_MOESM5_ESM.pdf]

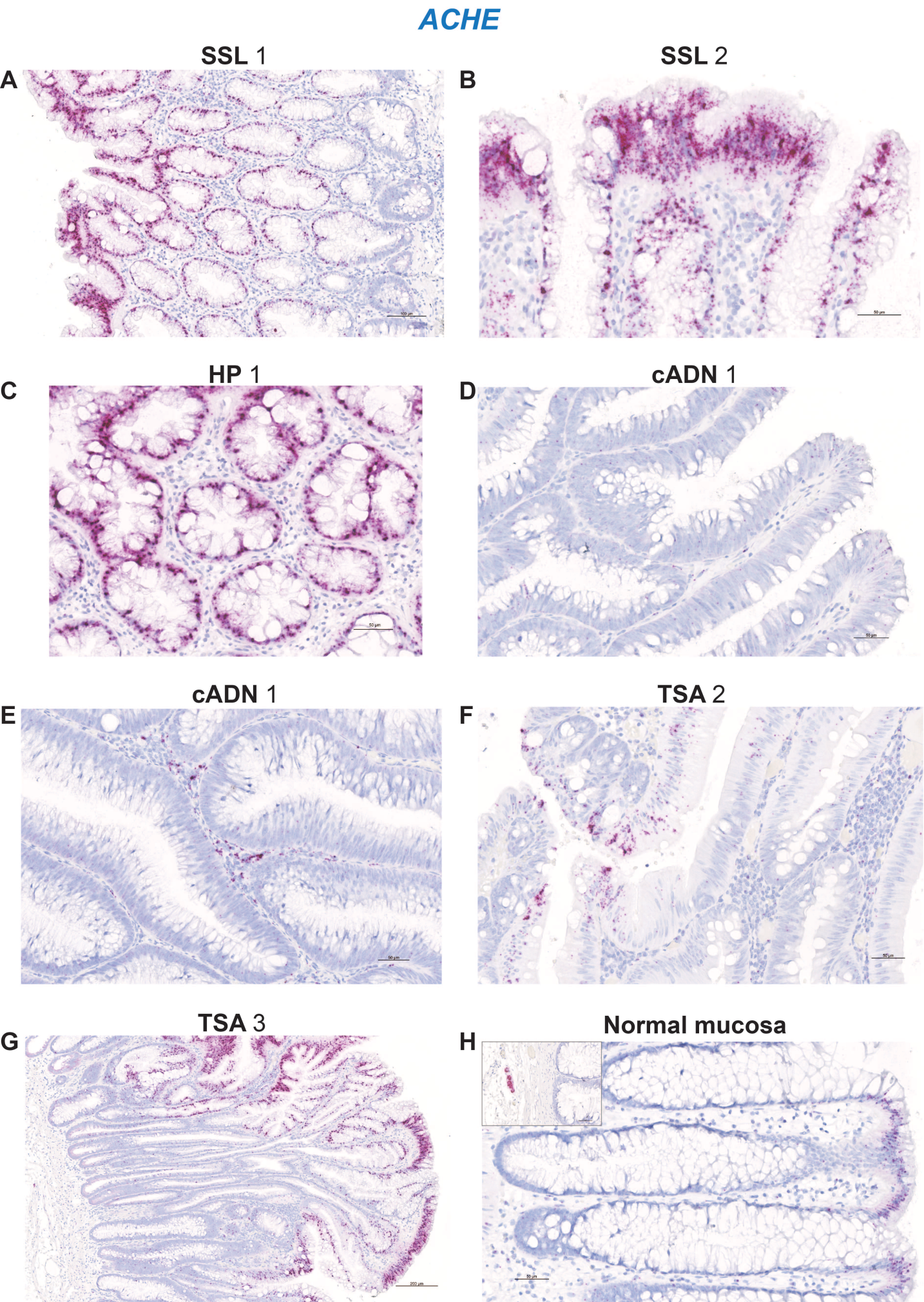

Supplement: Supplementary file 6 — Additional file 6: Supplementary Figure 6. In situ hybridization analysis of ACHE expression in serrated precursor lesions, cADNs, and normal colorectal mucosa. In SSLs (A and B) and HPs (C) (Table 1), ACHE is very highly expressed at the lesion surface and in the upper half of the serrated crypts. Numerous ACHE-positive crypts are also seen on the surfaces of TSAs, especially in TSA 3 (F and G, Table 1). cADNs are negative with the exception of a few cells with low-to-moderate expression on the surface of adenomatous villi (D and E). Moderate to high ACHE expression was observed in the superficial epithelium of normal crypts (H), submucosa plexi (inset in panel H), some stromal cells (example in panel E), and in lymphocytic folliculi (not shown). [file 13000_2020_1064_MOESM6_ESM.pdf]

**SEMG1**

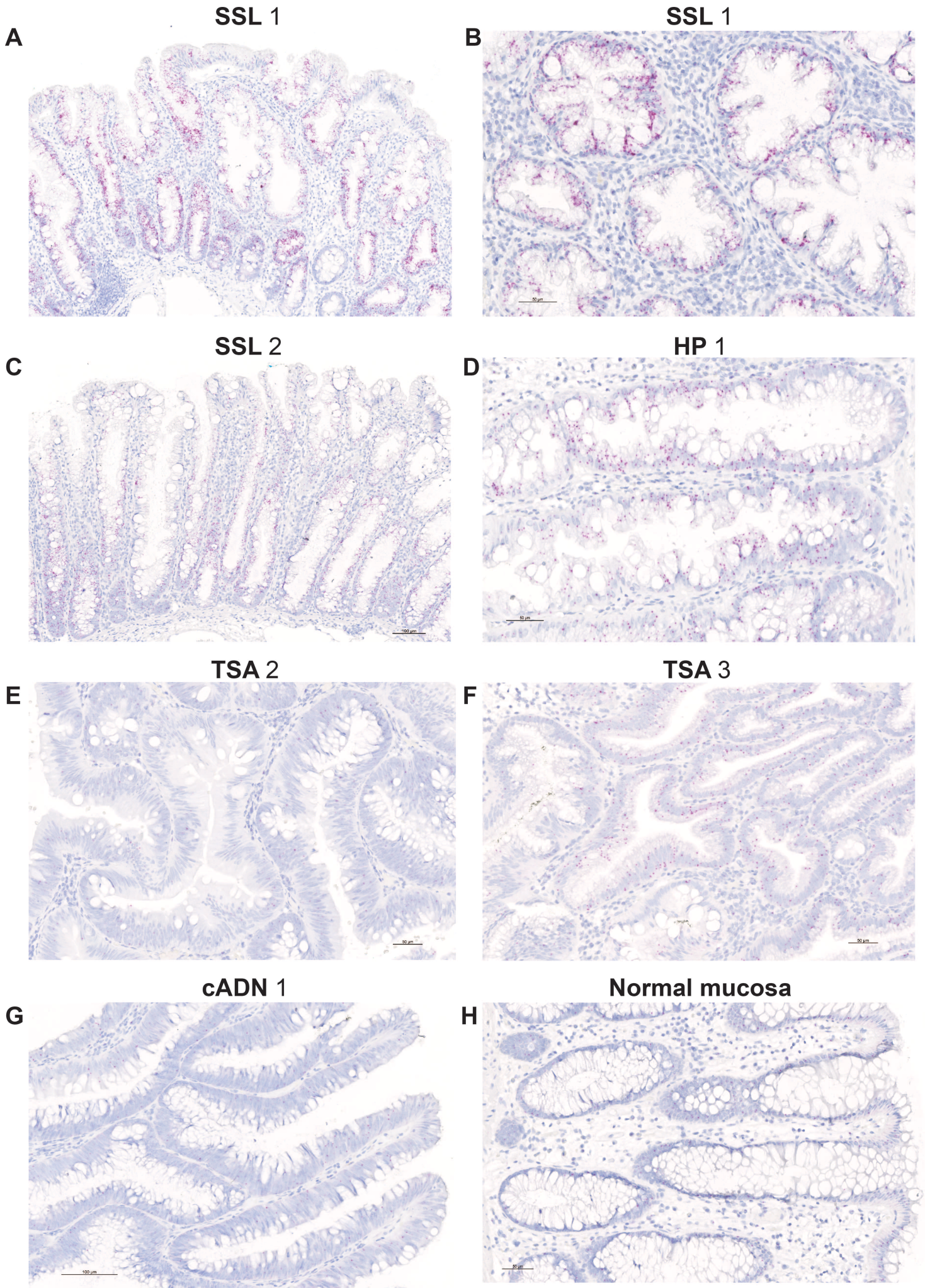

Supplement: Supplementary file 7 — Additional file 7: Supplementary Figure 7. In situ hybridization analysis of SEMG1 in serrated precursor lesions, cADNs and normal colorectal mucosa. SEMG1 is moderately expressed in SSLs and HPs (A-D) along most of the longitudinal axis of serrated crypts, with lower-level expression at their bases and mouths (Table 1). Patches of low expression were also seen in TSAs (E and F). In cADNs and normal mucosa, SEMG1 was virtually absent (G and H, respectively) with the exception of a few cells with one or two dot-like signals each reflecting a single RNA molecule. [file 13000_2020_1064_MOESM7_ESM.pdf]

# **AQP5**

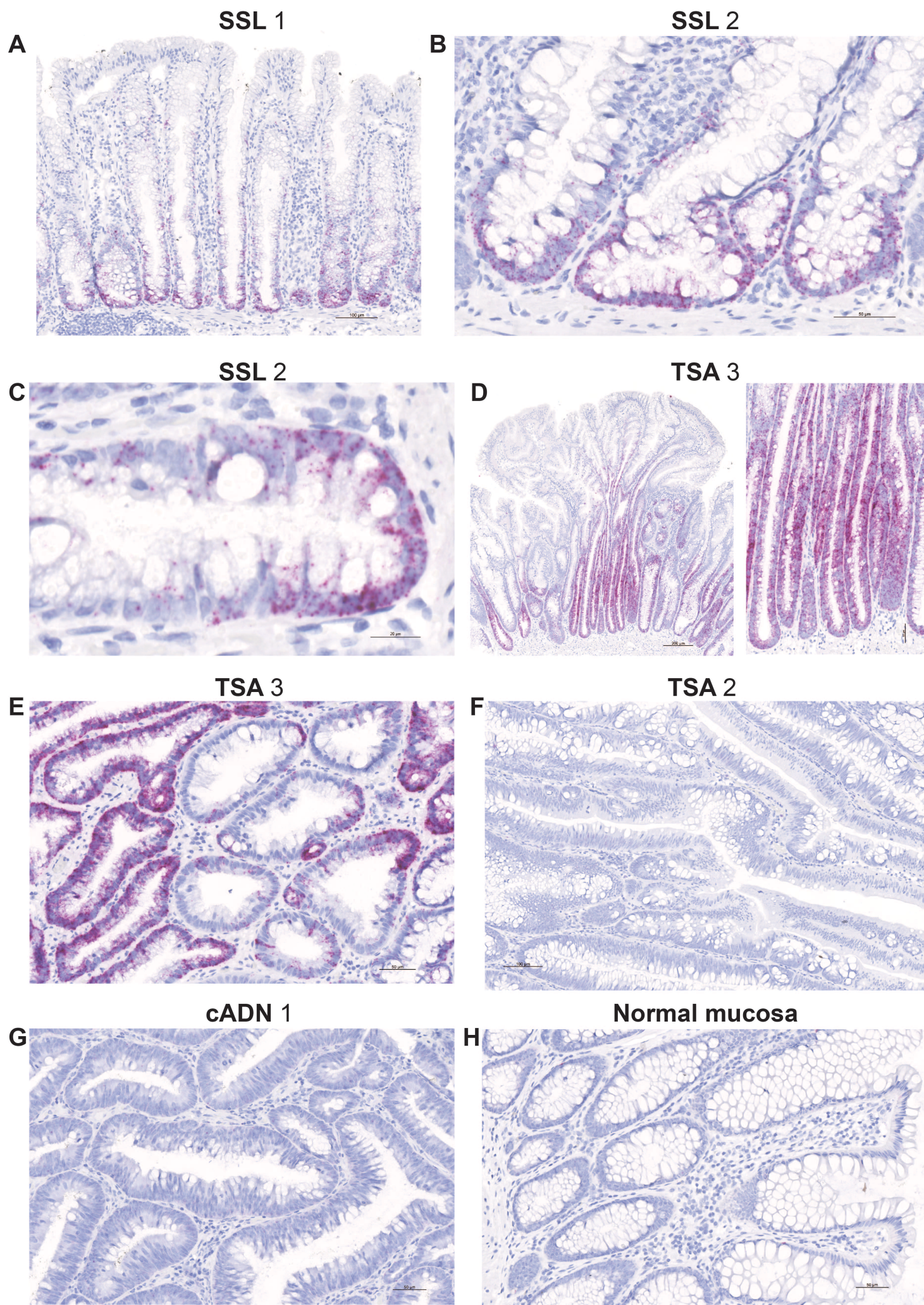

Supplement: Supplementary file 8 — Additional file 8: Supplementary Figure 8. In situ hybridization analysis of AQP5 in serrated precursor lesions, cADNs and normal colorectal mucosa. AQP5 is also a bona fide marker of serrated tumors: it is very highly expressed in the lower half of serrated crypts in SSLs and HPs (A-C) and in 2 of the 3 TSAs we analyzed (D-F), but no expression was observed in cADNs (G) or normal mucosa (H) (Table 1). [file 13000_2020_1064_MOESM8_ESM.pdf]

***LINC00520***

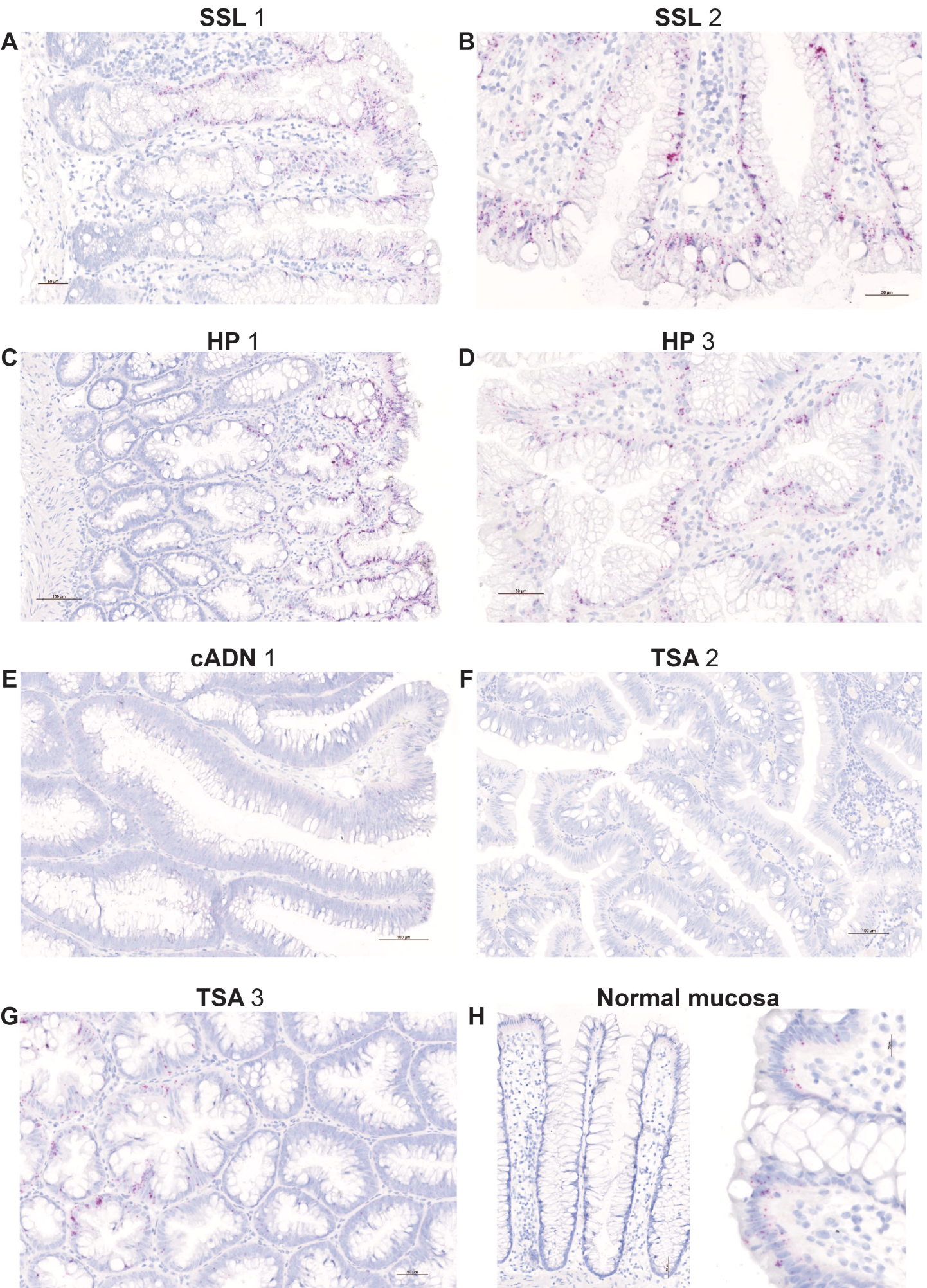

Supplement: Supplementary file 9 — Additional file 9: Supplementary Figure 9. In situ hybridization analysis of LINC00520 in serrated precursor lesions, cADNs and normal colorectal mucosa. The long noncoding LINC00520 RNA is also a good marker of serrated crypts in SSLs (A and B) and HPs (C and D), where it is moderately but consistently expressed in their upper half (Table 1). It is essentially absent in cADNs (E) and TSAs (F) with the exception of rare cells with low expression at the surfaces of these lesions and a few positive SSL-like glands in TSA 3 (G). Moderate expression was also observed in the uppermost epithelial layer of the normal colorectal mucosa (H). [file 13000_2020_1064_MOESM9_ESM.pdf]

**ZIC5**

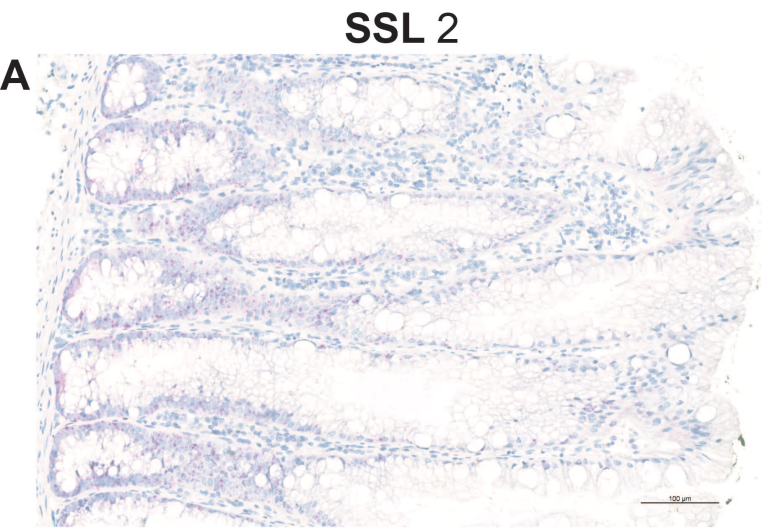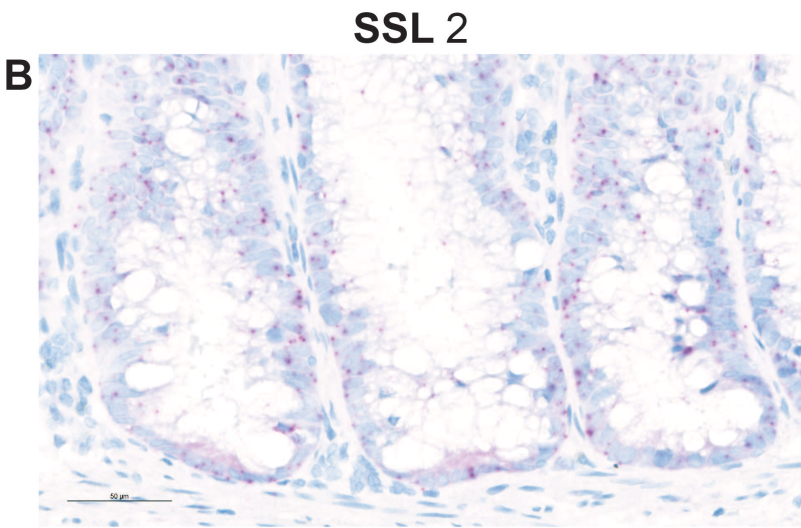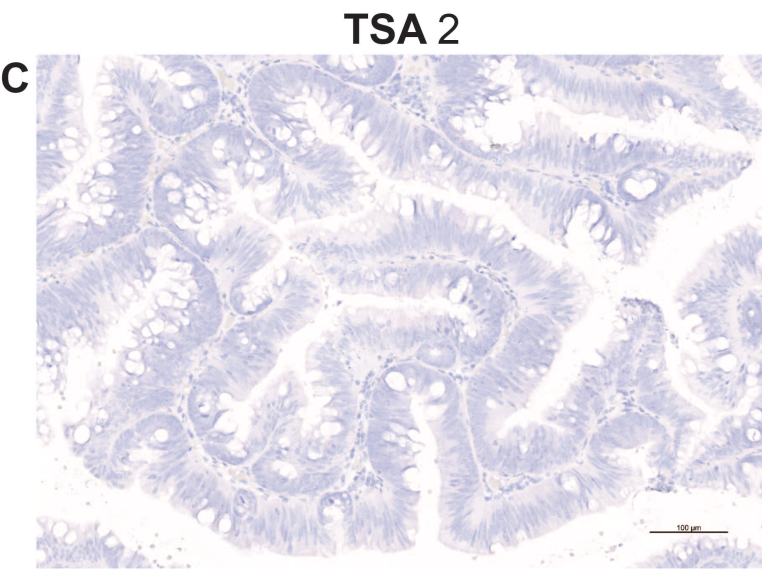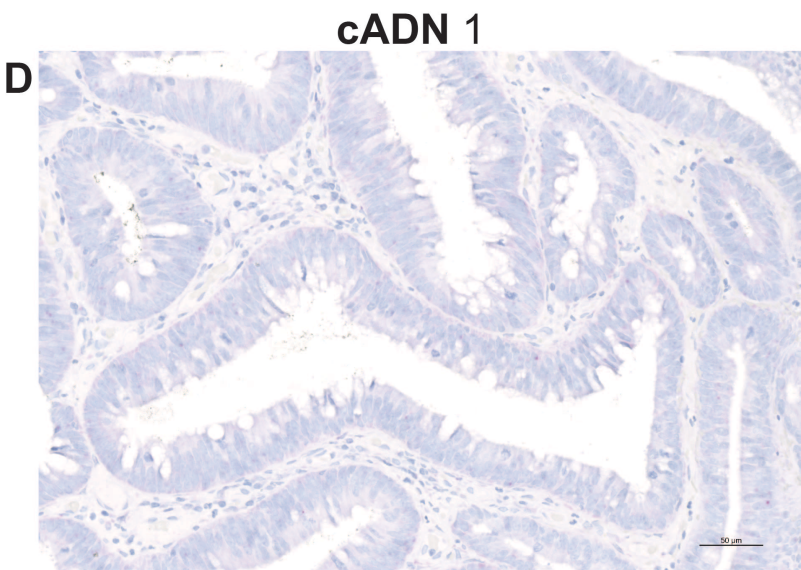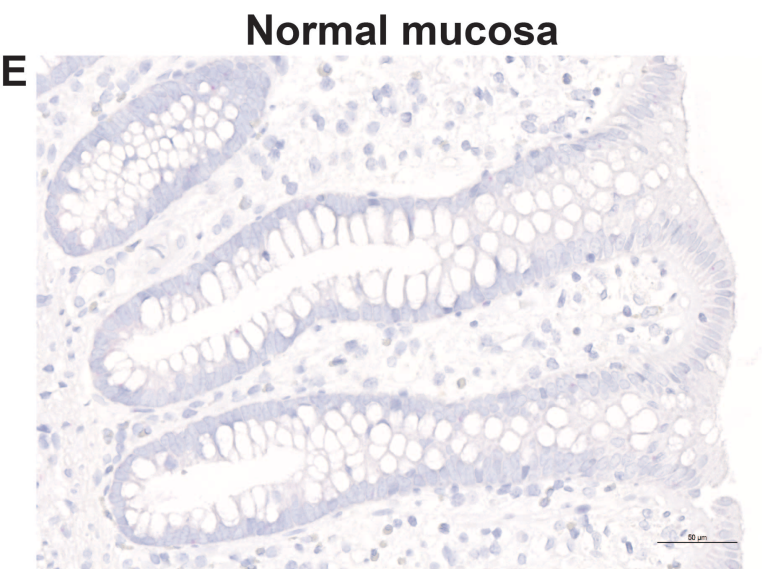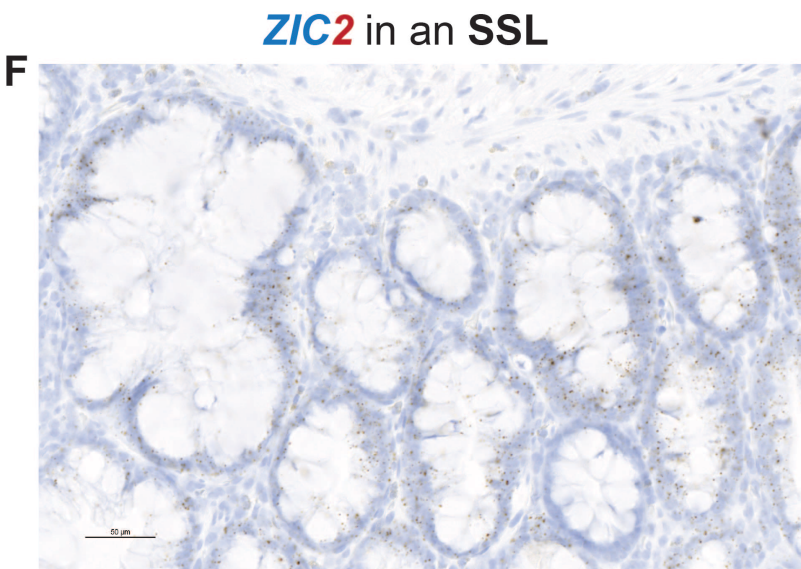

Supplement: Supplementary file 10 — Additional file 10: Supplementary Figure 10. In situ hybridization analysis of ZIC5 and ZIC2 in serrated precursor lesions, cADNs and normal colorectal mucosa. ZIC5 and ZIC2 are neighboring transcription factor-encoding genes with similar expression profiles in the colorectal tissues investigated (Supplementary Figure 2G). They are consistently expressed at low levels at the bases of serrated crypts in SSLs and HPs (A, B, and F) (Table 1). Neither gene is expressed in cADNs (D), the normal mucosa (E), or TSAs (C) with the exception of a few SSL-like glands in the latter lesions (Table 1). The brown (instead of red) punctate labeling in panel F reflects the use of a different chromogen from that used in other hybridizations. [file 13000_2020_1064_MOESM10_ESM.pdf]

# *FOXD1*

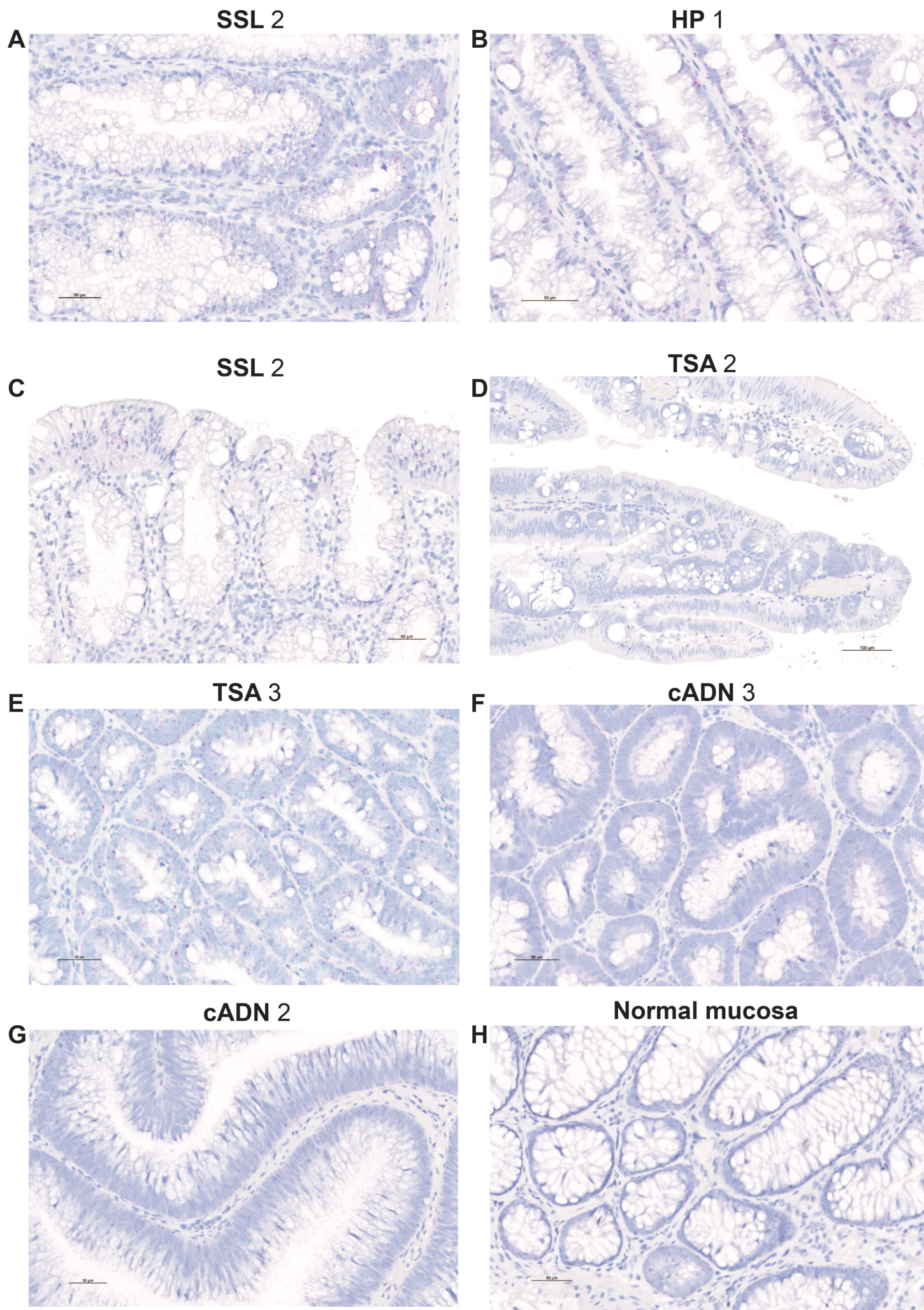

Supplement: Supplementary file 11 — Additional file 11: Supplementary Figure 11. In situ hybridization analysis of FOXD1 in serrated precursor lesions, cADNs and normal colorectal mucosa. FOXD1 is another marker of SSLs and HPs, where it is lowly expressed along the entire length of the serrated crypts (A-C). Limited areas of positivity were observed in 2 of the 3 TSAs (D and E) (Table 1), but no expression was found in cADNs (F and G) or in normal mucosa (H). [file 13000_2020_1064_MOESM11_ESM.pdf]

***APOBEC1***

**SSL 1**

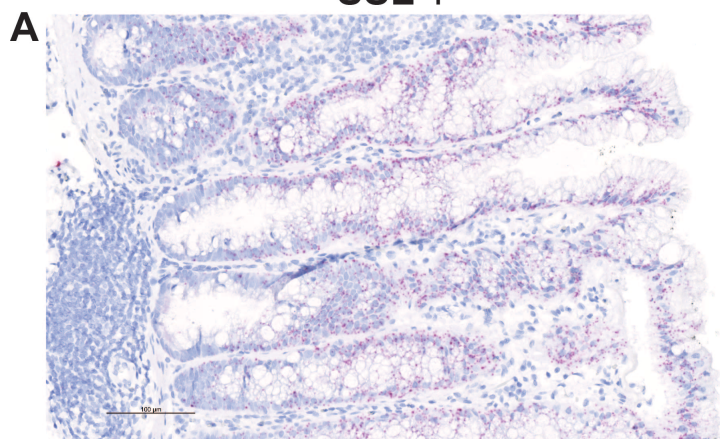

**SSL 1**

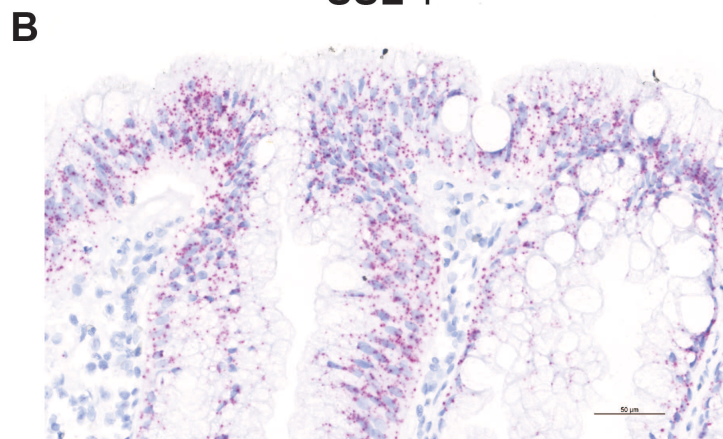

**cADN 1**

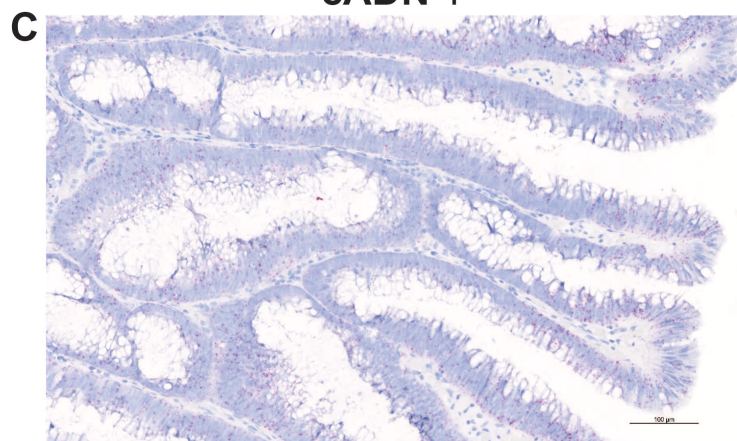

**cADN 1**

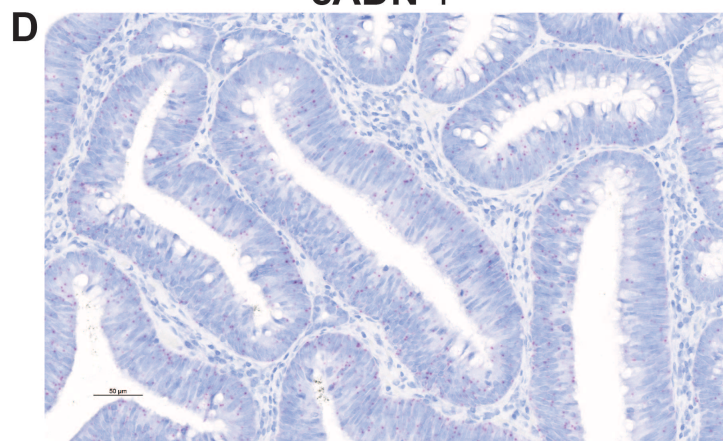

**cADN 2**

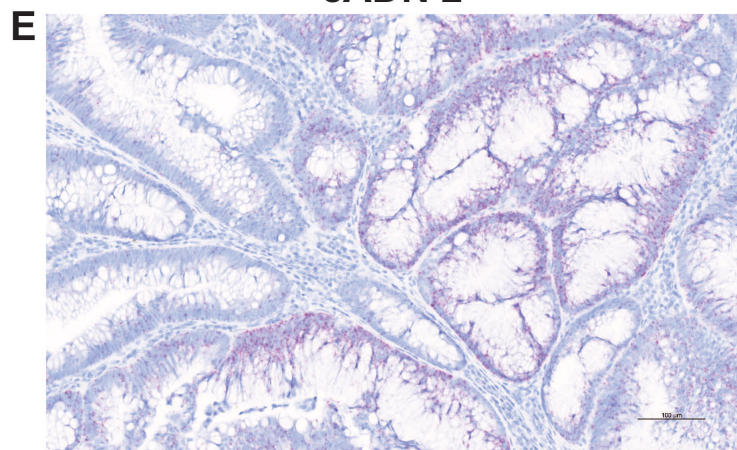

**TSA 2**

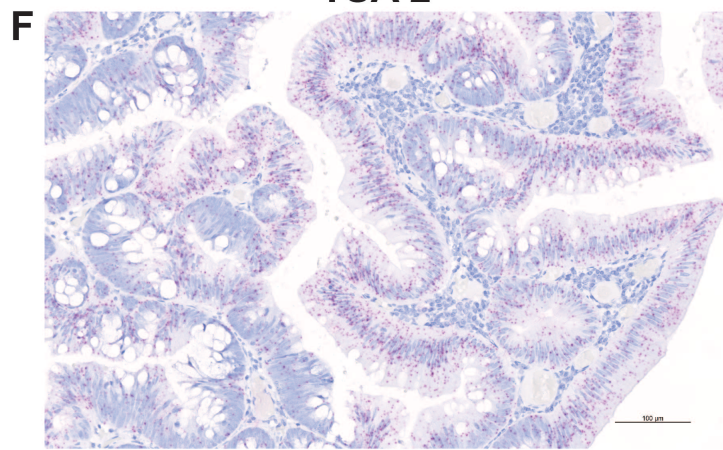

**TSA 3**

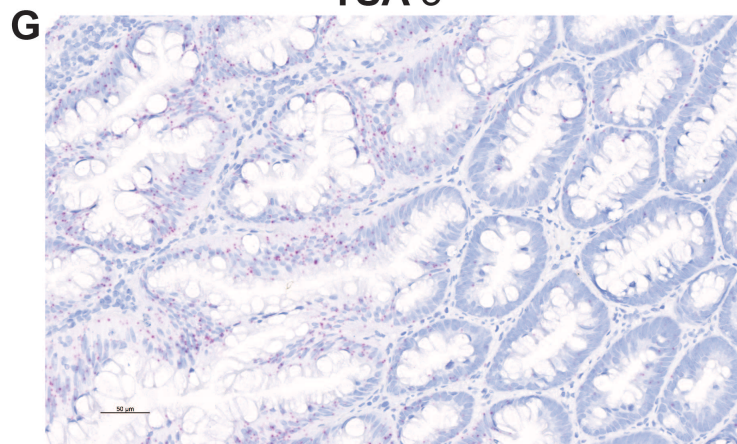

**Normal mucosa**

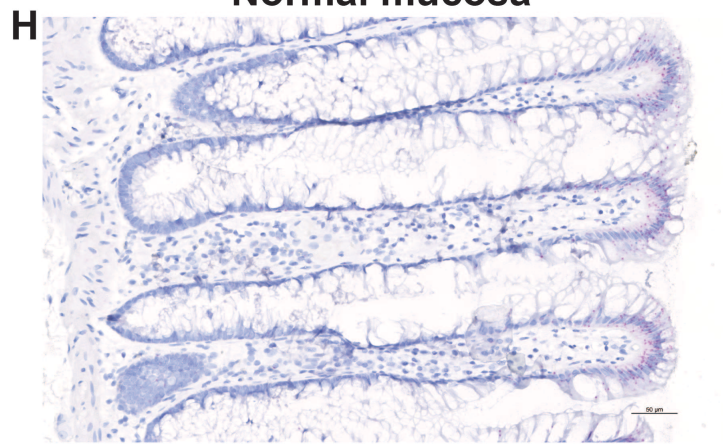

Supplement: Supplementary file 12 — Additional file 12: Supplementary Figure 12. In situ hybridization analysis of APOBEC1 in serrated precursor lesions, cADNs and normal colorectal mucosa. APOBEC1 was expressed in all the lesion types investigated, especially in the upper portions of glands, and also in the superficial epithelium of the normal mucosa (H) (Table 1). However, the expression was higher in SSLs, where it was absent only at the bases of serrated glands (A and B), and in HPs, where high expression was more confined to the surface of the lesions (Table 1). In TSAs and cADNs, moderate expression of APOBEC1 was seen in glands with more evident goblet-cell differentiation (C-G). [file 13000_2020_1064_MOESM12_ESM.pdf]

# *MUC5AC*

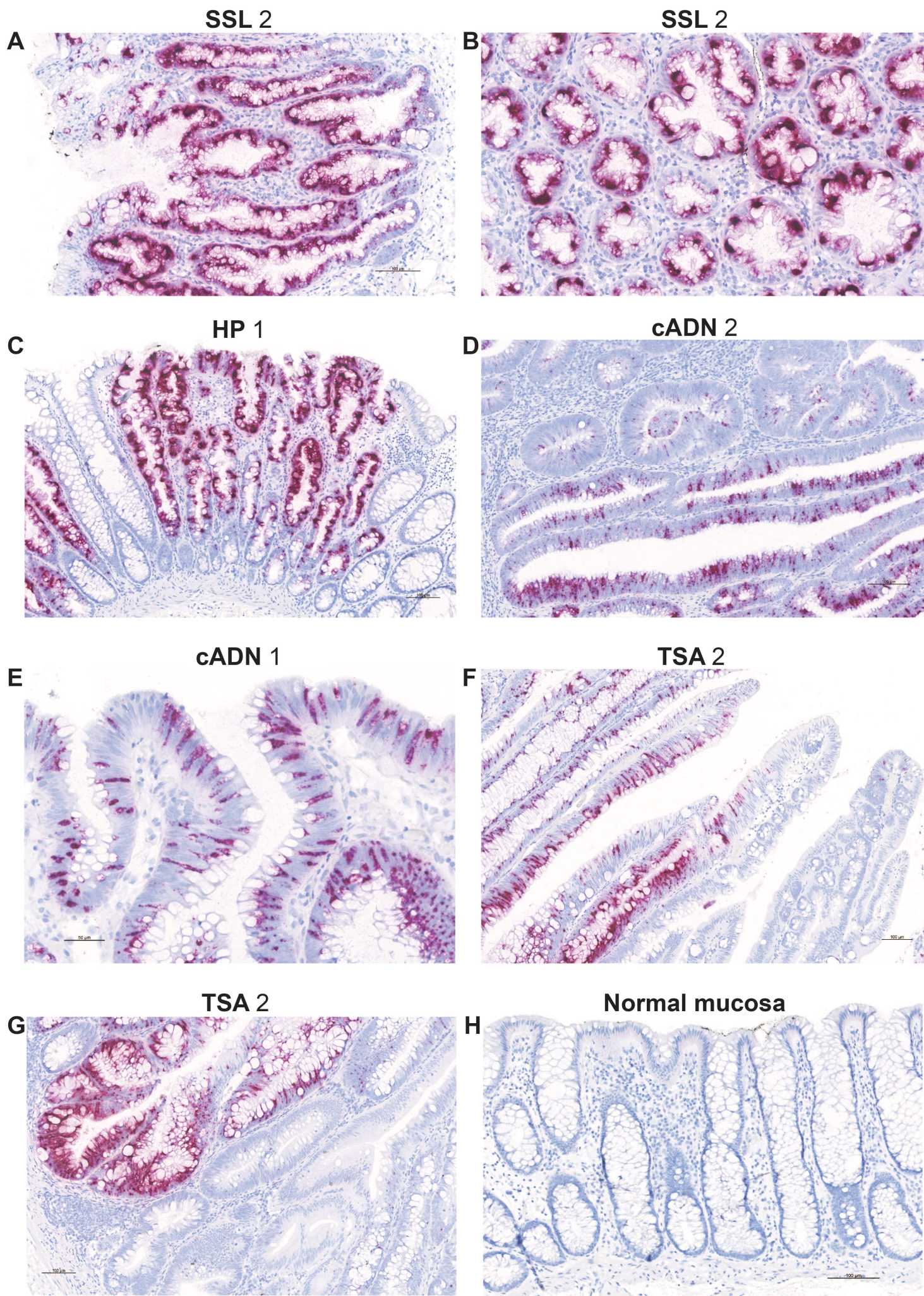

Supplement: Supplementary file 13 — Additional file 13: Supplementary Figure 13. In situ hybridization analysis of MUC5AC in serrated precursor lesions, cADNs and normal colorectal mucosa. MUC5AC expression, like that of APOBEC1, is not specific to serrated lesions. Very high levels were found in the mucous cells of all the lesions we tested, but not in those of the normal mucosa (H) (excluding rare positive cells in its surface; not shown). Very high MUC5AC expression was more extensive in SSLs and HPs (i.e., along the entire length of serrated crypts with lower levels only at their bases) (A-C), while patchier expression was observed in cADNs (D and E) and TSAs (F and G) (Table 1). MUC5AC therefore represents a marker of neoplastic goblet cells (e.g., panel E) but not of their normal mucosal counterparts (panel H). [file 13000_2020_1064_MOESM13_ESM.pdf]

# *NKD1*

**SSL 1**

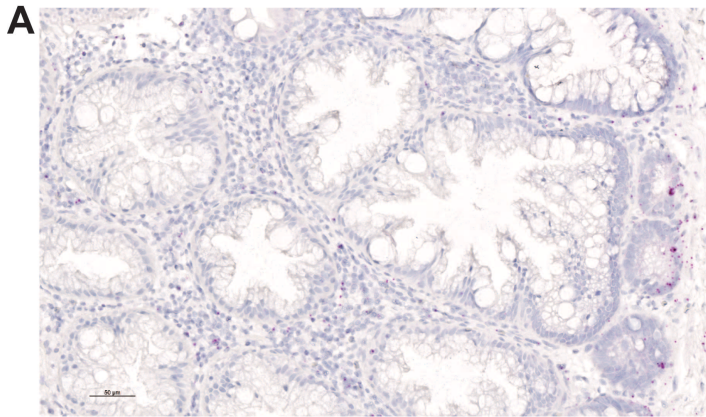

**HP 1**

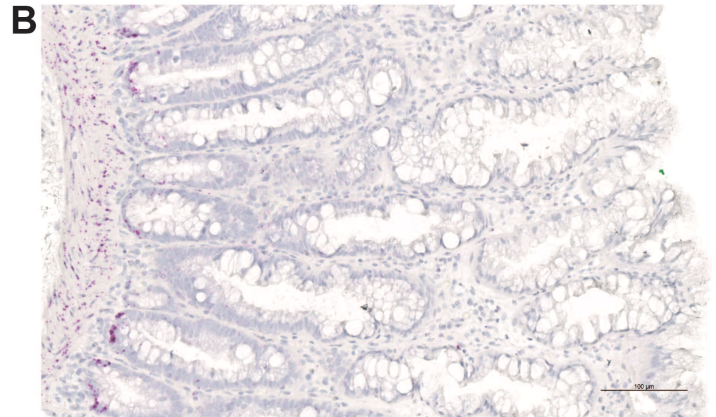

**cADN 2**

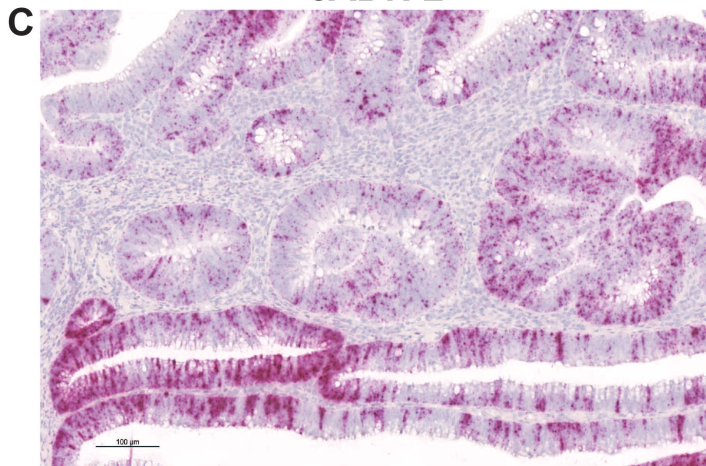

**cADN 2**

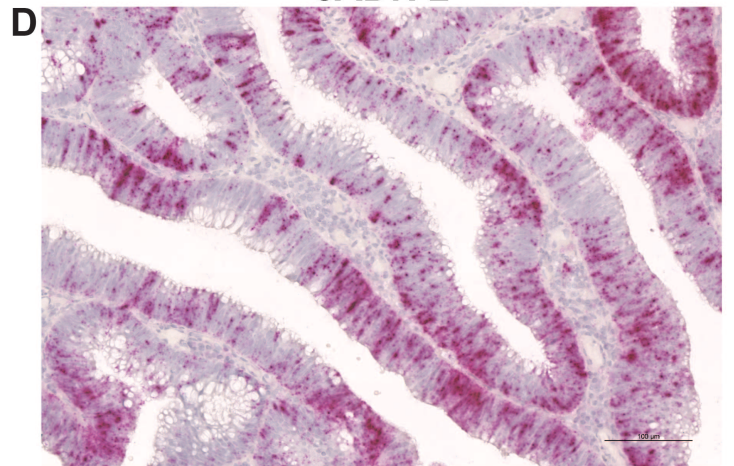

**TSA 2**

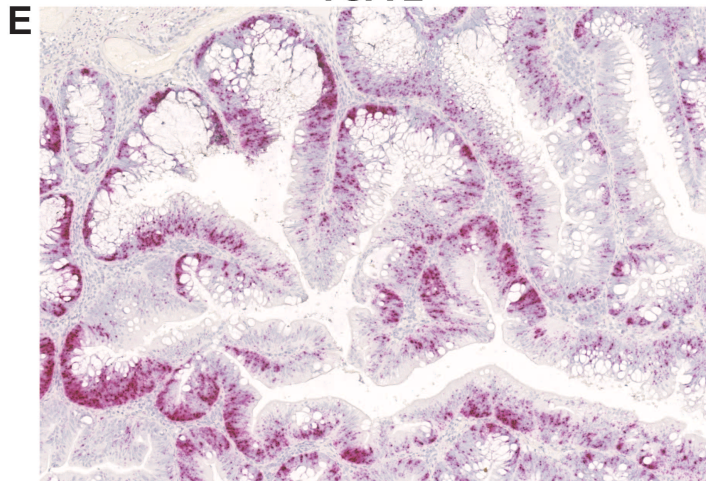

**TSA 2**

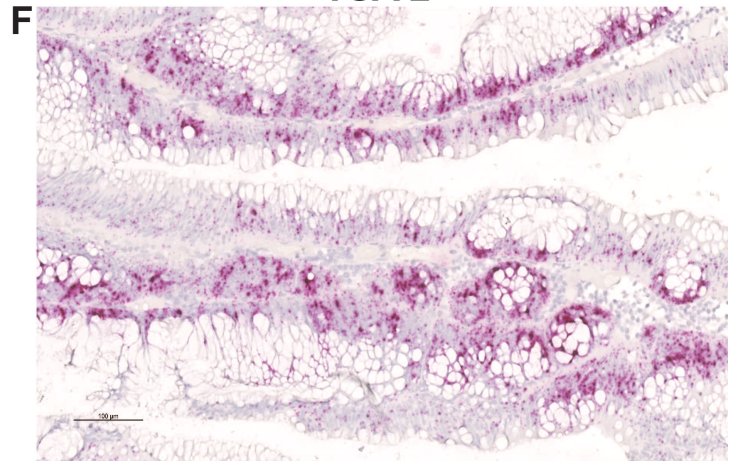

**TSA 3**

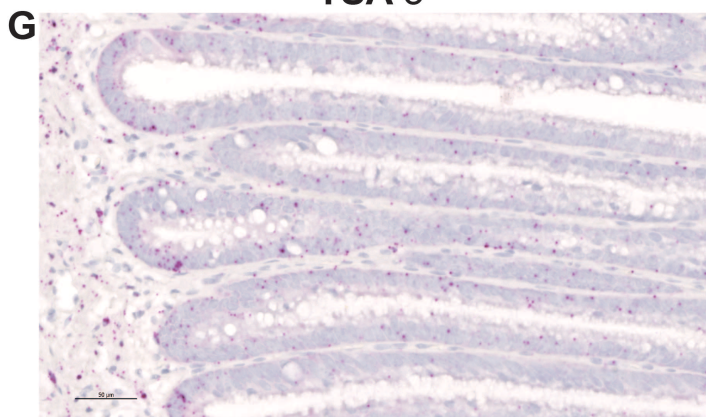

**TSA 3**

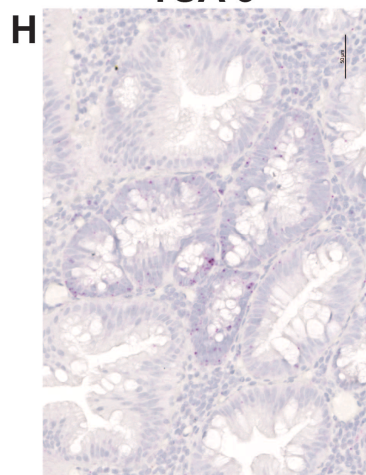

**Normal mucosa**

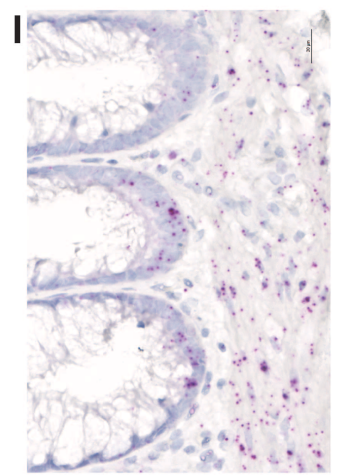

Supplement: Supplementary file 14 — Additional file 14: Supplementary Figure 14. In situ hybridization analysis of NKD1 in serrated precursor lesions, cADNs and normal colorectal mucosa. NKD1 is expressed only in a few cells within the stem-cell compartment at the bottom of serrated crypts in SSLs (A) and HPs (B) and at the bases of normal mucosal crypts (I). In cADNs, it is very highly and extensively expressed, with patchy variation of intensity (C and D), and similarly high expression was also observed in one of the TSAs (E and F). Its expression is much more limited in the other two TSAs (G and H, and Table 1). NKD1 is also expressed in some stromal cells and quite extensively in the muscularis mucosae (I). [file 13000_2020_1064_MOESM14_ESM.pdf]

# *HOXD13*

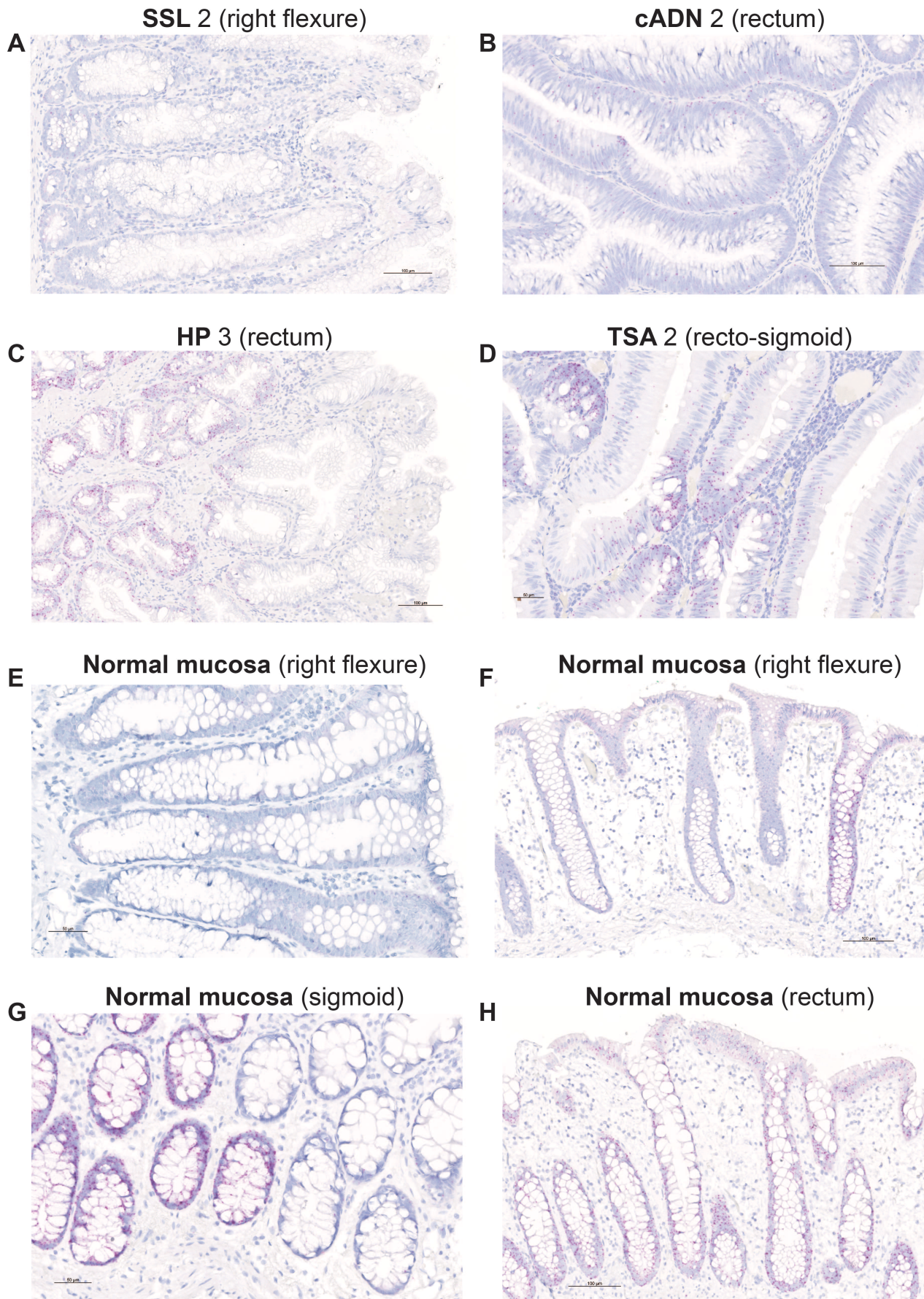

Supplement: Supplementary file 15 — Additional file 15: Supplementary Figure 15. In situ hybridization analysis of HOXD13 in serrated precursor lesions, cADNs and normal colorectal mucosa. In proximal-colon SSLs, HOXD13 is expressed only in a few cells at crypt bases (A) (Table 1). The two distal-colon HPs (especially HP 3, which arose in the rectum) displayed moderate expression at the crypt bases (C). Patchy, low-to-moderate HOXD13 expression was also present in cADNs and TSAs (B and D), and only a few sporadic positive crypts were noted in the normal mucosa of the proximal colon (E and F). In contrast, it was expressed in the normal mucosa of the distal colon and rectum at moderate levels, although patches of negative crypts were also seen (G and H). [file 13000_2020_1064_MOESM15_ESM.pdf]

*HOXB13*

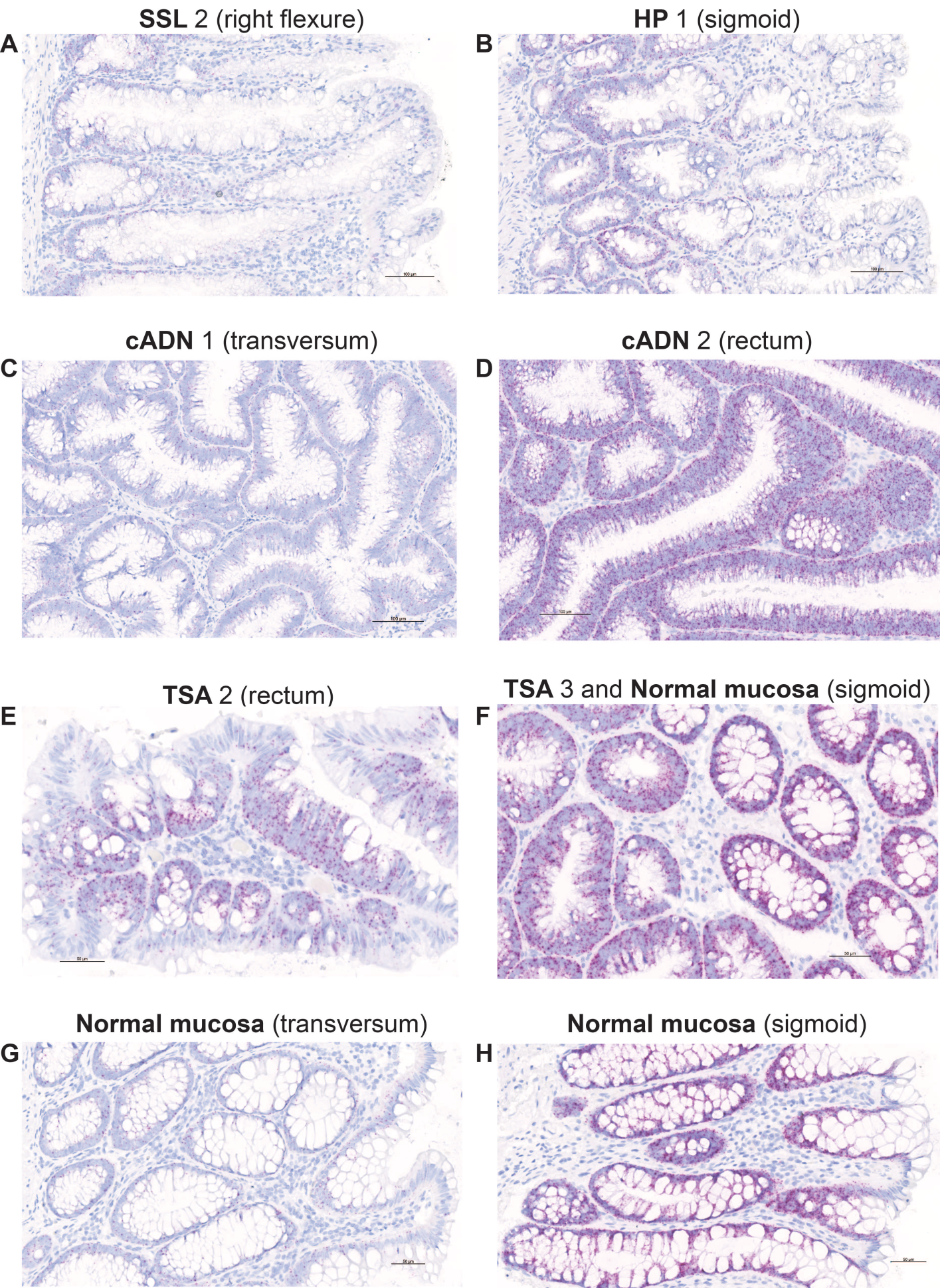

Supplement: Supplementary file 16 — Additional file 16: Supplementary Figure 16. In situ hybridization analysis of HOXB13 in serrated precursor lesions, cADNs and normal colorectal mucosa. HOXB13 was generally more highly expressed than HOXD13 in colorectal tissues (Table 1; see also Supplementary Figures 2 and 3), but, similarly to HOXD13, it is a typical marker of the normal mucosal of the distal colon and rectum (H). In the normal mucosa of the proximal colon, expression was low or absent (G). Moderate-to-high HOXB13 expression was found in SSLs (A), HPs (B), TSAs (E and F), and cADNs (C and D), but in all four lesion types, more abundant expression was found in tumors taken from the distal colon and rectum (Table 1). [file 13000_2020_1064_MOESM16_ESM.pdf]

**FAM3B**

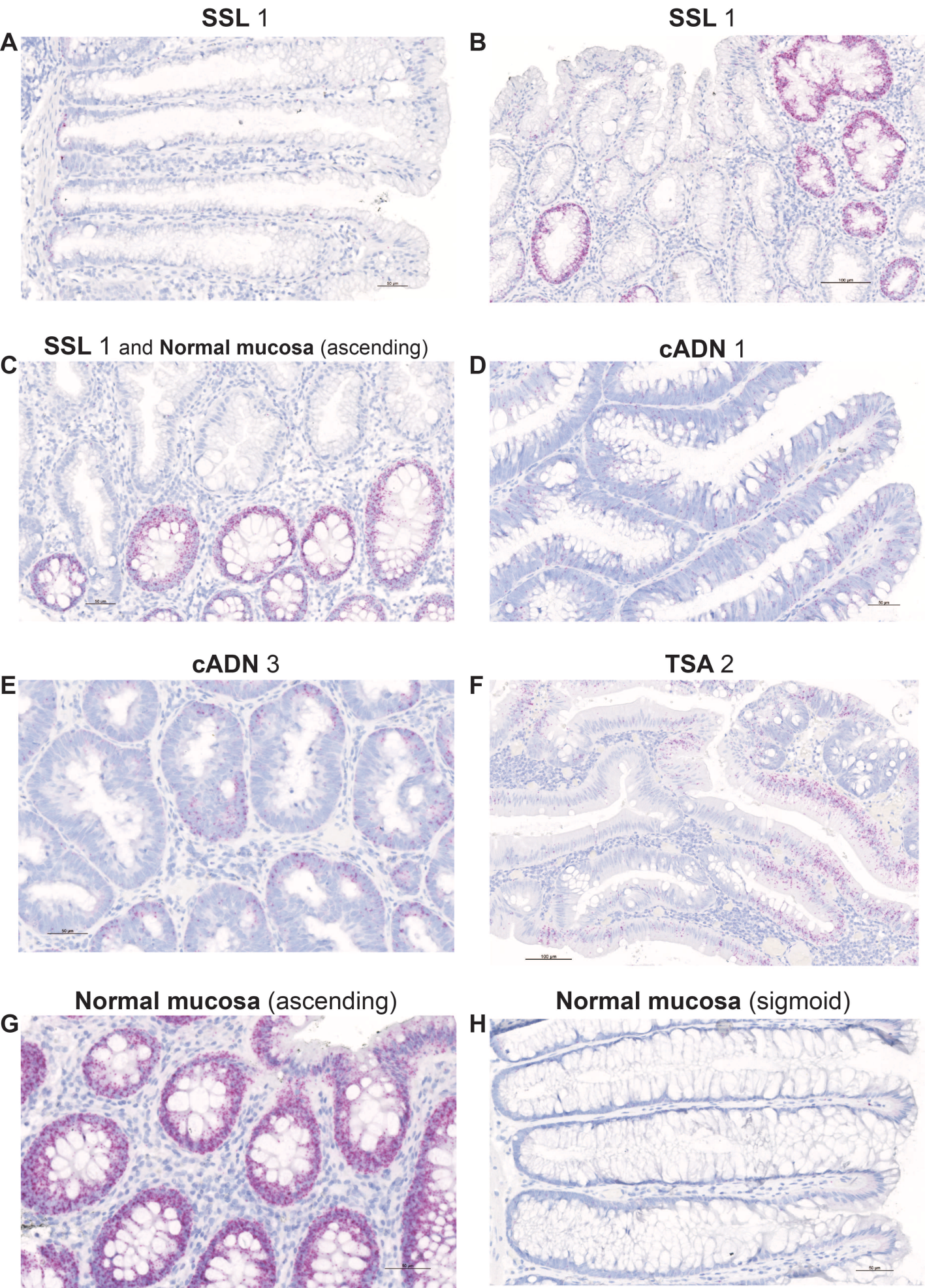

Supplement: Supplementary file 17 — Additional file 17: Supplementary Figure 17. In situ hybridization analysis of FAM3B in serrated precursor lesions, cADNs, and normal colorectal mucosa. In contrast to HOXD13 and HOXB13 of the two previous supplementary figures, FAM3B is highly expressed in the normal epithelium of the proximal colon (C and G) but unexpressed in the distal colon (H) (Table 1; see also Supplementary Figures 2 and 3). Moderate and localized (patchy or superficial) staining was seen in all tumors regardless of type and colorectal segment of origin (A, B, D, E, and F), although higher levels were often found in lesions from the proximal colon. [file 13000_2020_1064_MOESM17_ESM.pdf]

H&E image  
of cADN 1

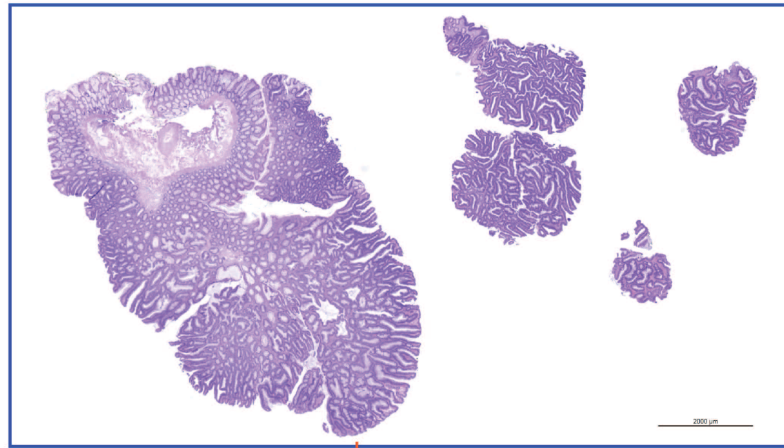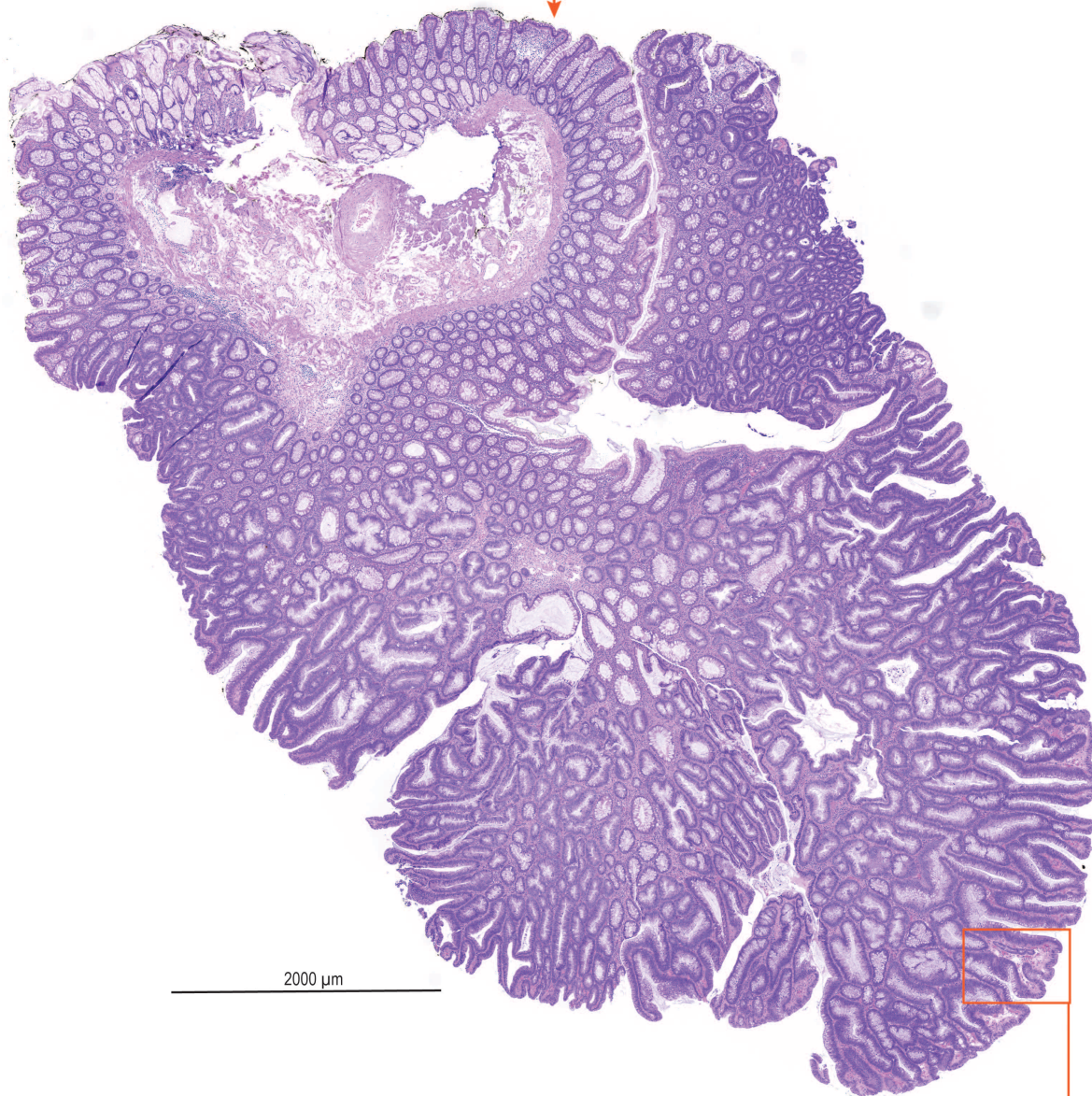

panel F of Figure 2

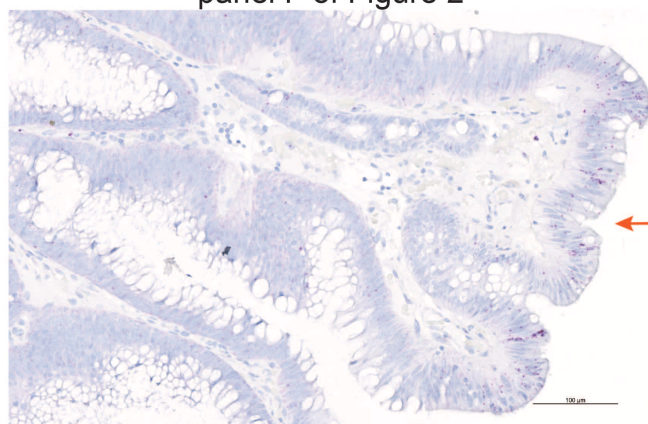

same region  
as that shown  
in Figure 2F

Supplement: Supplementary file 18 — Additional file 18: Supplementary Figures 18–29. H&E-stained sections of each of the 12 lesions investigated in this study. [file 13000_2020_1064_MOESM18_ESM.zip › Supplementary Figure 18.pdf]

H&E image  
of cADN 2

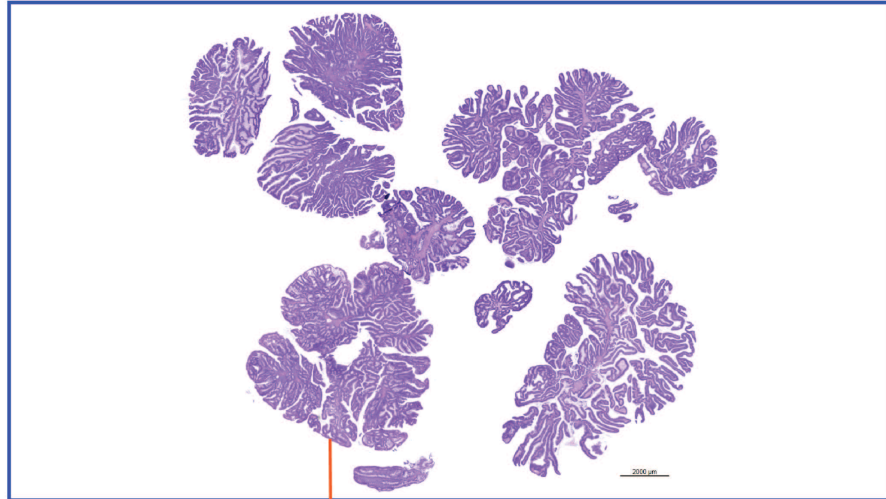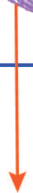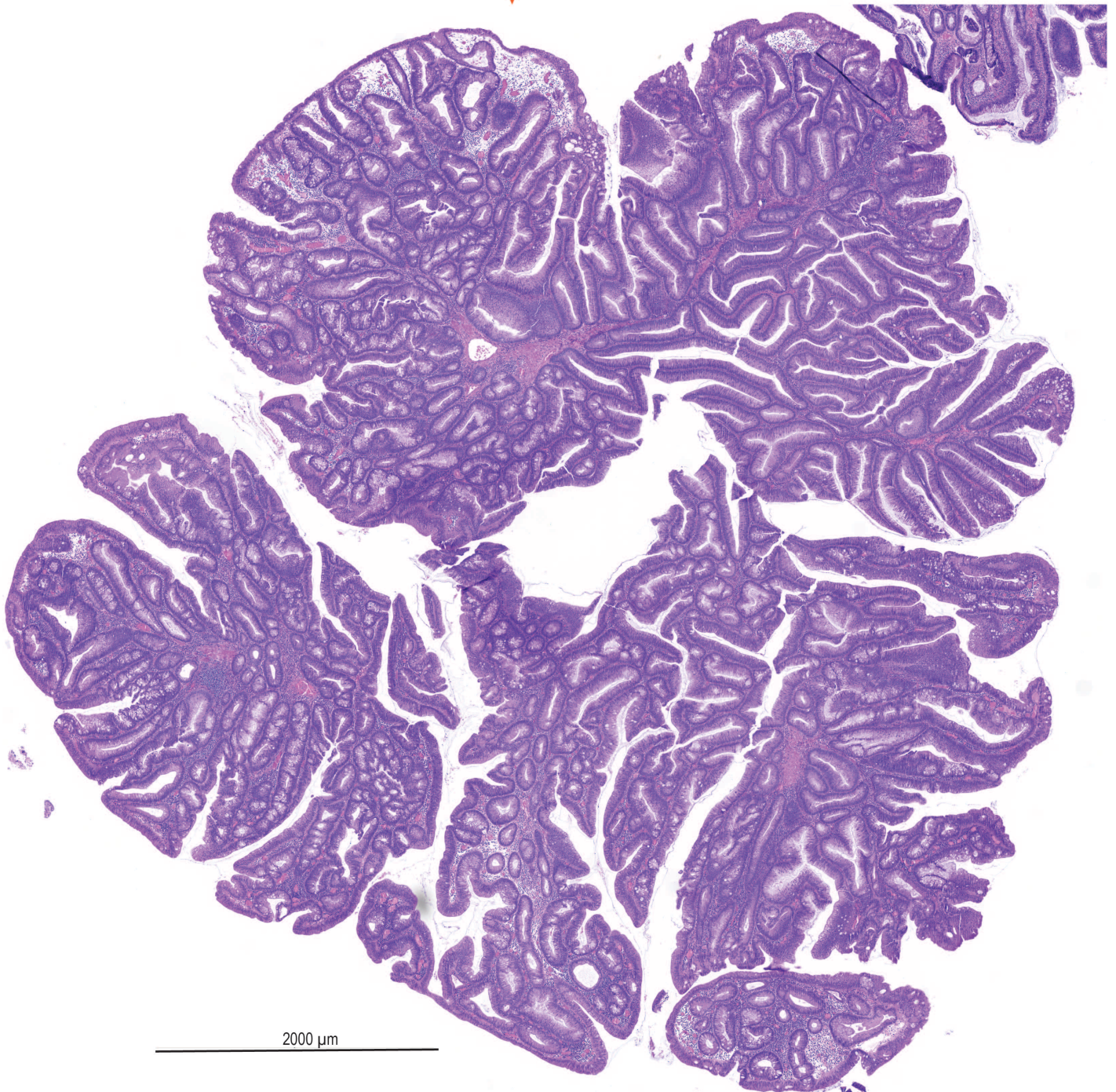

Supplement: Supplementary file 18 — Additional file 18: Supplementary Figures 18–29. H&E-stained sections of each of the 12 lesions investigated in this study. [file 13000_2020_1064_MOESM18_ESM.zip › Supplementary Figure 19.pdf]

H&E image  
of cADN 3

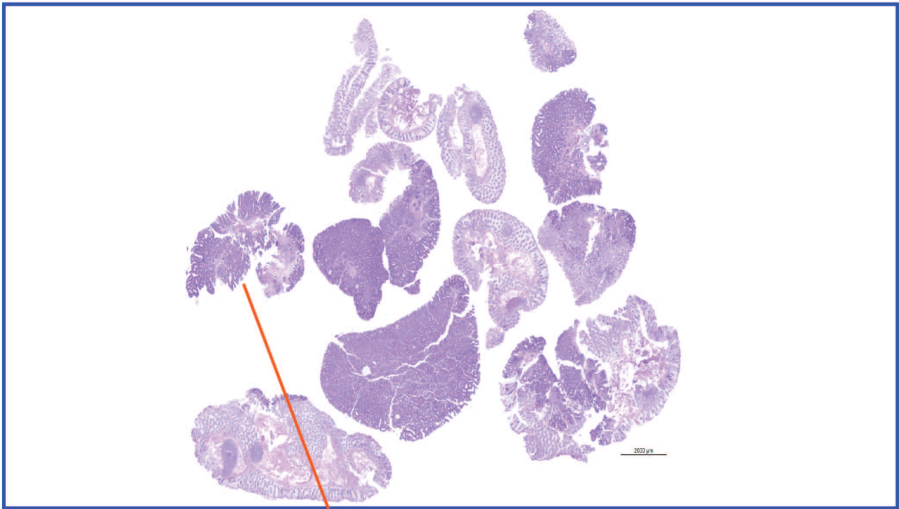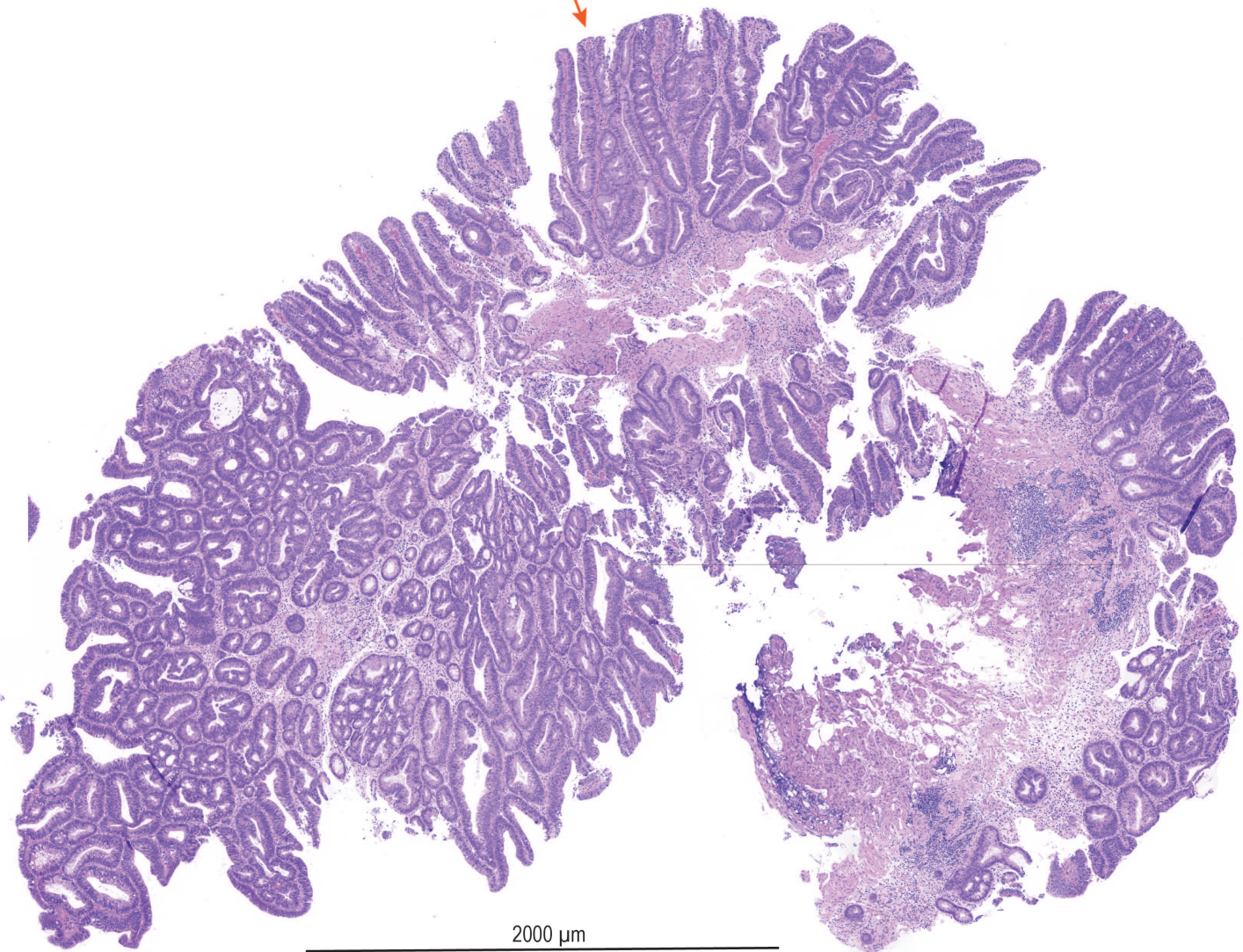

Supplement: Supplementary file 18 — Additional file 18: Supplementary Figures 18–29. H&E-stained sections of each of the 12 lesions investigated in this study. [file 13000_2020_1064_MOESM18_ESM.zip › Supplementary Figure 20.pdf]

H&E image  
of SSL 1

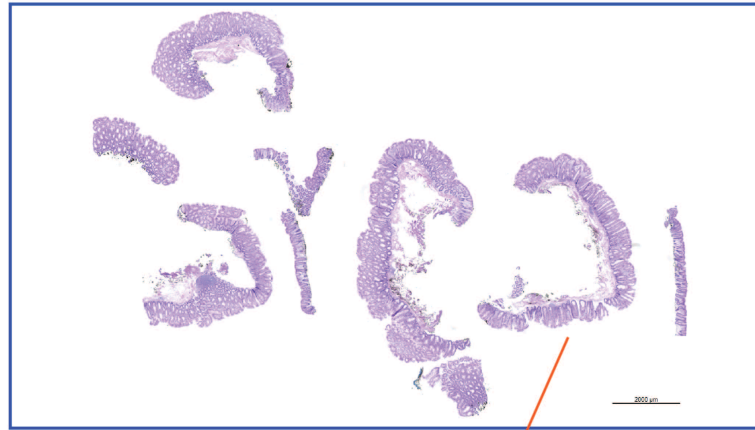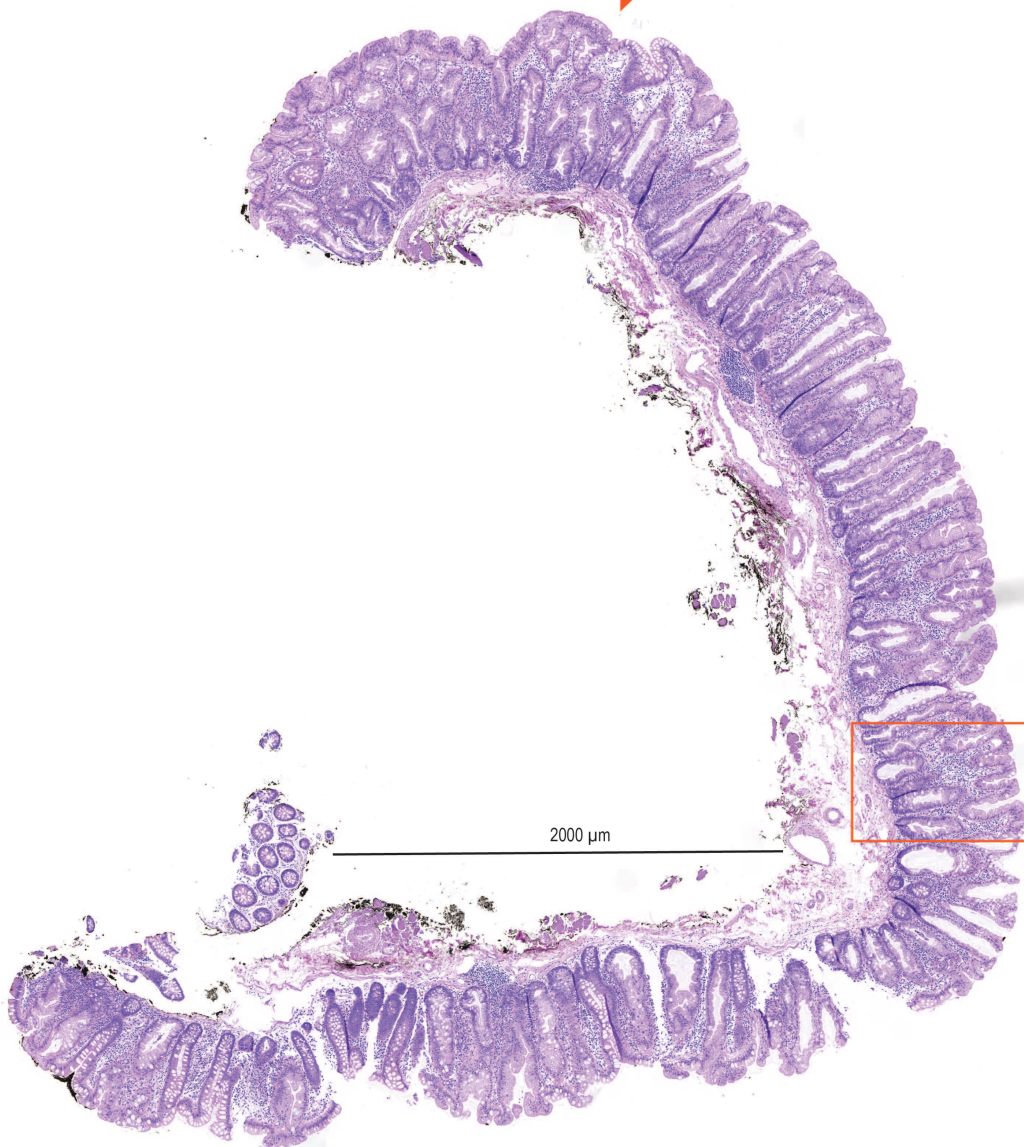

panel C of Figure 2

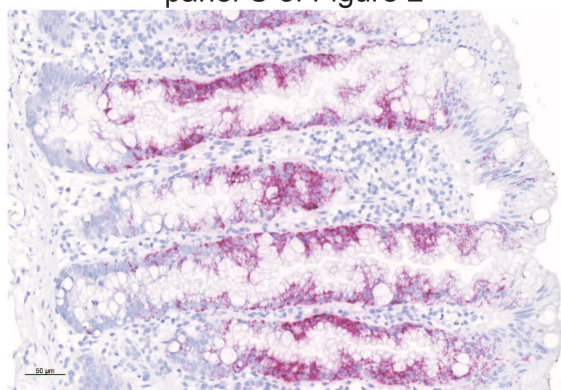

same region  
as that shown  
in Figure 2C

Supplement: Supplementary file 18 — Additional file 18: Supplementary Figures 18–29. H&E-stained sections of each of the 12 lesions investigated in this study. [file 13000_2020_1064_MOESM18_ESM.zip › Supplementary Figure 21.pdf]

H&E image  
of SSL 2

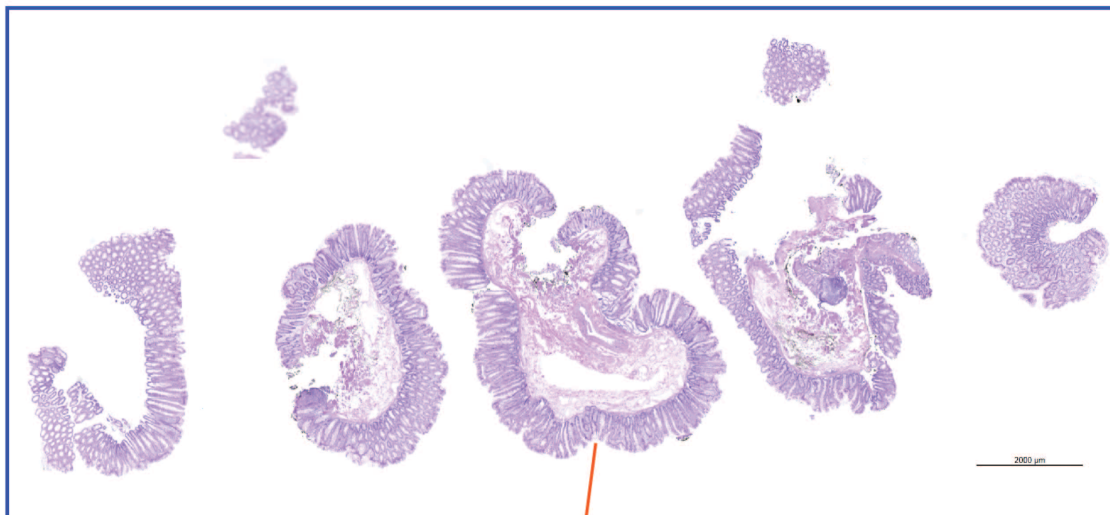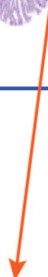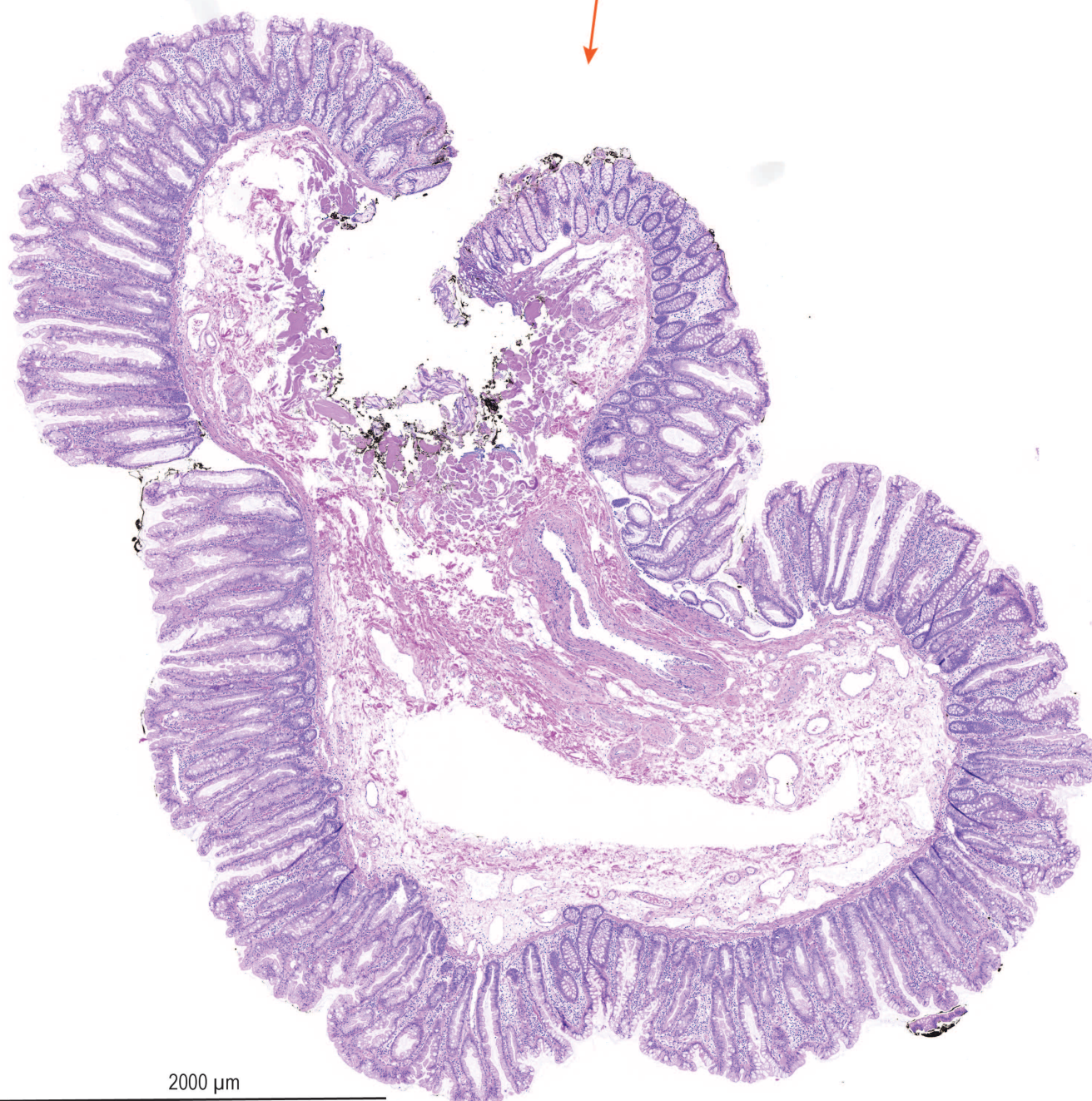

Supplement: Supplementary file 18 — Additional file 18: Supplementary Figures 18–29. H&E-stained sections of each of the 12 lesions investigated in this study. [file 13000_2020_1064_MOESM18_ESM.zip › Supplementary Figure 22.pdf]

H&E image  
of SSL 3

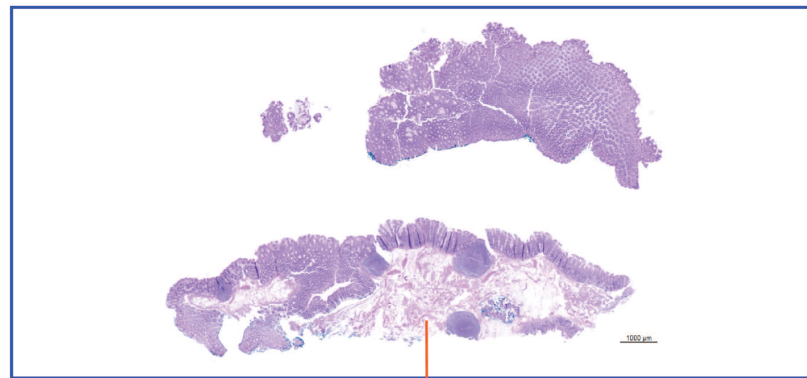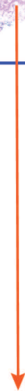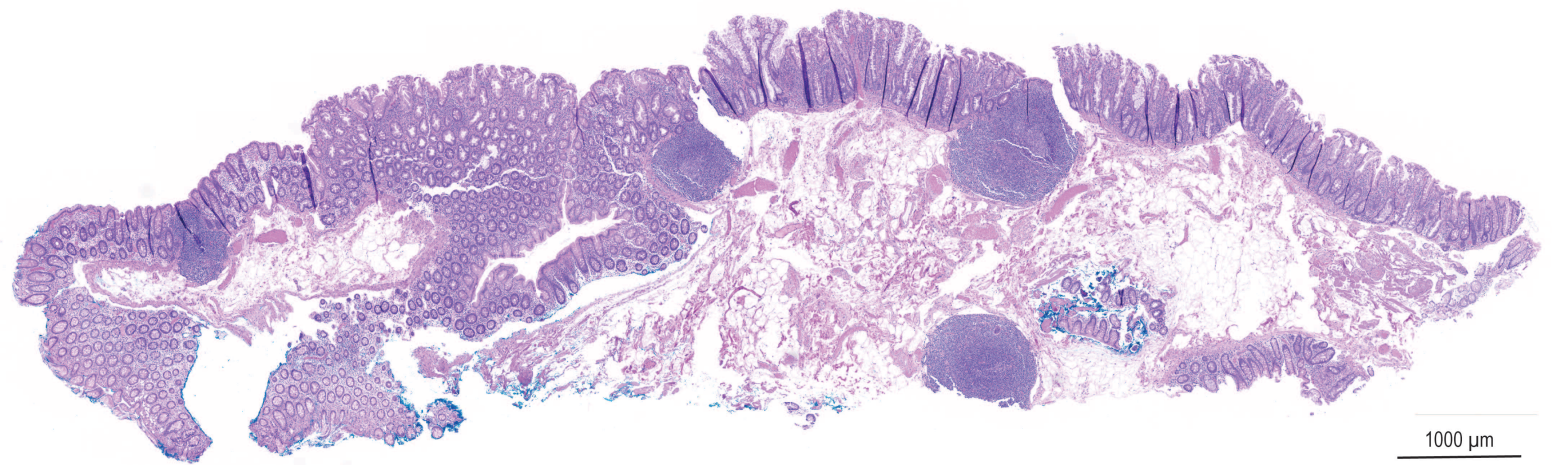

Supplement: Supplementary file 18 — Additional file 18: Supplementary Figures 18–29. H&E-stained sections of each of the 12 lesions investigated in this study. [file 13000_2020_1064_MOESM18_ESM.zip › Supplementary Figure 23.pdf]

H&E image  
of HP 1

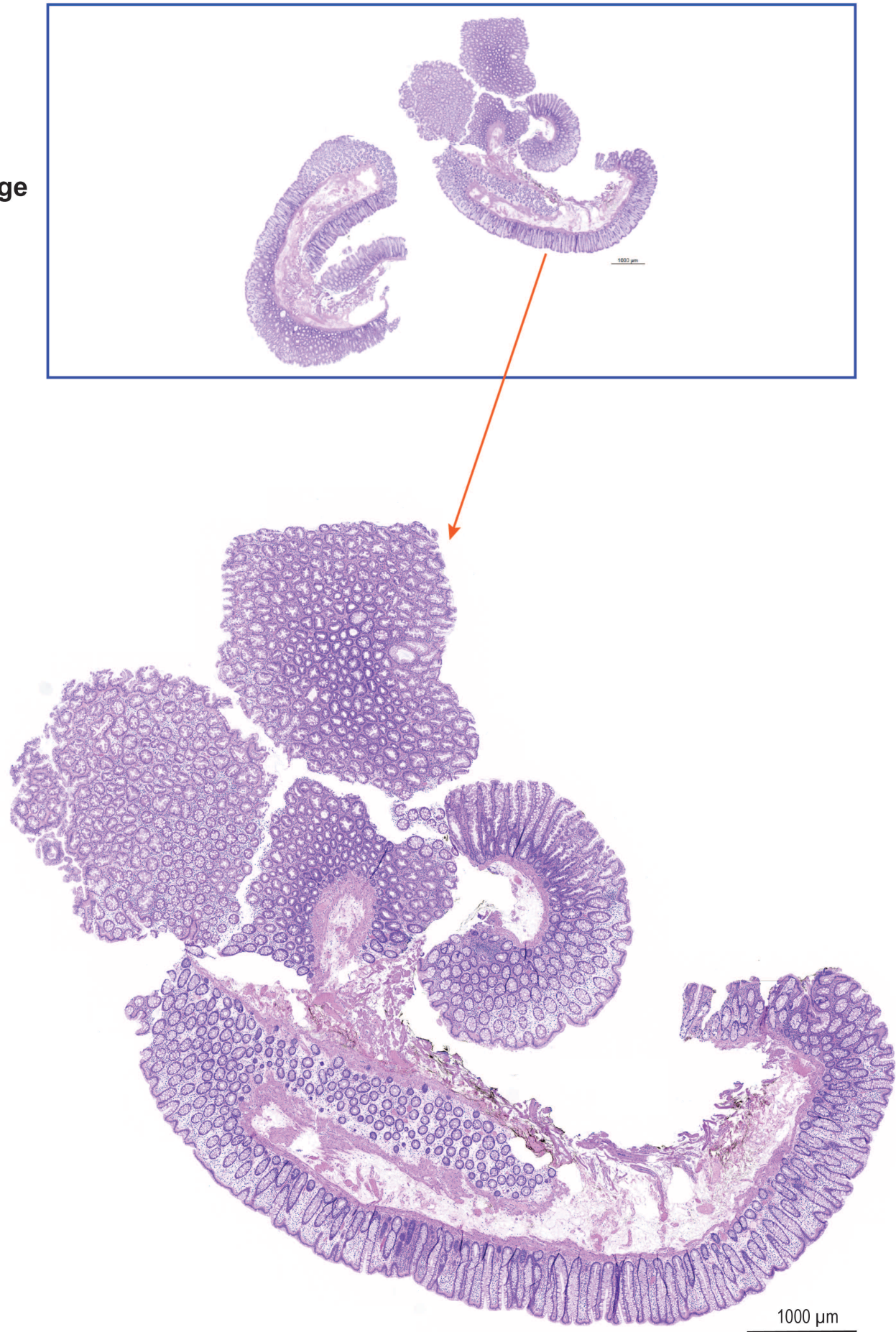

Supplement: Supplementary file 18 — Additional file 18: Supplementary Figures 18–29. H&E-stained sections of each of the 12 lesions investigated in this study. [file 13000_2020_1064_MOESM18_ESM.zip › Supplementary Figure 24.pdf]

H&E image  
of HP 2

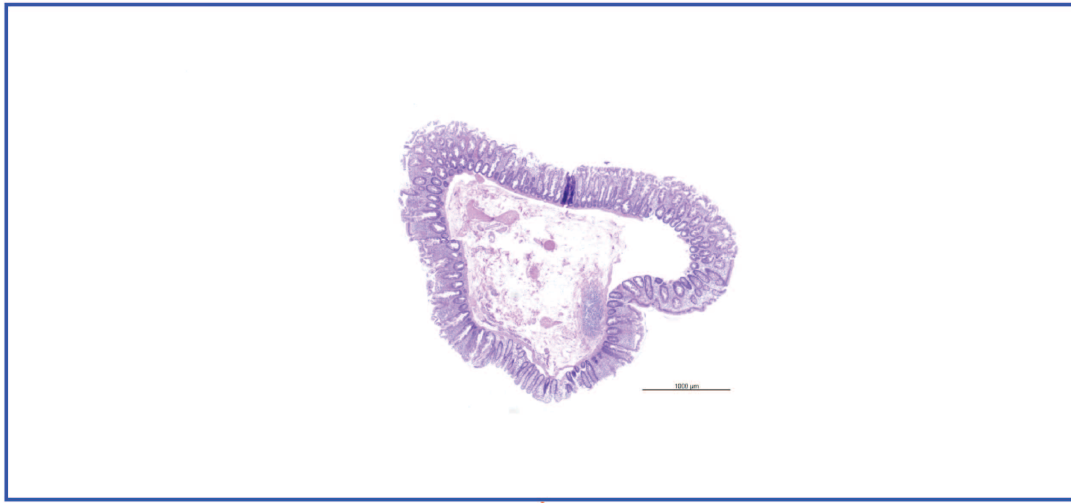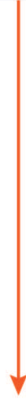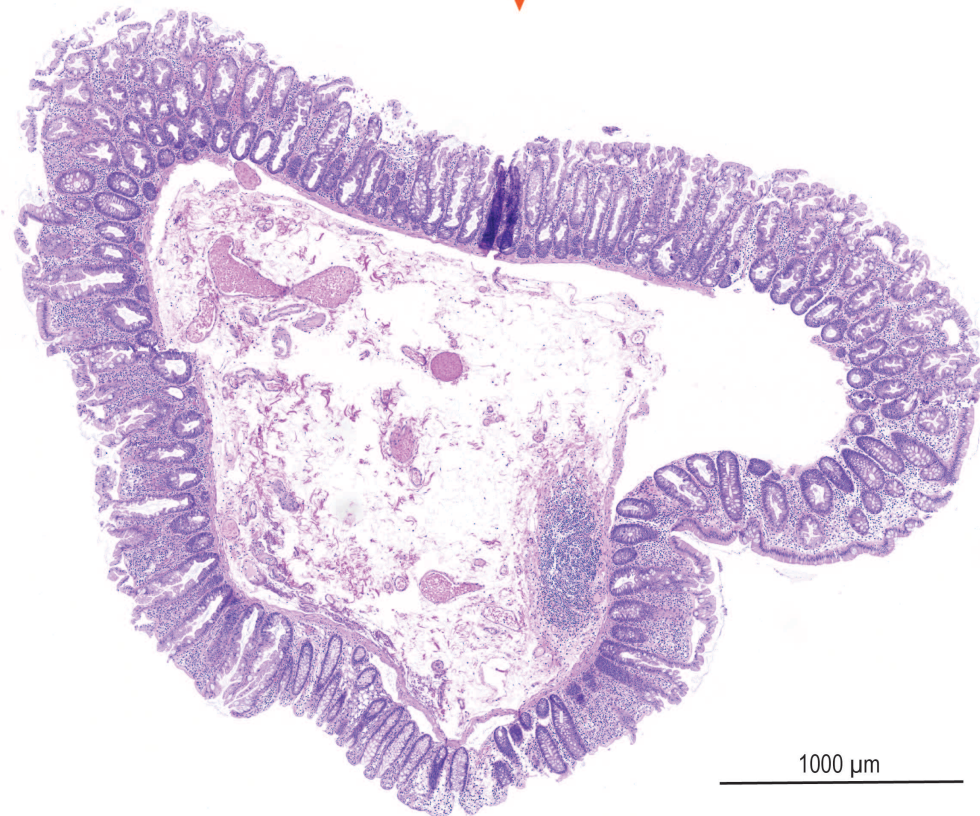

Supplement: Supplementary file 18 — Additional file 18: Supplementary Figures 18–29. H&E-stained sections of each of the 12 lesions investigated in this study. [file 13000_2020_1064_MOESM18_ESM.zip › Supplementary Figure 25.pdf]

H&E image  
of HP 3

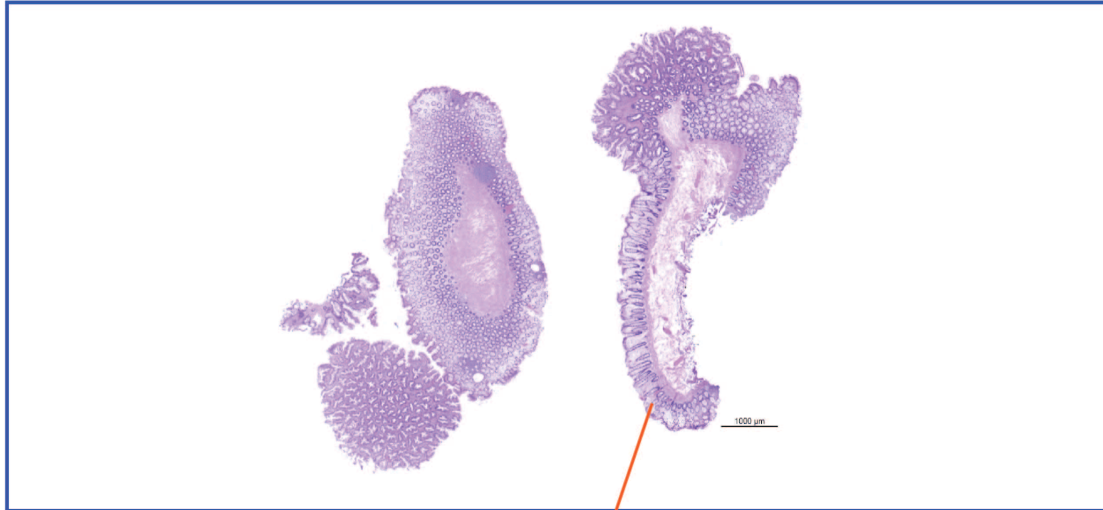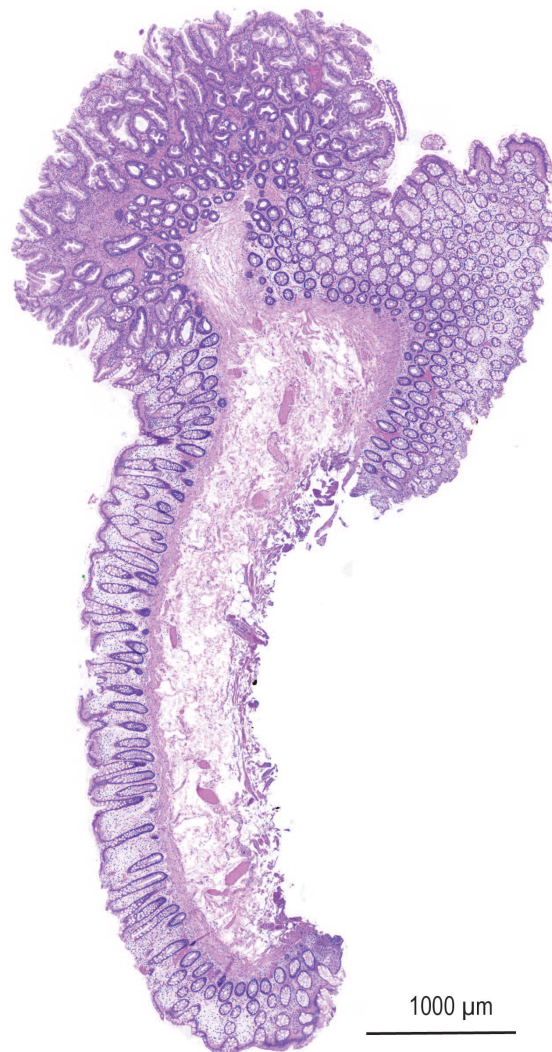

Supplement: Supplementary file 18 — Additional file 18: Supplementary Figures 18–29. H&E-stained sections of each of the 12 lesions investigated in this study. [file 13000_2020_1064_MOESM18_ESM.zip › Supplementary Figure 26.pdf]

H&E image  
of TSA 1

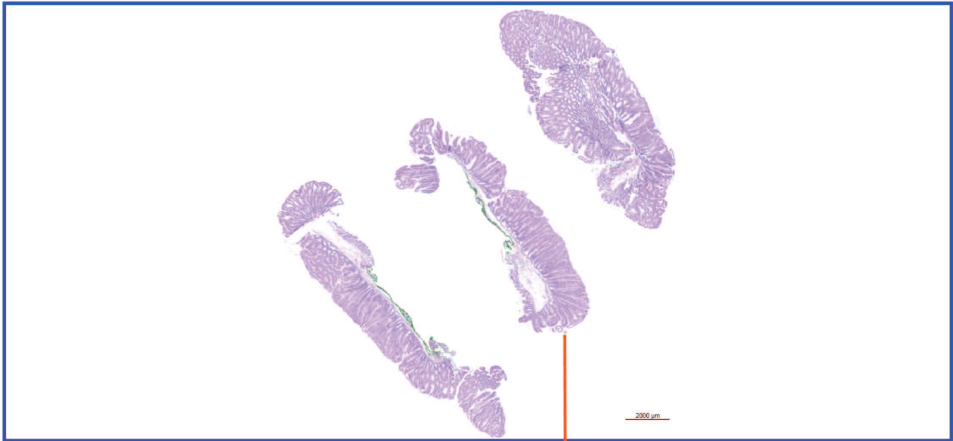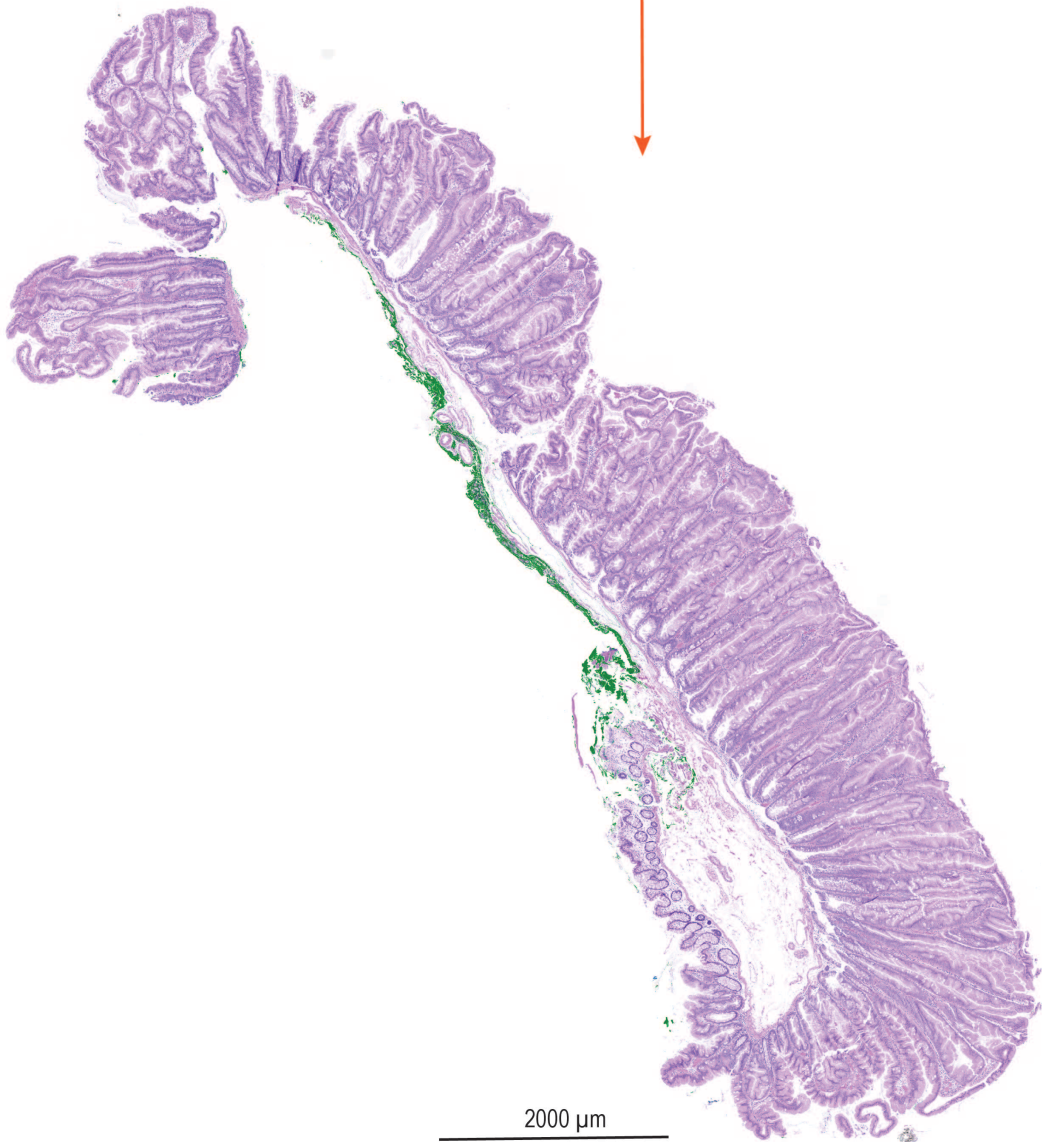

Supplement: Supplementary file 18 — Additional file 18: Supplementary Figures 18–29. H&E-stained sections of each of the 12 lesions investigated in this study. [file 13000_2020_1064_MOESM18_ESM.zip › Supplementary Figure 27.pdf]

H&E image  
of TSA 2

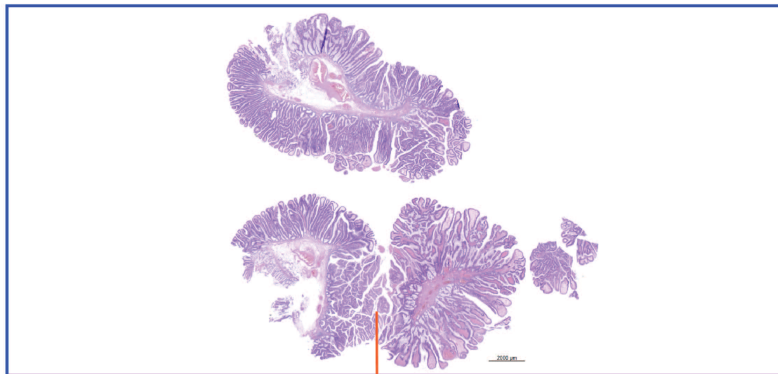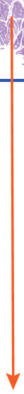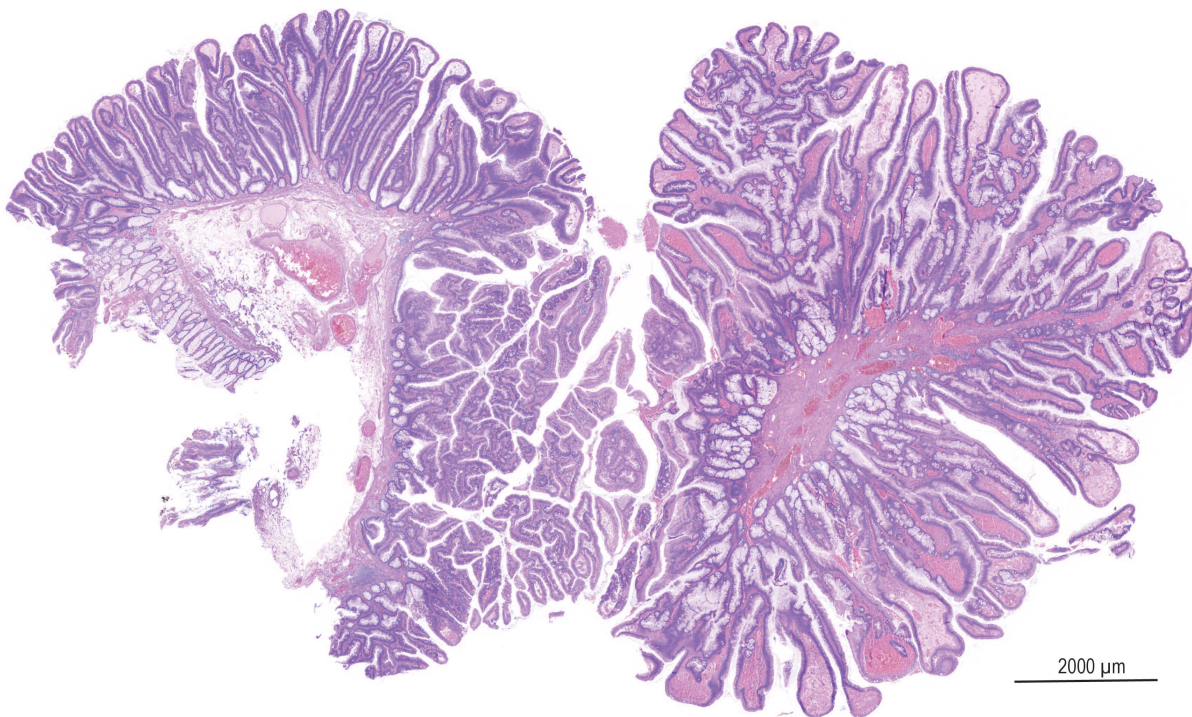

Supplement: Supplementary file 18 — Additional file 18: Supplementary Figures 18–29. H&E-stained sections of each of the 12 lesions investigated in this study. [file 13000_2020_1064_MOESM18_ESM.zip › Supplementary Figure 28.pdf]

H&E image  
of TSA 3

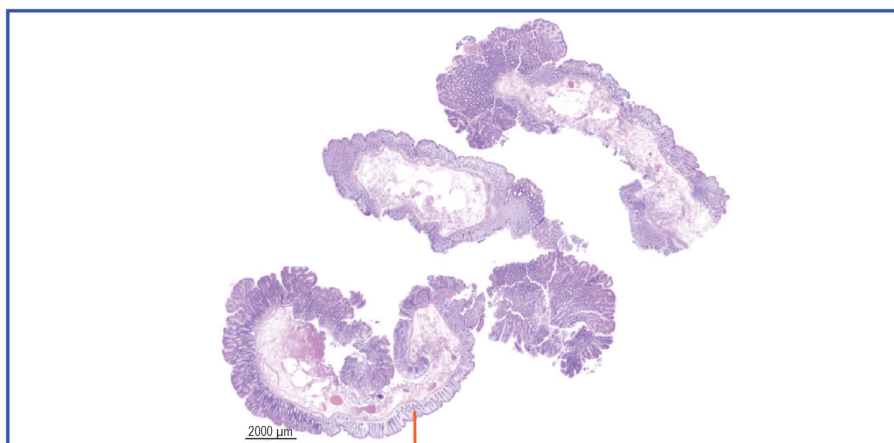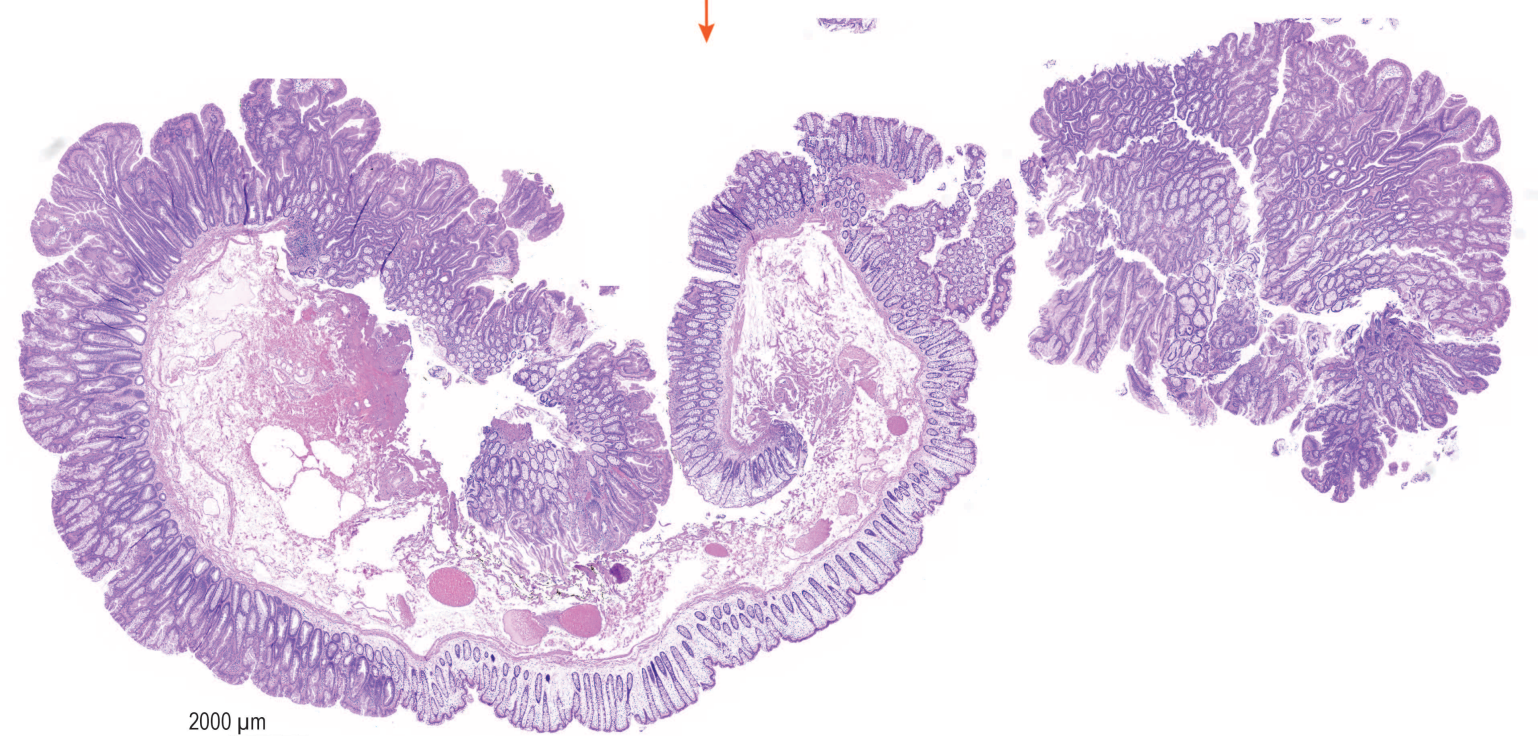

Supplement: Supplementary file 18 — Additional file 18: Supplementary Figures 18–29. H&E-stained sections of each of the 12 lesions investigated in this study. [file 13000_2020_1064_MOESM18_ESM.zip › Supplementary Figure 29.pdf]
