# Supplementary material for: An “expressionistic” look at serrated precancerous colorectal lesions
Source: Diagn Pathol. 2021 Jan 10;16:4. doi: 10.1186/s13000-020-01064-1 (PMC7797135; doi:10.1186/s13000-020-01064-1)
Supplement: Supplementary file 2 — Additional file 2: Supplementary Figure 2. (Panels A through O). RNA-sequencing-based expression profiles of the targets included in this study based on data published by Parker et al. (reference [21]). Integrative Genomics Viewer snapshots are shown for the serrated lesions and cADNs investigated by Parker et al. All lesions assessed in this study were from the proximal colon (details in reference [21]). Seventeen SSLs are compared with 15 cADNs, and each track in the snapshot shows the level of a given RNA (i.e., peaks across exons proportional to the number of sequencing reads) in the lesion (SSLs: red track: cADNs: blue track). Below each of these tracks is a track showing the expression level of the same RNA in a patient-matched sample of normal mucosa from the proximal colon (i.e., cecum, ascending, hepatic flexure or transversum) harboring the precancerous lesion (pink track: normal mucosa of an SSL carrier; light blue track: normal mucosa of a cADN carrier). [file 13000_2020_1064_MOESM2_ESM.pdf]

A ***VSIG1*** **mRNA expression (chromosome X)**

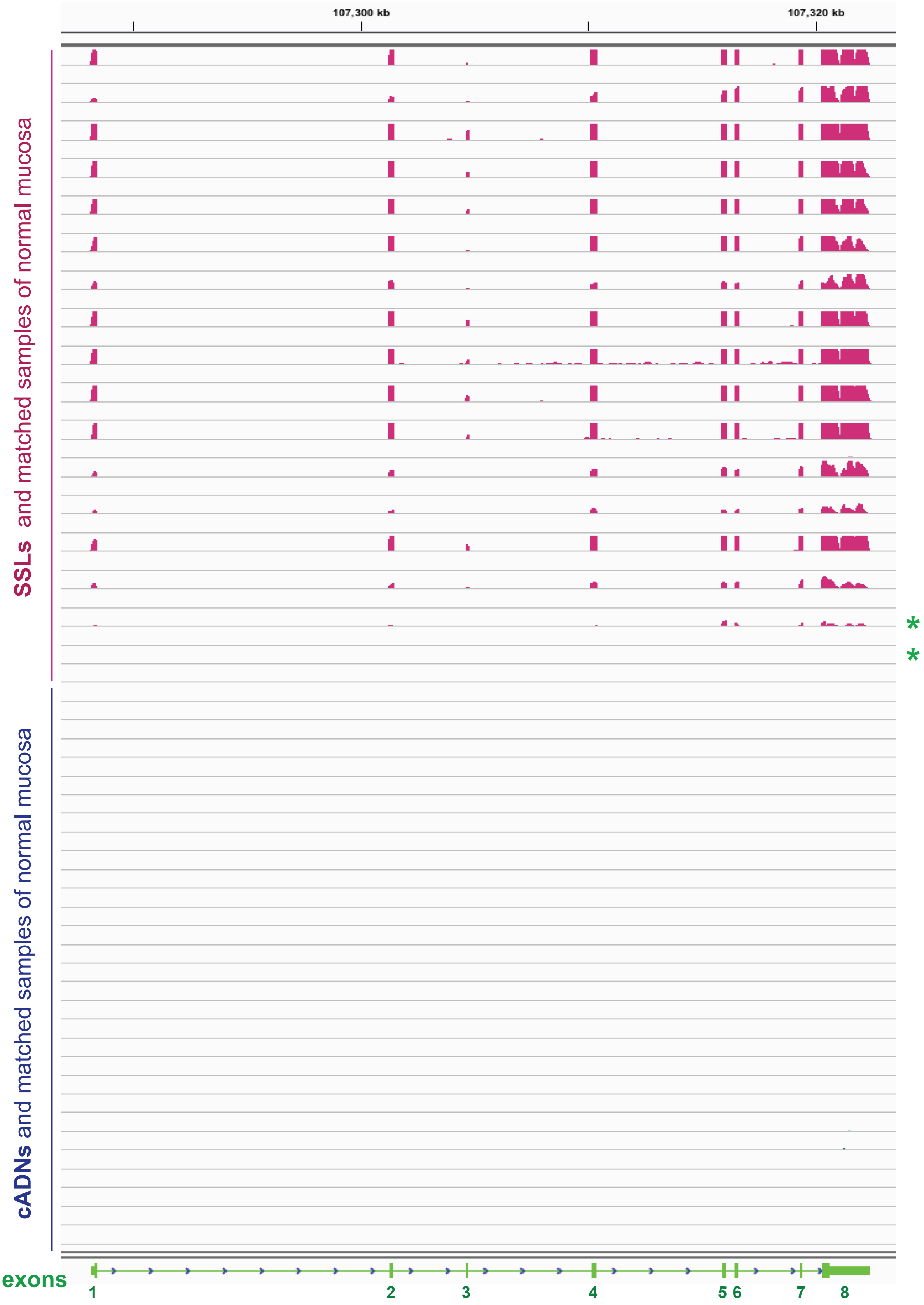

*The 2 lesions showing histologic features of both SSLs and cADNs (samples S6 and S16 of Table 1, Parker H. et al. 2018)

B ***ANXA10*** **mRNA expression (chromosome 4)**

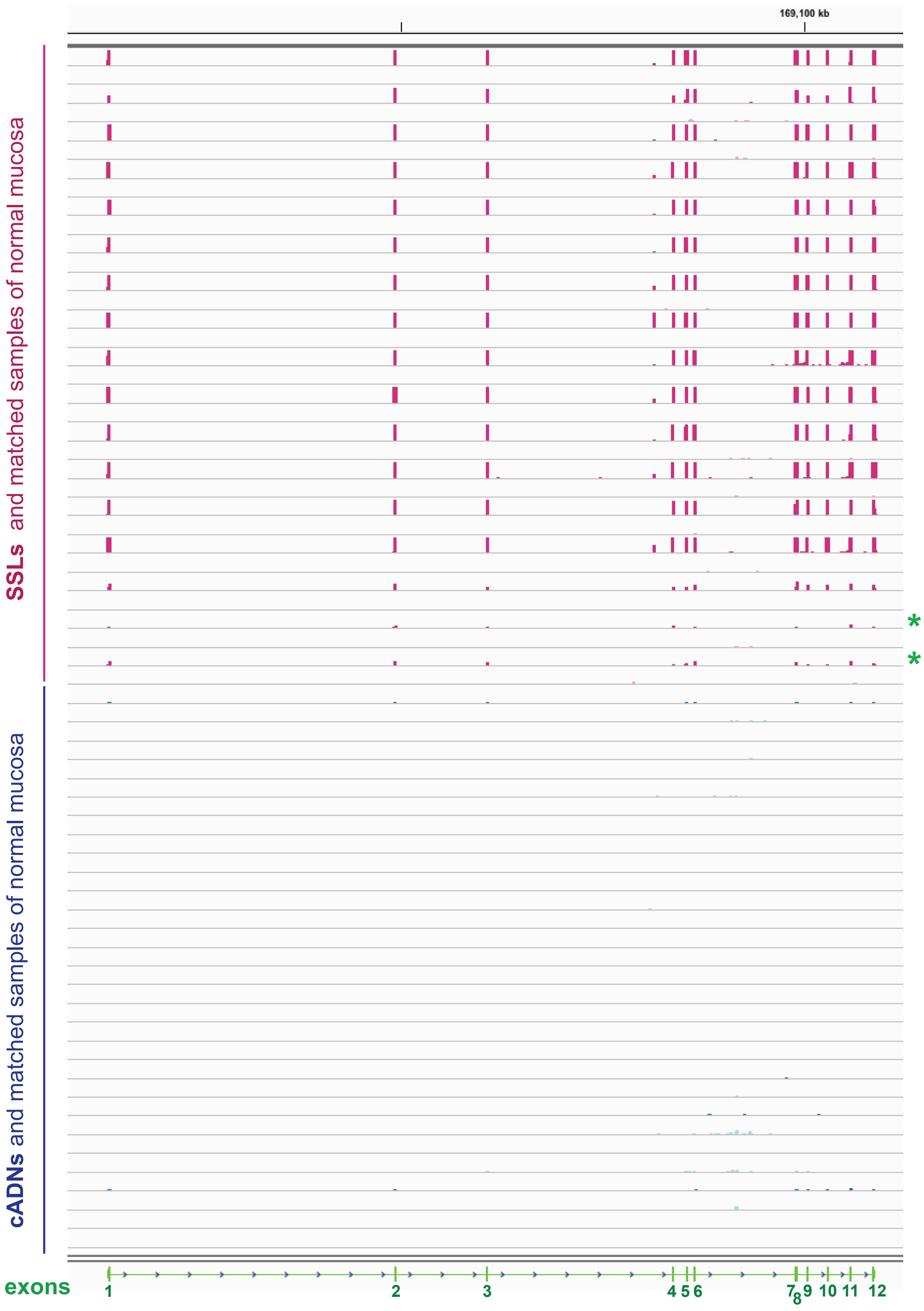

*The 2 lesions showing histologic features of both SSLs and cADNs (samples S6 and S16 of Table 1, Parker H. et al. 2018)

C *ACHE* mRNA expression (chromosome 7)

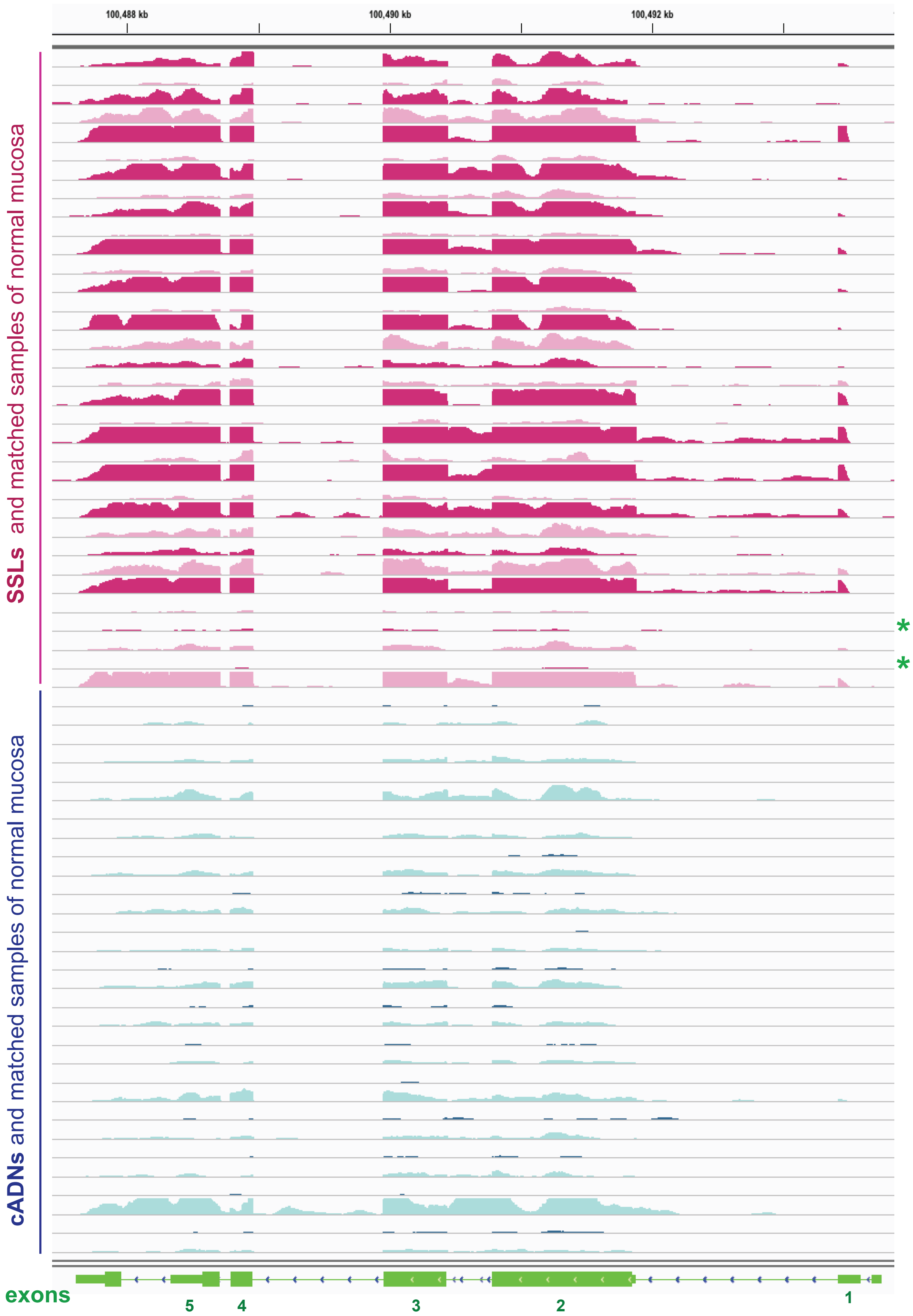

*The 2 lesions showing histologic features of both SSLs and cADNs (samples S6 and S16 of Table 1, Parker H. et al. 2018)

D ***SEMG1*** **mRNA expression (chromosome 20)**

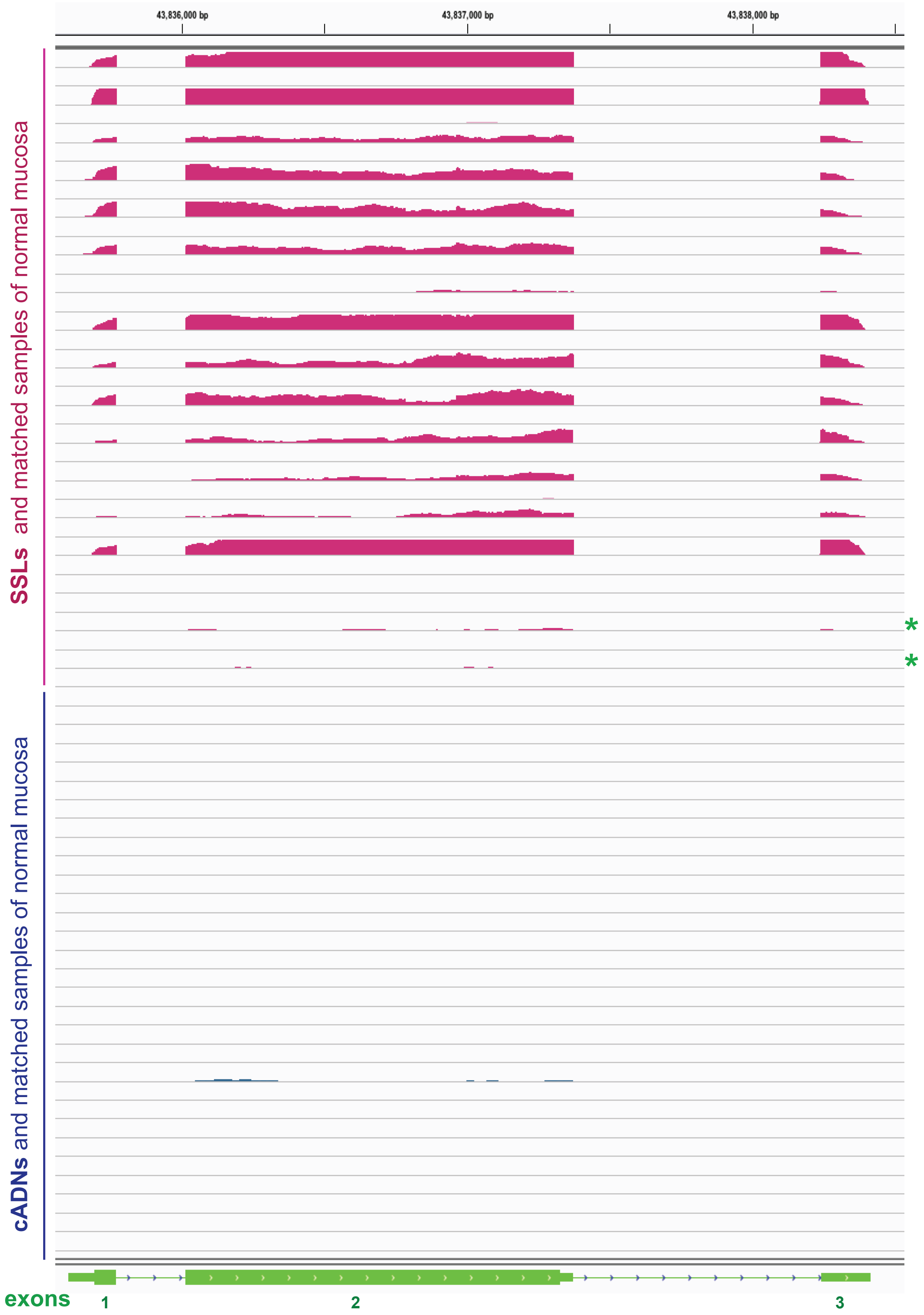

*The 2 lesions showing histologic features of both SSLs and cADNs (samples S6 and S16 of Table 1, Parker H. et al. 2018)

E ***AQP5*** **mRNA expression (chromosome 12)**

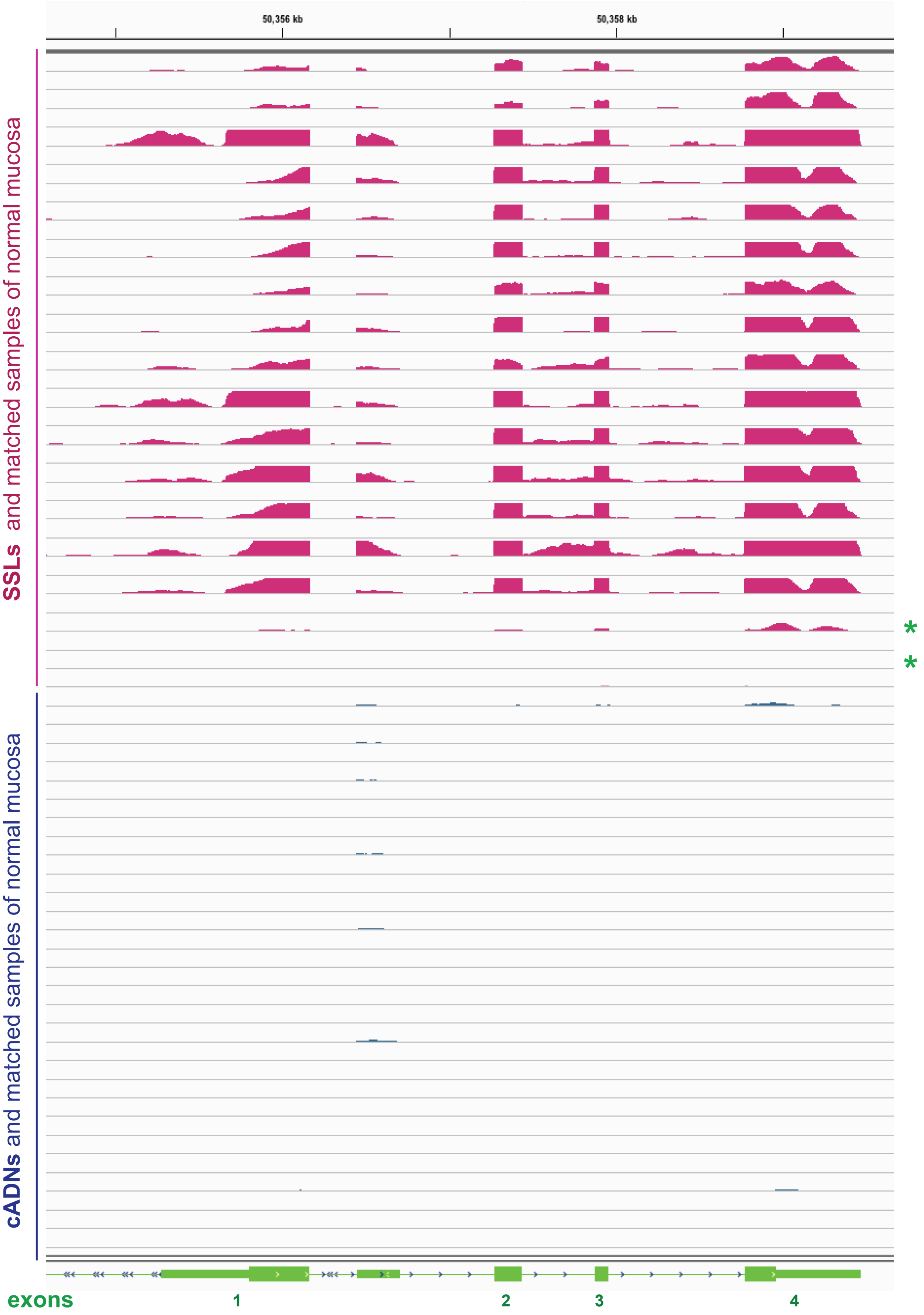

*The 2 lesions showing histologic features of both SSLs and cADNs (samples S6 and S16 of Table 1, Parker H. et al. 2018)

F

LINC00520 mRNA expression (chromosome 14)

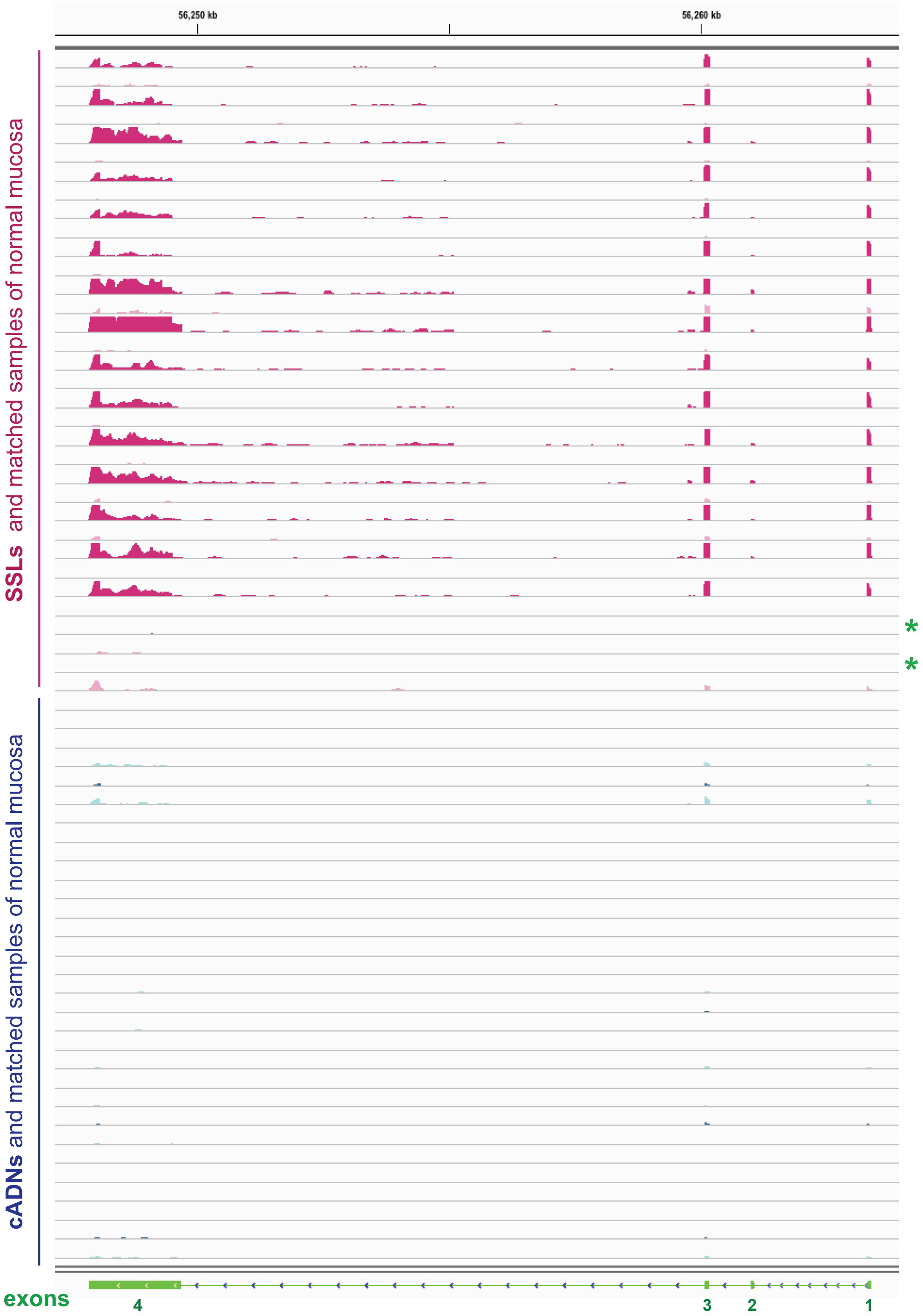

*The 2 lesions showing histologic features of both SSLs and cADNs (samples S6 and S16 of Table 1, Parker H. et al. 2018)

G *ZIC5 and ZIC2* mRNA expression (chromosome 13)

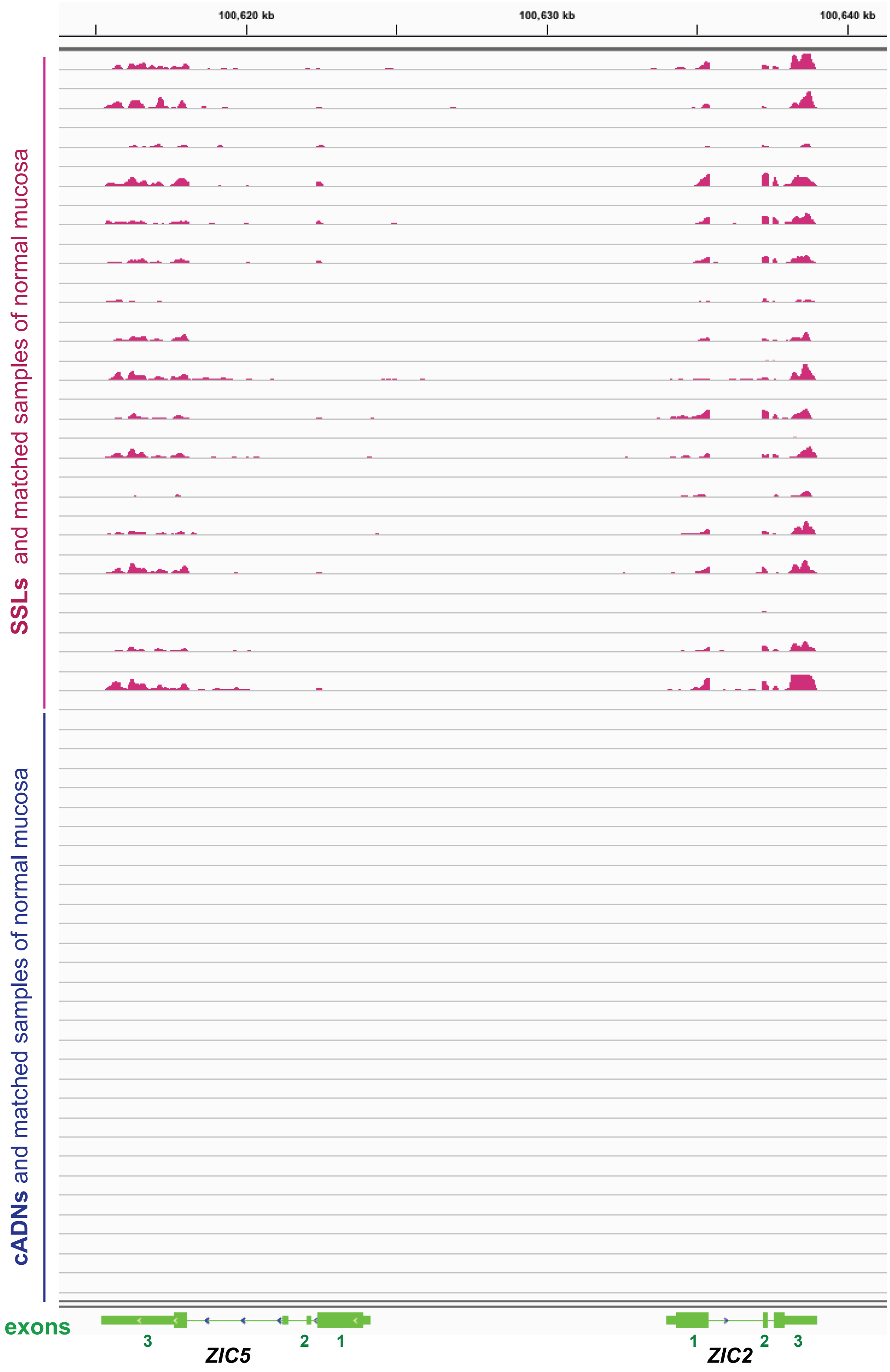

H ***FOXD1*** **mRNA expression (chromosome 5)**

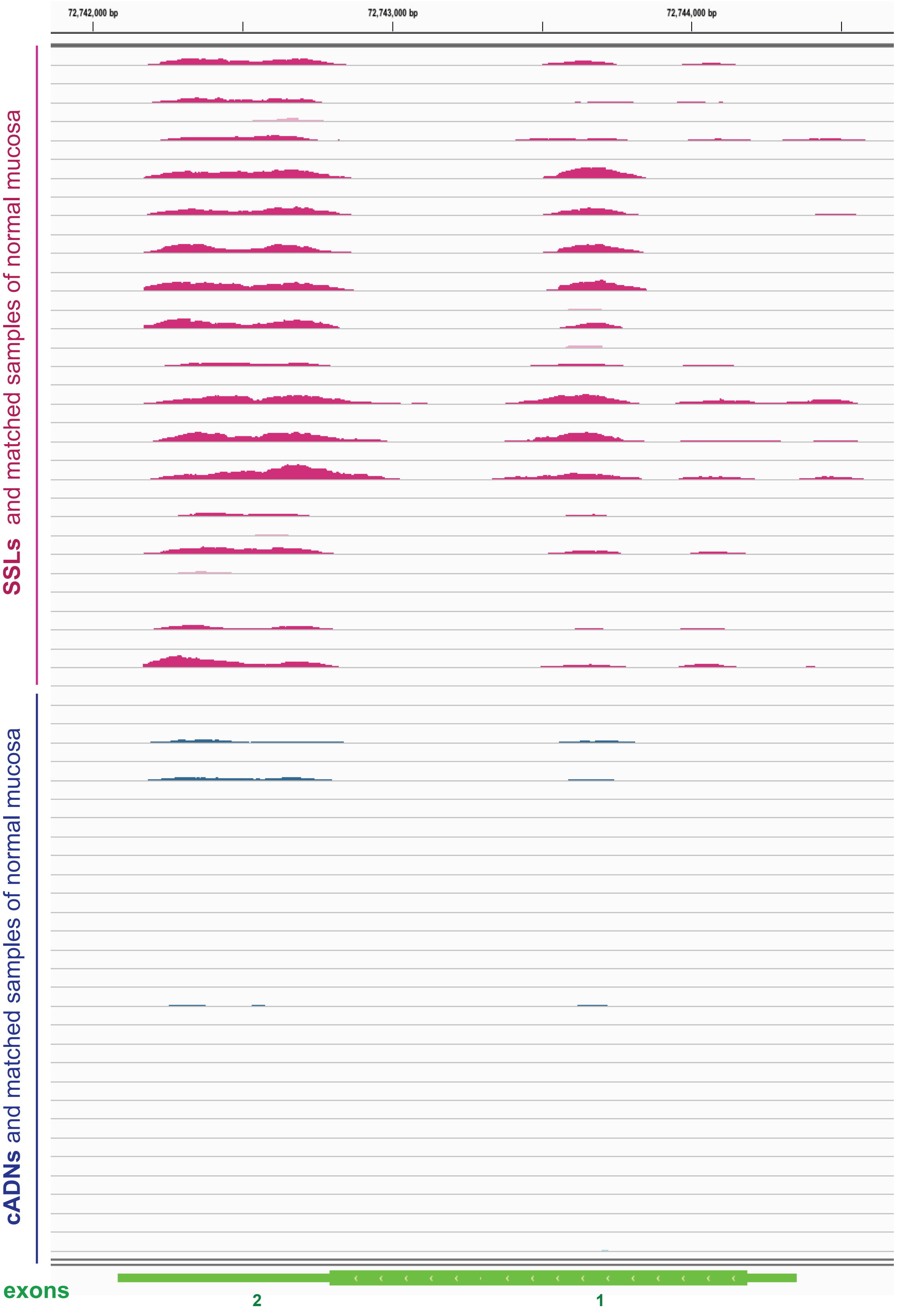

I ***NKD1*** **mRNA expression (chromosome 16)**

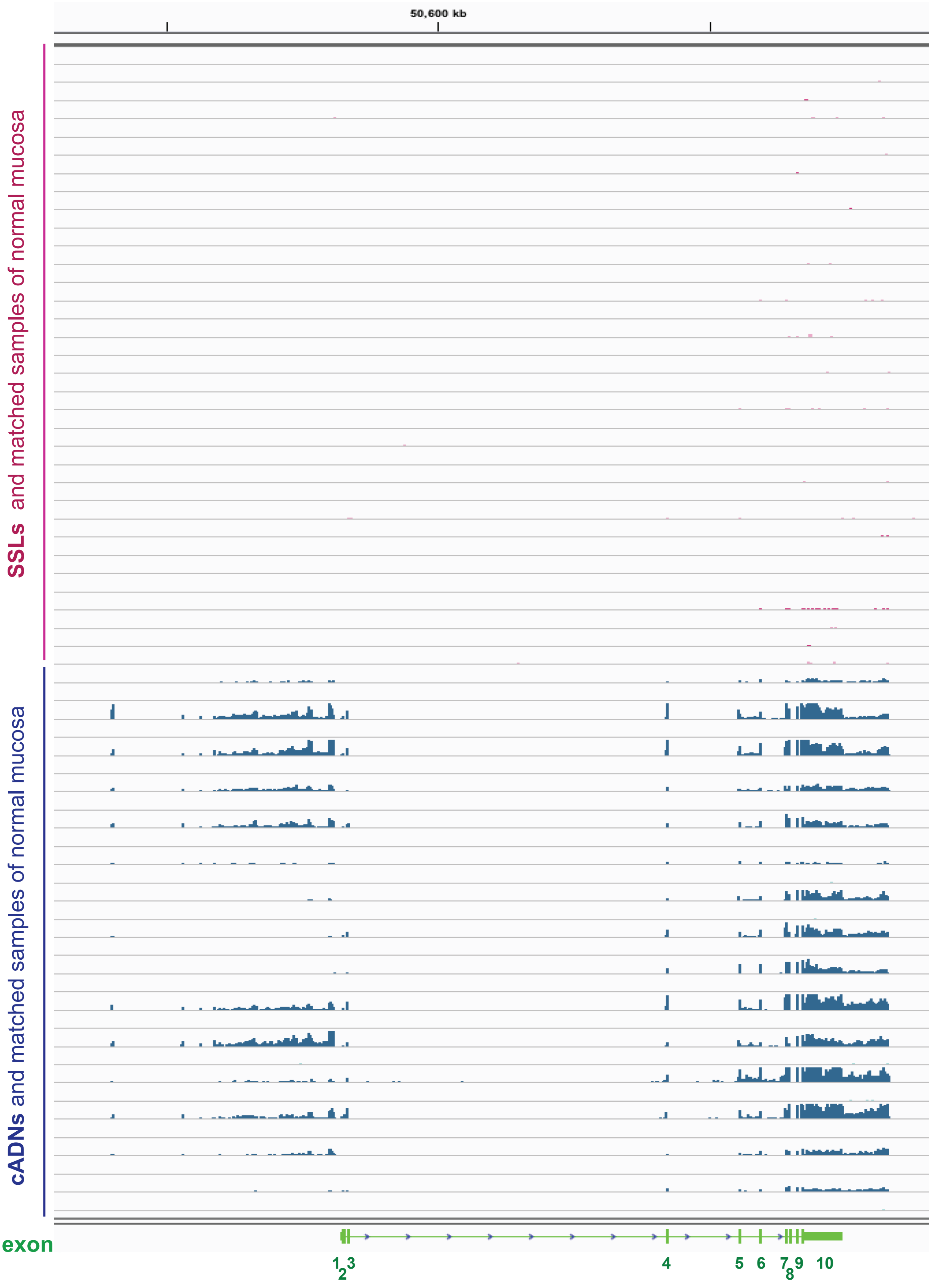

J

APOBEC1 mRNA expression (chromosome 12)

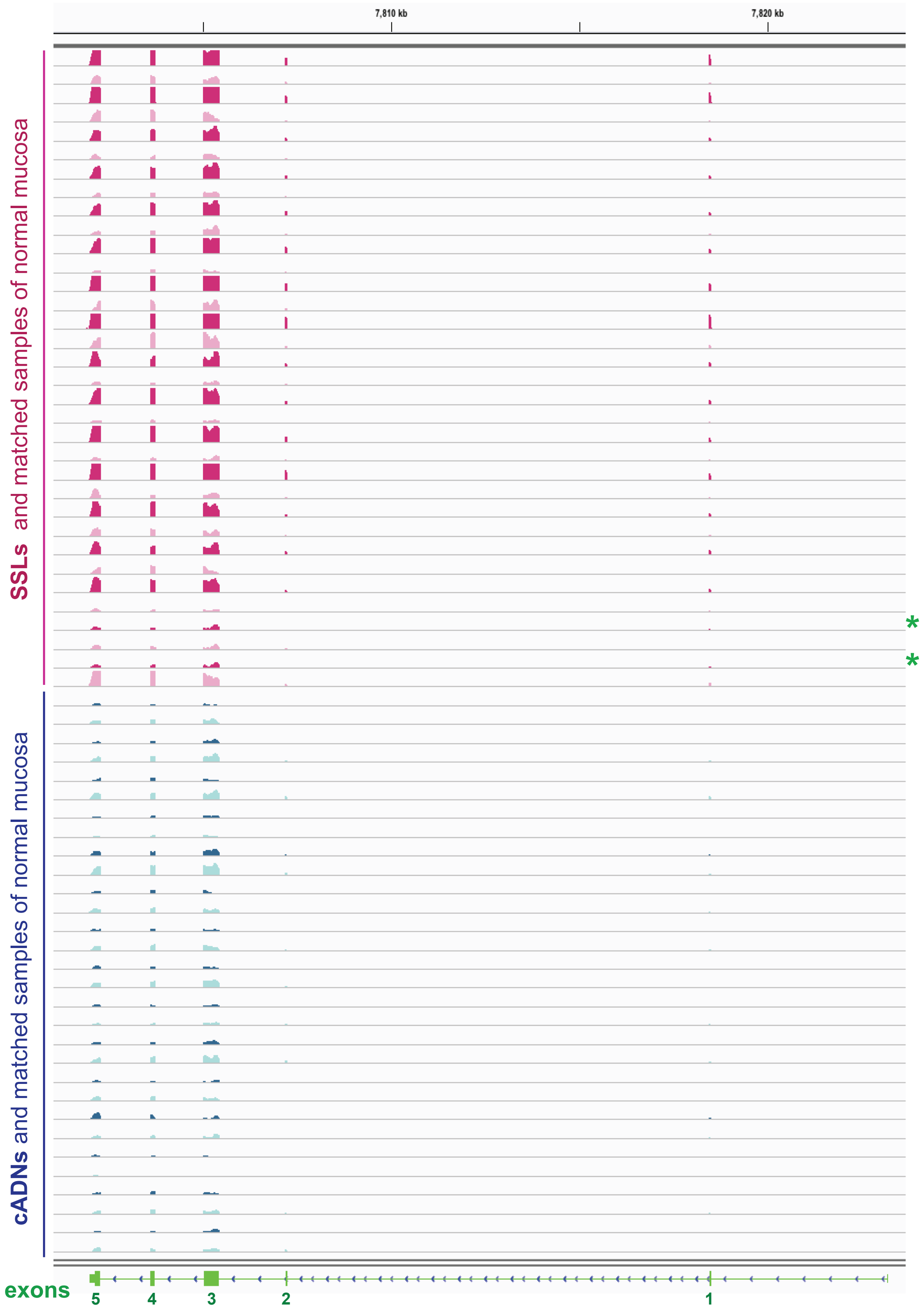

*The 2 lesions showing histologic features of both SSLs and cADNs (samples S6 and S16 of Table 1, Parker H. et al. 2018)

K ***MUC5AC*** **mRNA expression (chromosome 11)**

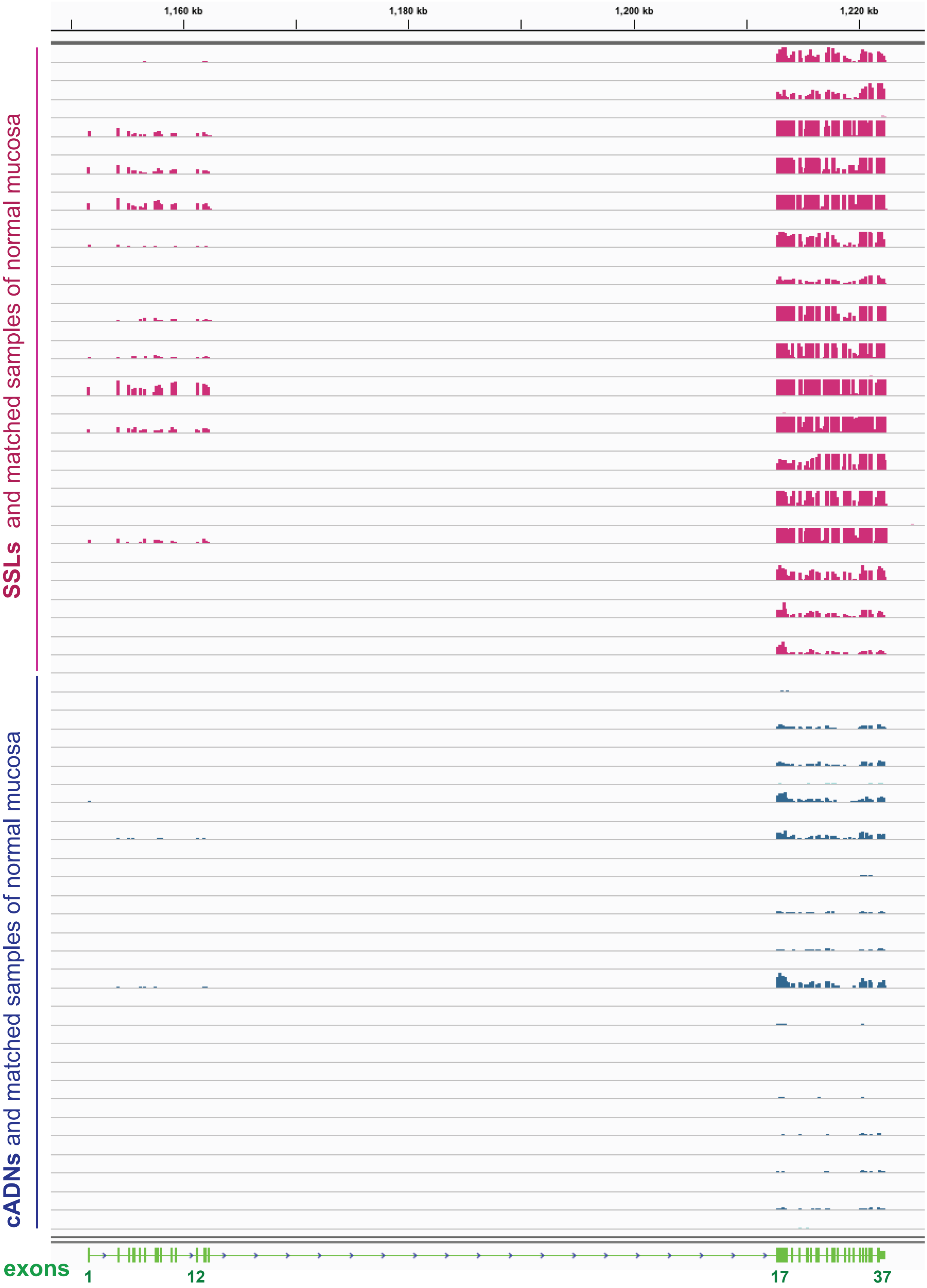

L ***HOXD13*** **mRNA expression (chromosome 2)**

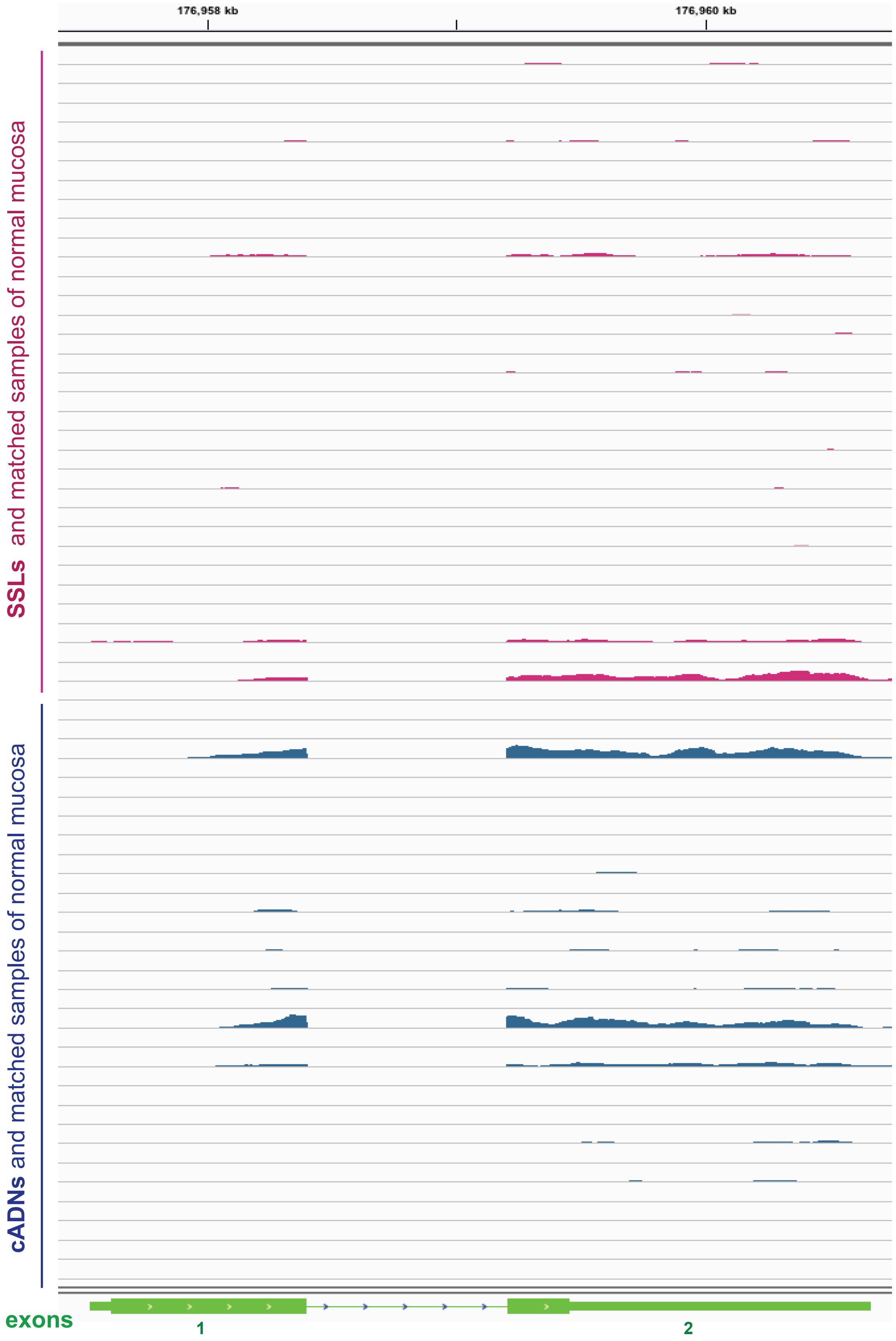

M ***HOXB13*** **mRNA expression (chromosome 17)**

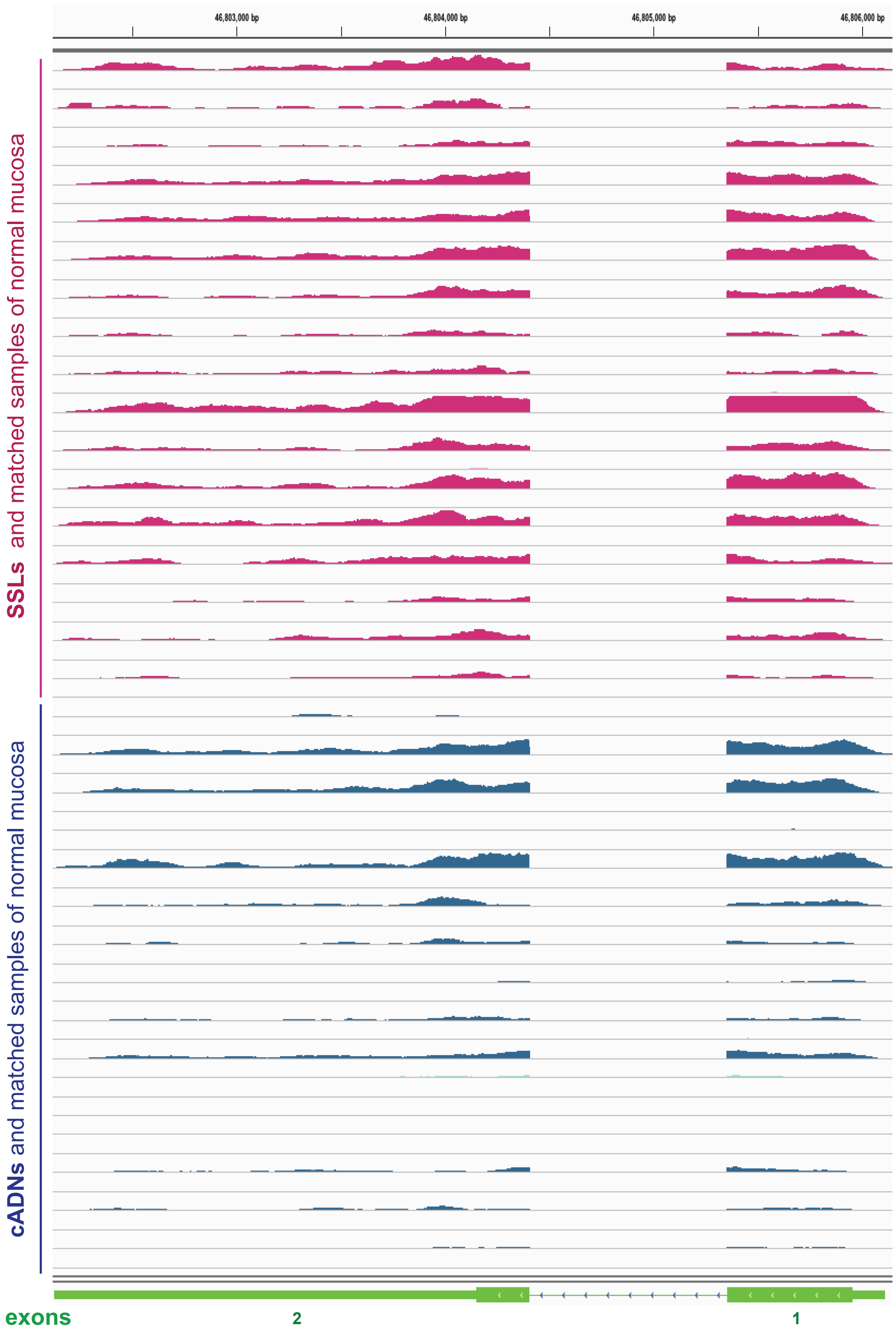

N ***FAM3B*** **mRNA expression (chromosome 21)**

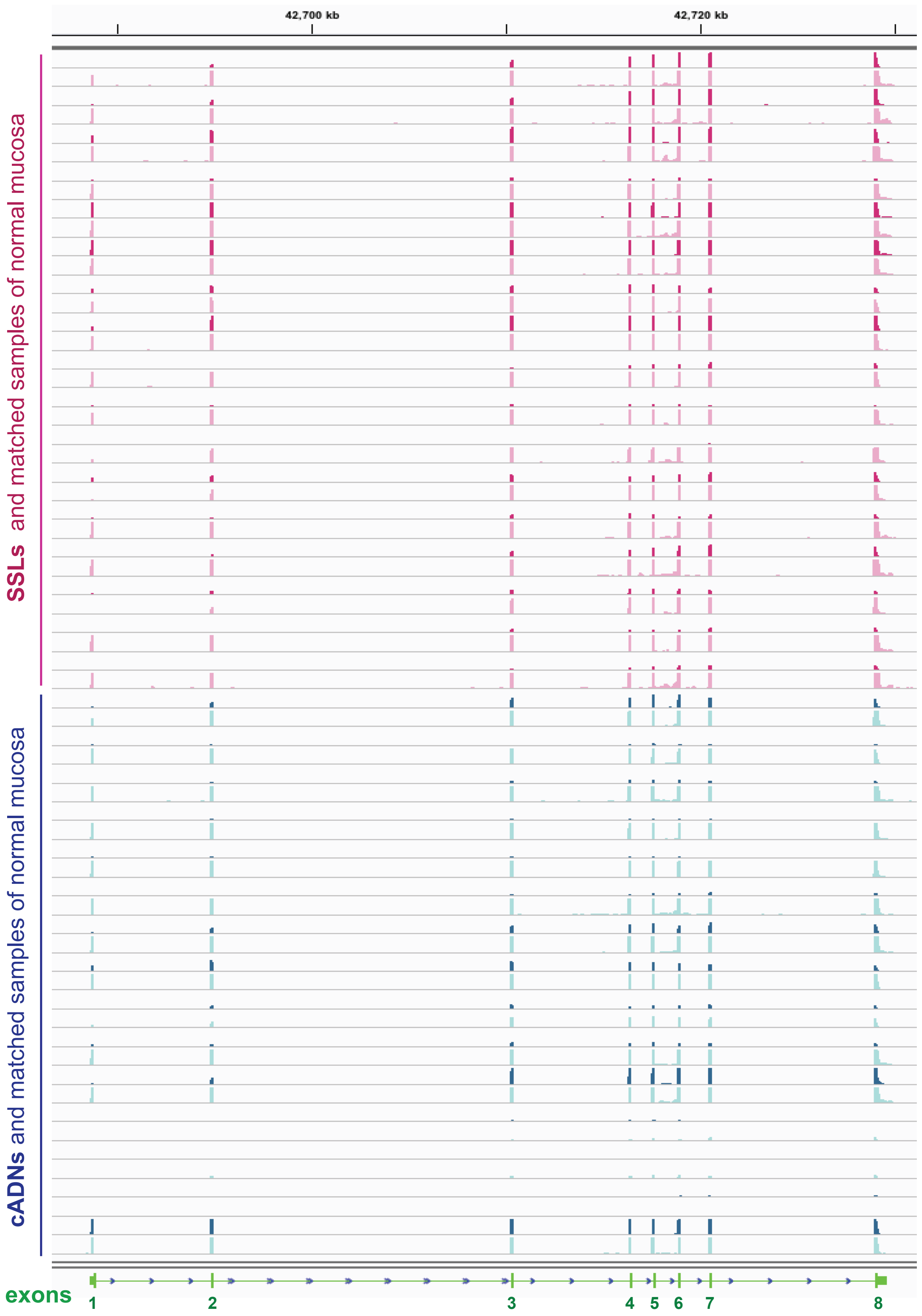

O

AGRN mRNA expression (chromosome 1)

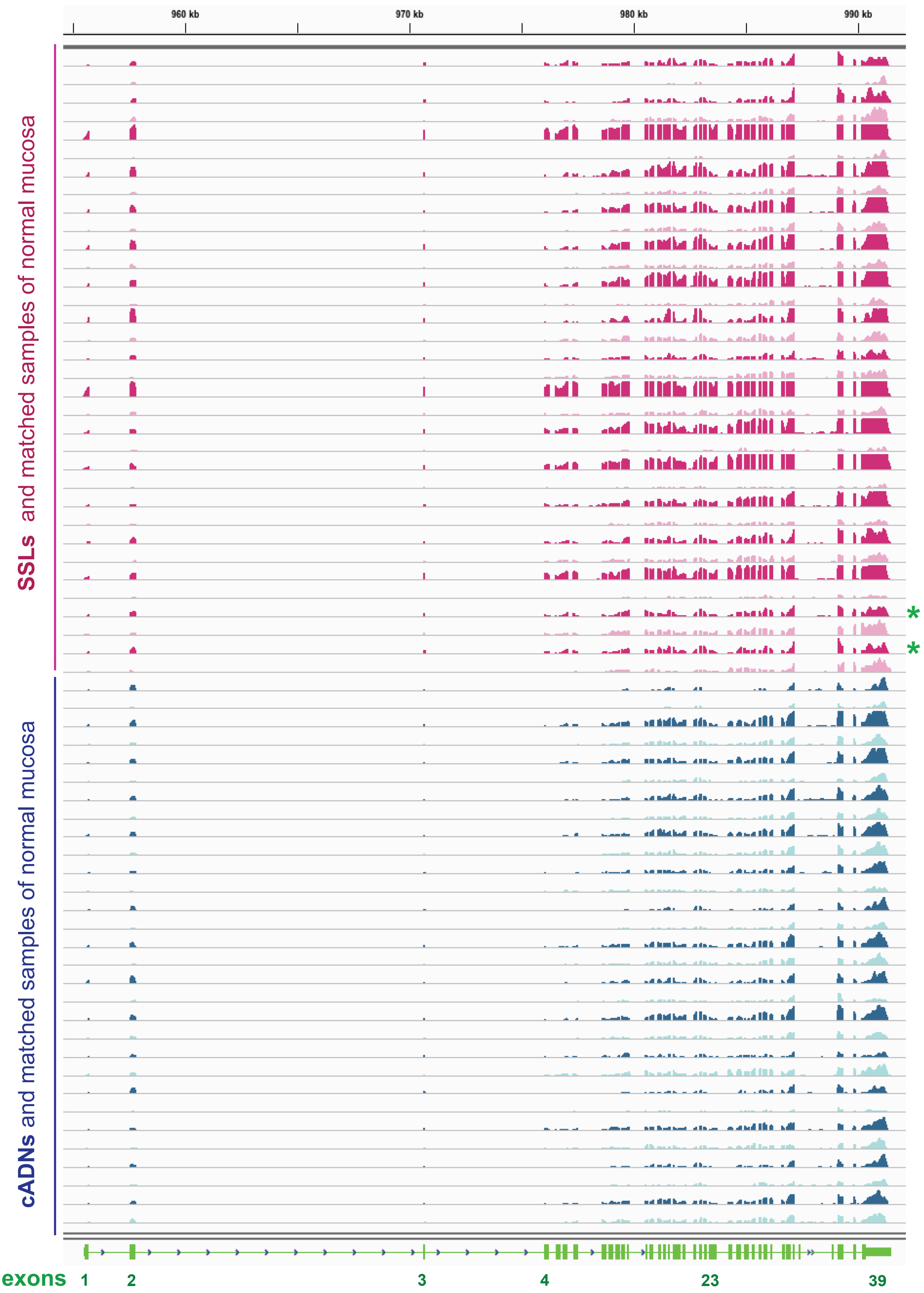

*The 2 lesions showing histologic features of both SSLs and cADNs (samples S6 and S16 of Table 1, Parker H. et al. 2018)
