# Supplementary material for: An “expressionistic” look at serrated precancerous colorectal lesions
Source: Diagn Pathol. 2021 Jan 10;16:4. doi: 10.1186/s13000-020-01064-1 (PMC7797135; doi:10.1186/s13000-020-01064-1)

A

**Samples from Kanth P. et al., 2016** (GSE76987 RNA sequencing data)					
SRR ID	name	tissue	colon segment	age	gender
SRR3106772	SSA/P -1	Serrated Polyposis	ascending	28	M
SRR3106773	SSA/P -2	Serrated Polyposis	ascending	51	M
SRR3106774	SSA/P -3	Serrated Polyposis	transversum	51	M
SRR3106775	SSA/P -4	Serrated Polyposis	cecum	76	M
SRR3106776	SSA/P -5	Serrated Polyposis	ascending	76	M
SRR3106777	SSA/P -6	Serrated Polyposis	transversum	25	M
SRR3106778	SSA/P -7	Serrated Polyposis	ascending	31	M
SRR3106779	SSA/P -8	Serrated Polyposis	ascending	29	F
SRR3106780	SSA/P -9	Serrated Polyposis	descending	20	F
SRR3106781	SSA/P -10	Serrated Polyposis	descending	20	F
SRR3106782	SSA/P -11	Serrated Polyposis	ascending	47	F
SRR3106783	SSA/P -12	Serrated Polyposis	ascending	47	F
SRR3106784	SSA/P -13	Sporadic SSA/P	right flexure	58	M
SRR3106785	SSA/P -14	Sporadic SSA/P	sigmoid	51	F
SRR3106786	SSA/P -15	Sporadic SSA/P	ascending	65	M
SRR3106787	SSA/P -16	Sporadic SSA/P	cecum	67	F
SRR3106788	SSA/P -17	Sporadic SSA/P	cecum	61	M
SRR3106789	SSA/P -18	Sporadic SSA/P	transversum	68	M
SRR3106790	SSA/P -19	Sporadic SSA/P	cecum	67	F
SRR3106791	SSA/P -20	Sporadic SSA/P	ascending	73	M
SRR3106792	SSA/P -21	Sporadic SSA/P	rectum	64	M
SRR3106793	HP -1	HP	sigmoid	49	M
SRR3106794	HP -2	HP	sigmoid	66	M
SRR3106795	HP -3	HP	rectum	64	M
SRR3106796	HP -4	HP	sigmoid	56	M
SRR3106797	HP -5	HP	right flexure	64	M
SRR3106798	HP -6	HP	rectum	59	M
SRR3106799	HP -7	HP	sigmoid	64	M
SRR3106800	HP -8	HP	rectum	64	M
SRR3106801	HP -9	HP	sigmoid	64	M
SRR3106802	HP -10	HP	left flexure	57	F
SRR3106803	AP -1	cADN	ascending	49	M
SRR3106804	AP -2	cADN	descending	65	M
SRR3106805	AP -3	cADN	transversum	80	M
SRR3106806	AP -4	cADN	ascending	66	M
SRR3106807	AP -5	cADN	transversum	66	M
SRR3106808	AP -6	cADN	cecum	44	M
SRR3106809	AP -7	cADN	transversum	44	M
SRR3106810	AP -8	cADN	ascending	53	F
SRR3106811	AP -9	cADN	ascending	64	M
SRR3106812	AP -10	cADN	ascending	50	M
SRR3106813	CA-1	CRC	ascending	NA	NA
SRR3106814	CA-2	CRC	ascending	NA	NA
SRR3106815	CA-3	CRC	ascending	NA	NA
SRR3106816	CA-4	CRC	ascending	NA	NA
SRR3106817	UR-1	Uninvolved right colon	ascending	49	M
SRR3106818	UR-2	Uninvolved right colon	ascending	65	M
SRR3106819	UR-3	Uninvolved right colon	ascending	80	M
SRR3106820	UR-4	Uninvolved right colon	ascending	66	M
SRR3106821	UR-5	Uninvolved right colon	ascending	44	M
SRR3106822	UR-6	Uninvolved right colon	ascending	53	F
SRR3106823	UR-7	Uninvolved right colon	ascending	64	M
SRR3106824	UR-8	Uninvolved right colon	ascending	50	M
SRR3106825	UR-9	Uninvolved right colon	ascending	61	M
SRR3106826	UR-10	Uninvolved right colon	ascending	57	F
SRR3106827	UR-11	Uninvolved right colon	ascending	28	M
SRR3106828	UR-12	Uninvolved right colon	ascending	51	M
SRR3106829	UR-13	Uninvolved right colon	ascending	76	M
SRR3106830	UR-14	Uninvolved right colon	ascending	25	M
SRR3106831	UR-15	Uninvolved right colon	ascending	29	F
SRR3106832	UL-1	Uninvolved left colon	sigmoid	20	F
SRR3106833	UL-2	Uninvolved left colon	sigmoid	51	F
SRR3106834	UL-3	Uninvolved left colon	sigmoid	64	M
SRR3106835	UL-4	Uninvolved left colon	sigmoid	56	M
SRR3106836	UL-5	Uninvolved left colon	sigmoid	64	M
SRR3106837	UL-6	Uninvolved left colon	sigmoid	57	F
SRR3106838	CR-1	Control right colon	ascending	46	F
SRR3106839	CR-2	Control right colon	ascending	50	F
SRR3106840	CR-3	Control right colon	ascending	50	M
SRR3106841	CR-4	Control right colon	ascending	55	F
SRR3106842	CR-5	Control right colon	ascending	68	M
SRR3106843	CR-6	Control right colon	ascending	51	M
SRR3106844	CR-7	Control right colon	ascending	50	F
SRR3106845	CR-8	Control right colon	ascending	52	F
SRR3106846	CR-9	Control right colon	ascending	61	F
SRR3106847	CR-10	Control right colon	ascending	52	M
SRR3106848	CL-1	Control left colon	sigmoid	46	F
SRR3106849	CL-2	Control left colon	sigmoid	50	F
SRR3106850	CL-3	Control left colon	sigmoid	50	M
SRR3106851	CL-4	Control left colon	sigmoid	55	F
SRR3106852	CL-5	Control left colon	sigmoid	68	M
SRR3106853	CL-6	Control left colon	sigmoid	51	M
SRR3106854	CL-7	Control left colon	sigmoid	50	F
SRR3106855	CL-8	Control left colon	sigmoid	52	F
SRR3106856	CL-9	Control left colon	sigmoid	61	F
SRR3106857	CL-10	Control left colon	sigmoid	52	M

B

HOXD13 **mRNA expression (samples from Kanth P. et al., 2016, ref.45)**

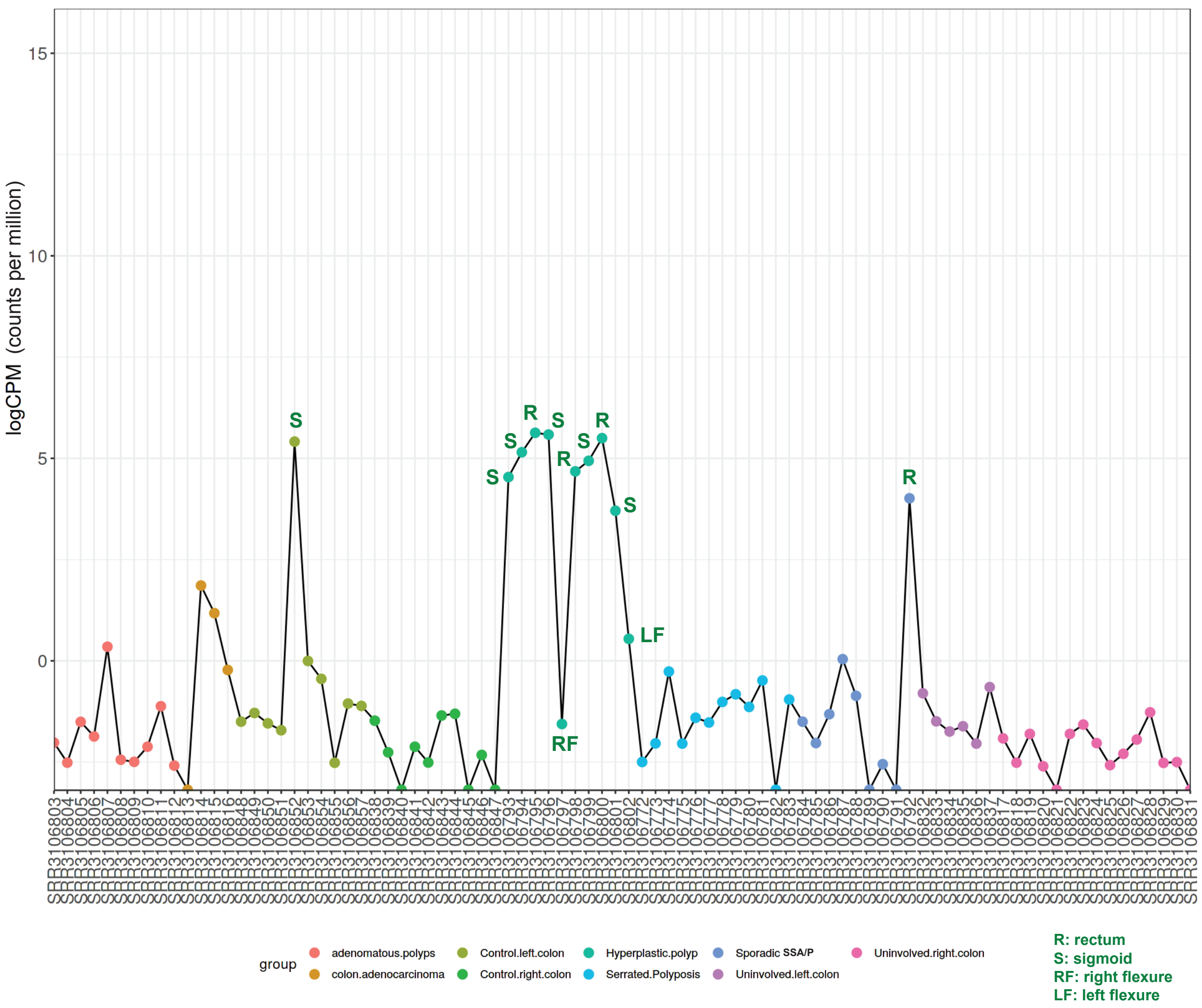

C

HOXB13 **mRNA expression (samples from Kanth P. et al., 2016, ref.45)**

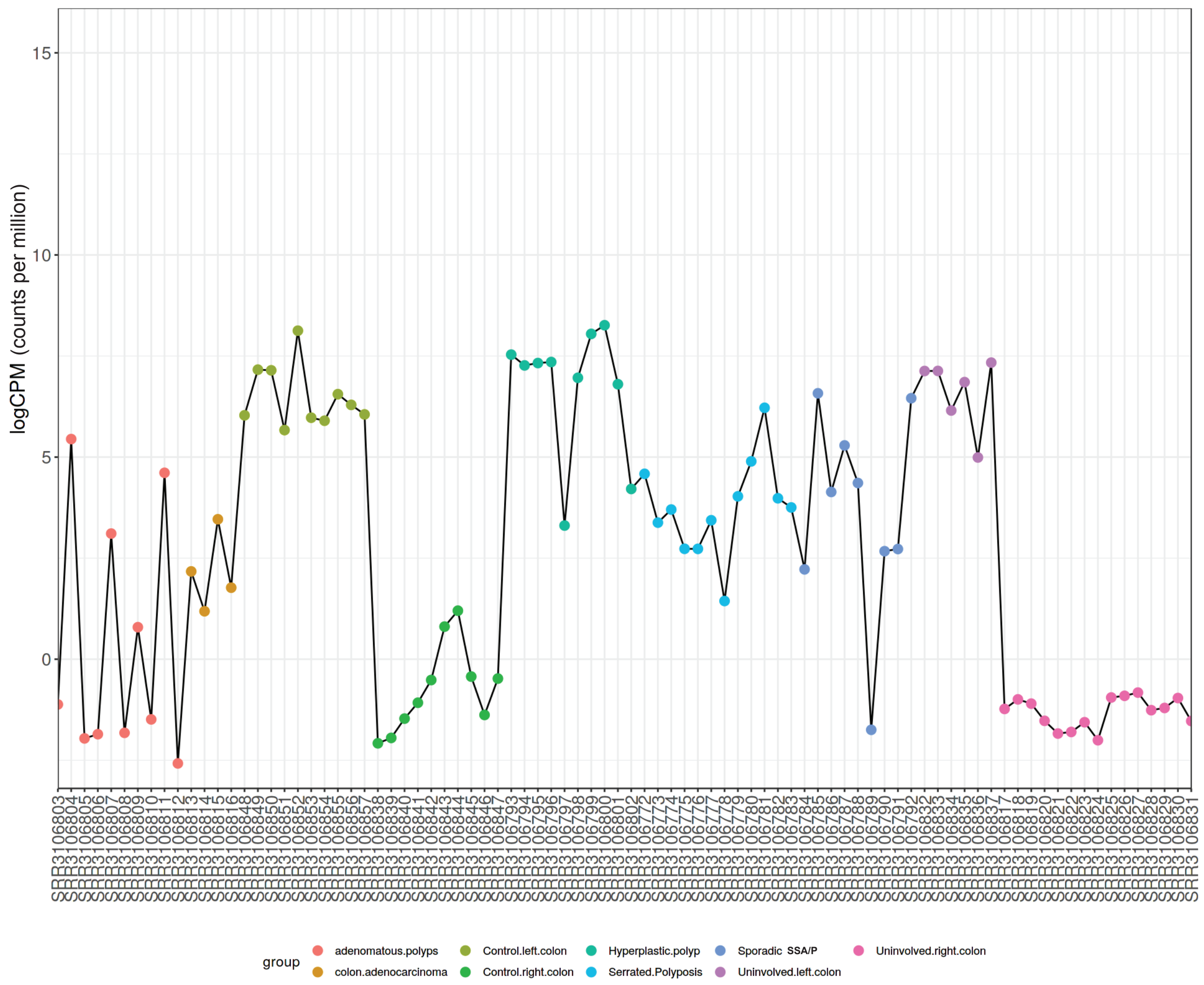

D

FAM3B **mRNA expression (samples from Kanth P. et al., 2016, ref.45)**

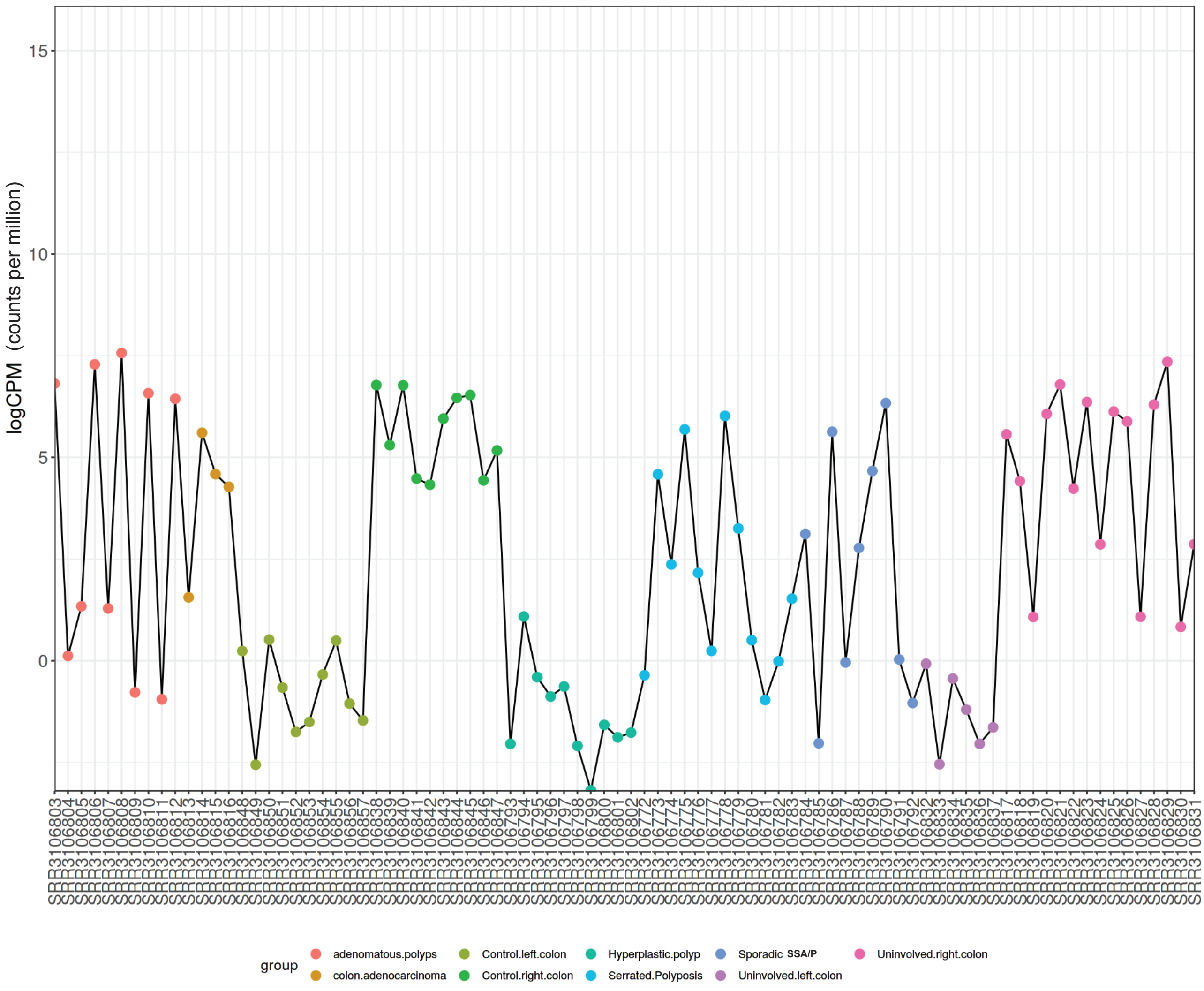

E

EVX2 mRNA expression (samples from Kanth P. et al., 2016, ref.45)

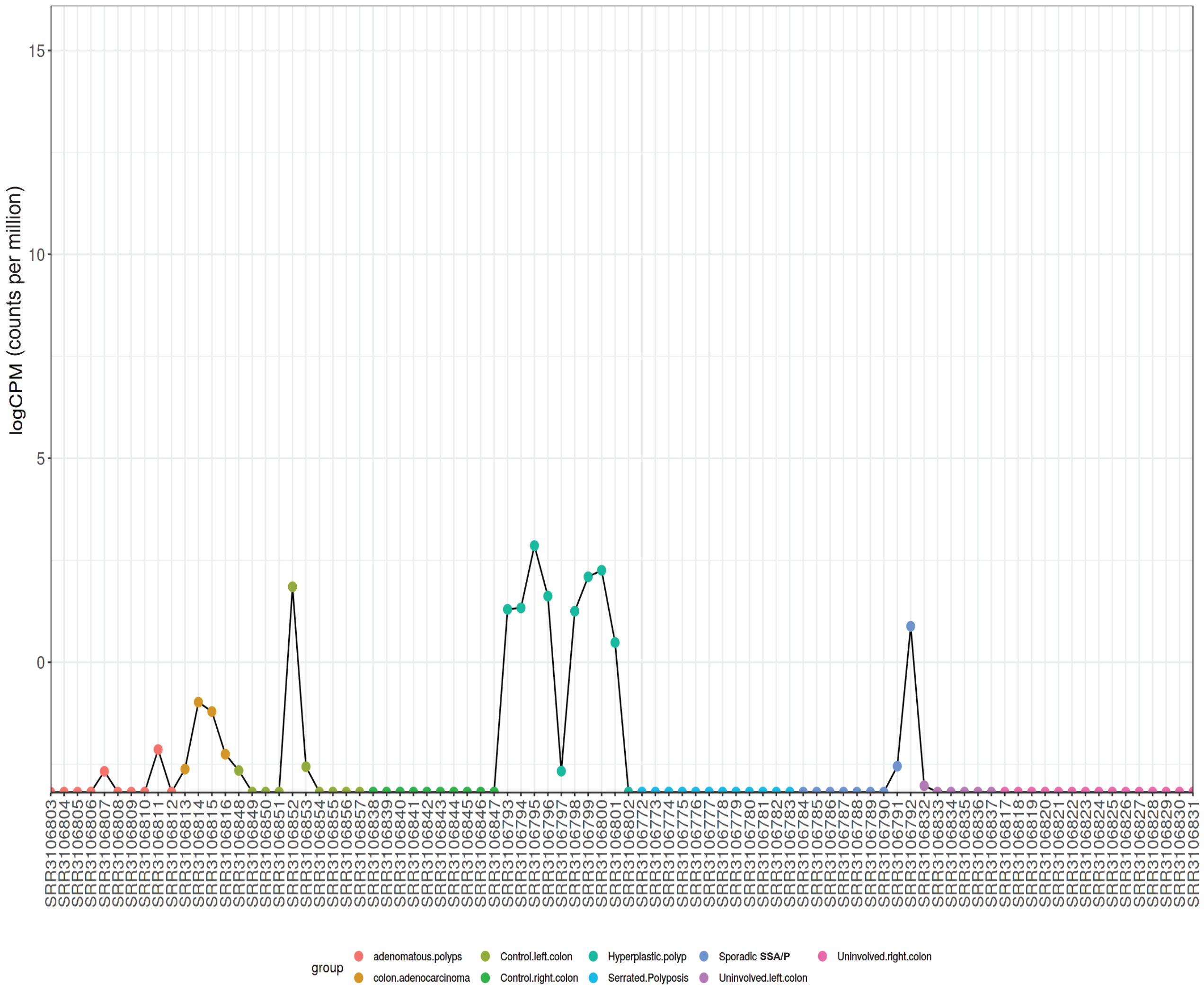

F

PRAC1 **mRNA expression (samples from Kanth P. et al., 2016, ref.45)**

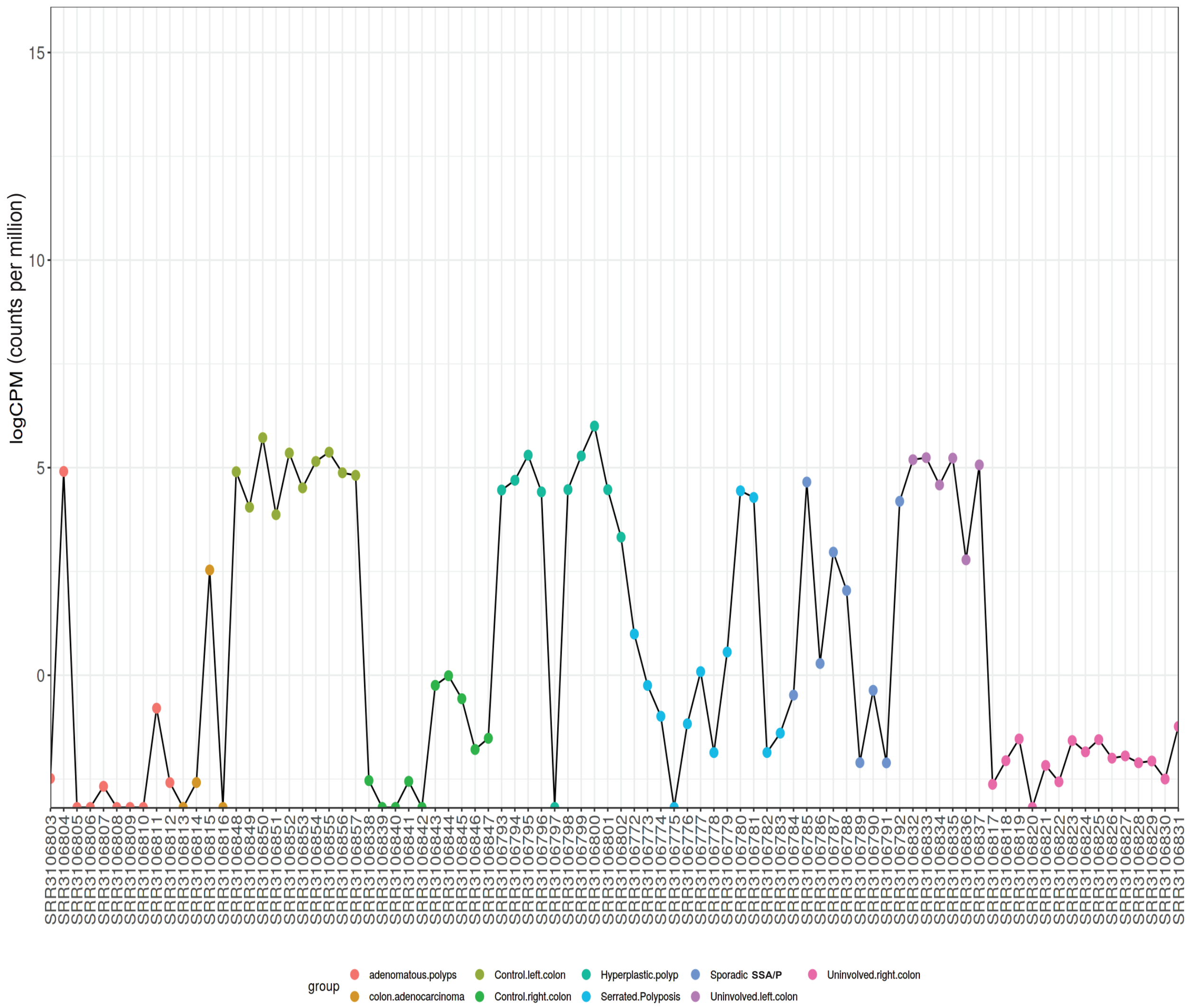

G

INSL5 mRNA expression (samples from Kanth P. et al., 2016, ref.45)

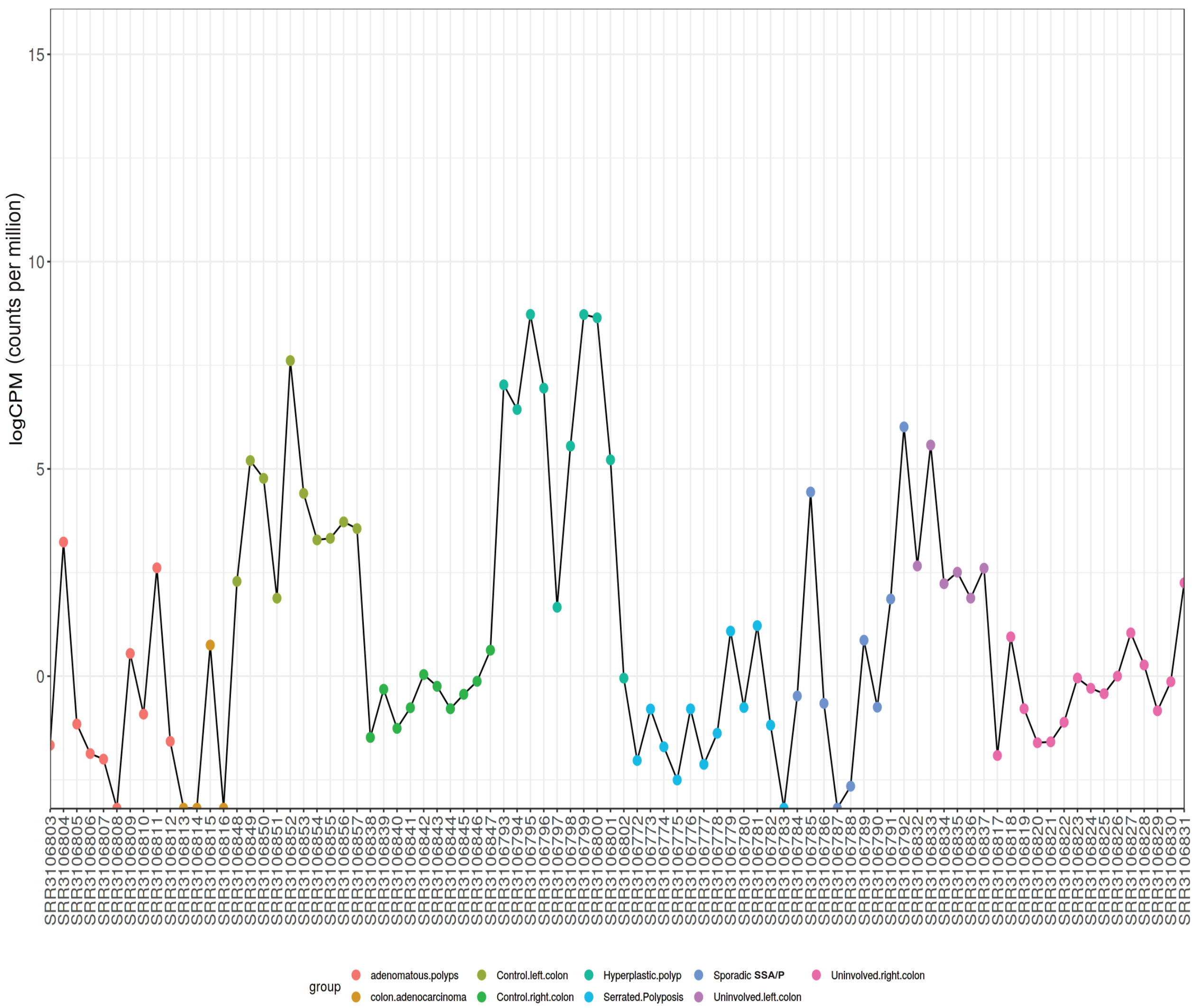

H

OR51E2 **mRNA expression (samples from Kanth P. et al., 2016, ref.45)**

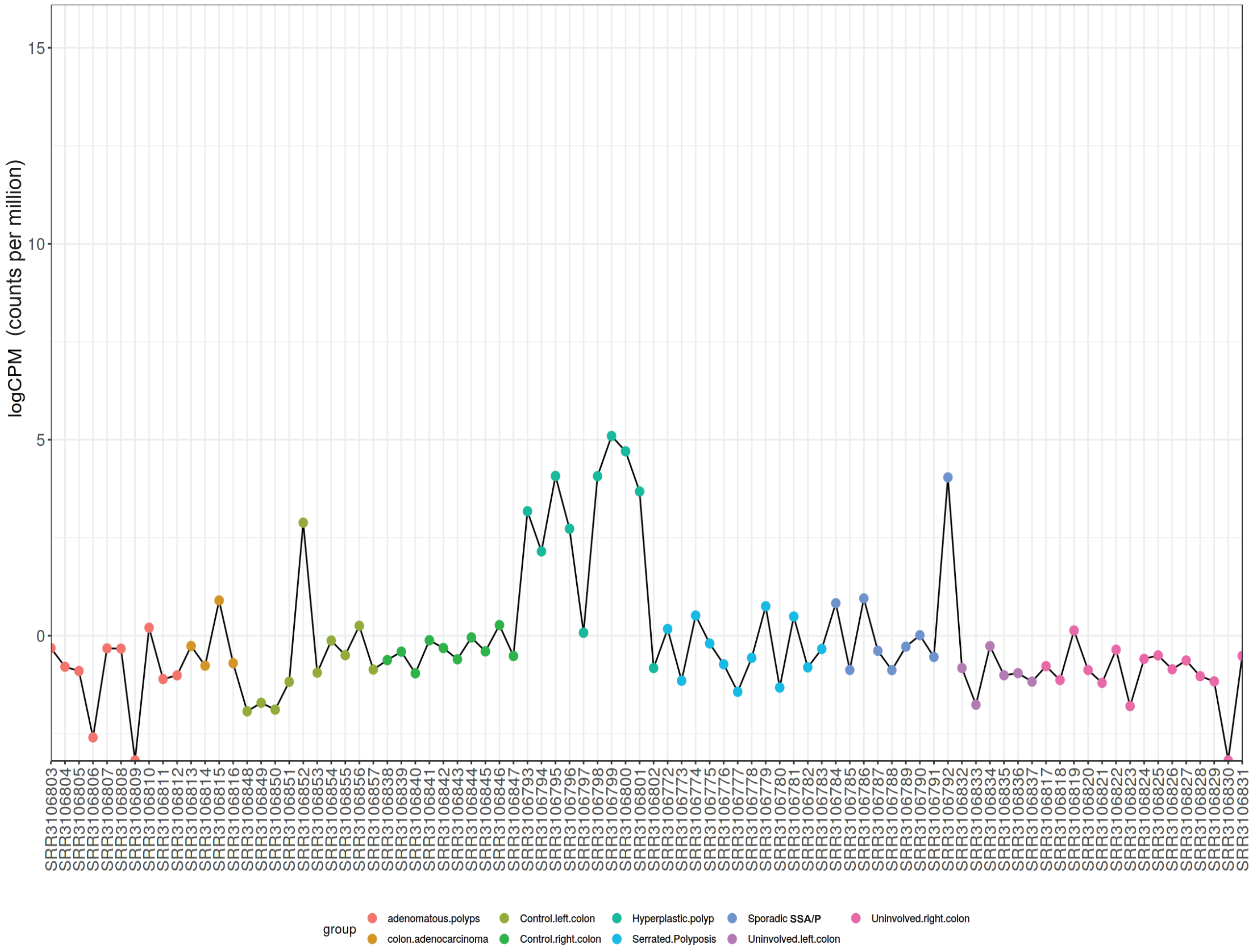

I

CPB1 mRNA expression (samples from Kanth P. et al., 2016, ref.45)

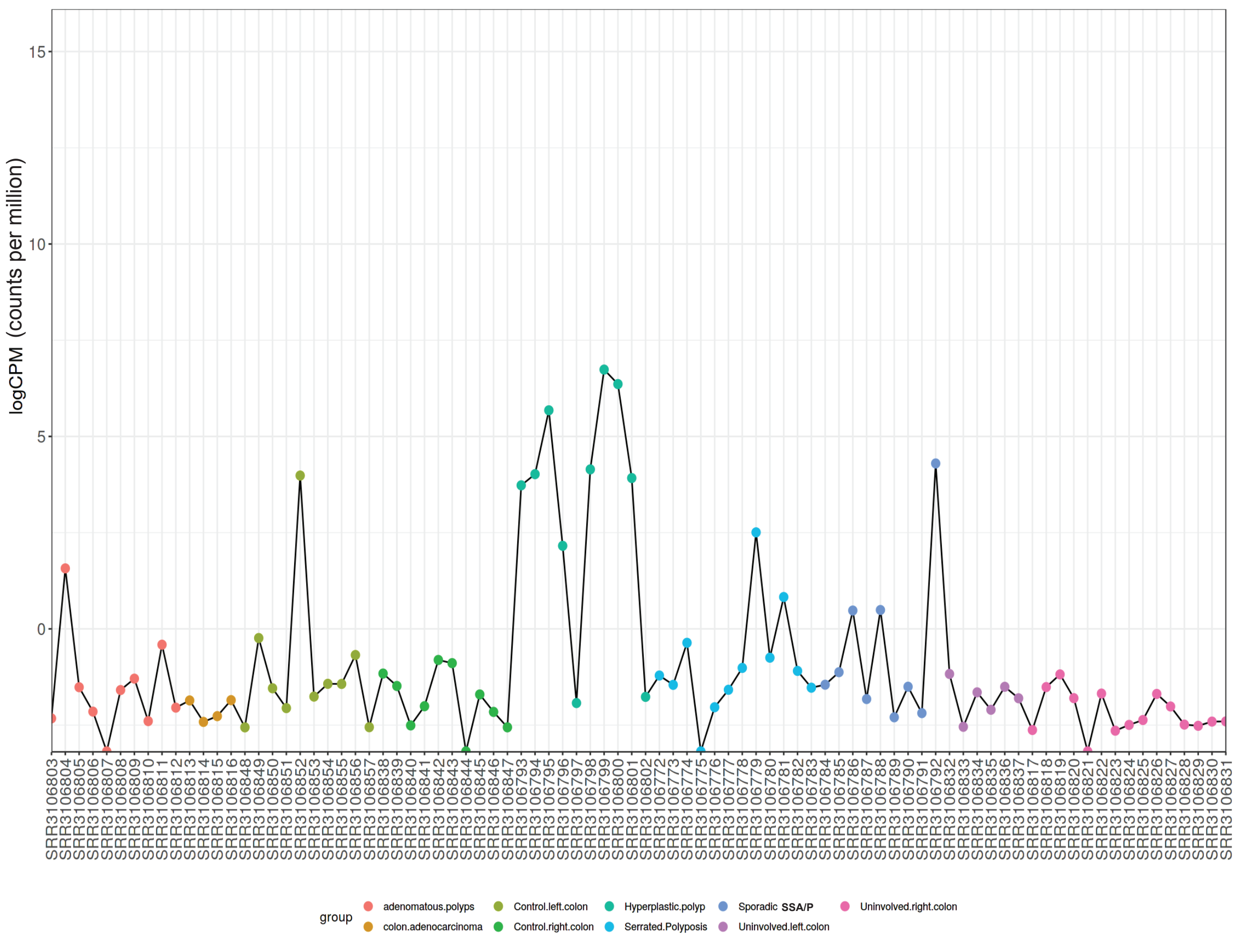

J

ST6GAL2 **mRNA expression (samples from Kanth P. et al., 2016, ref.45)**

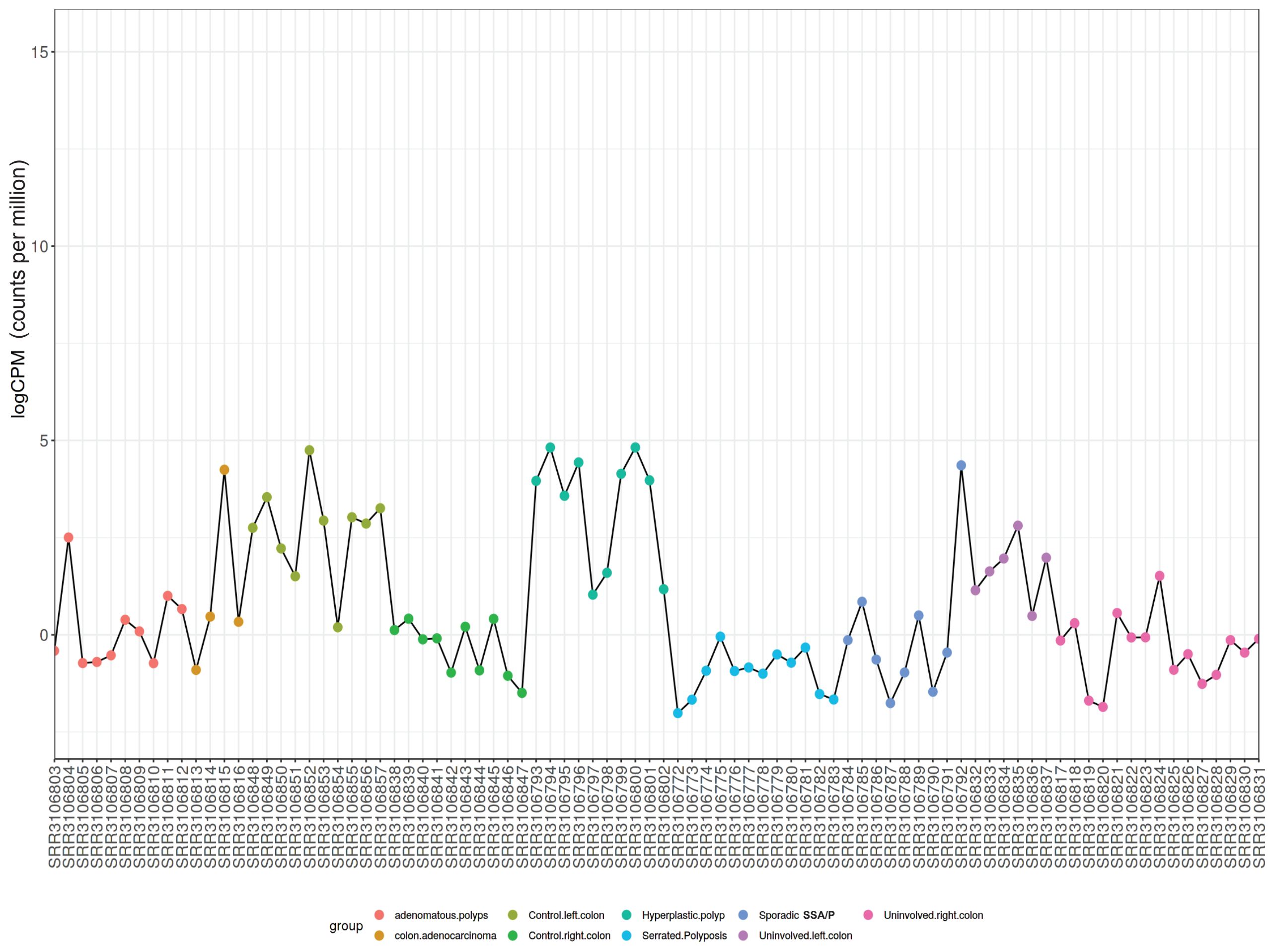

K

AGRN **mRNA expression (samples from Kanth P. et al., 2016, ref.45)**

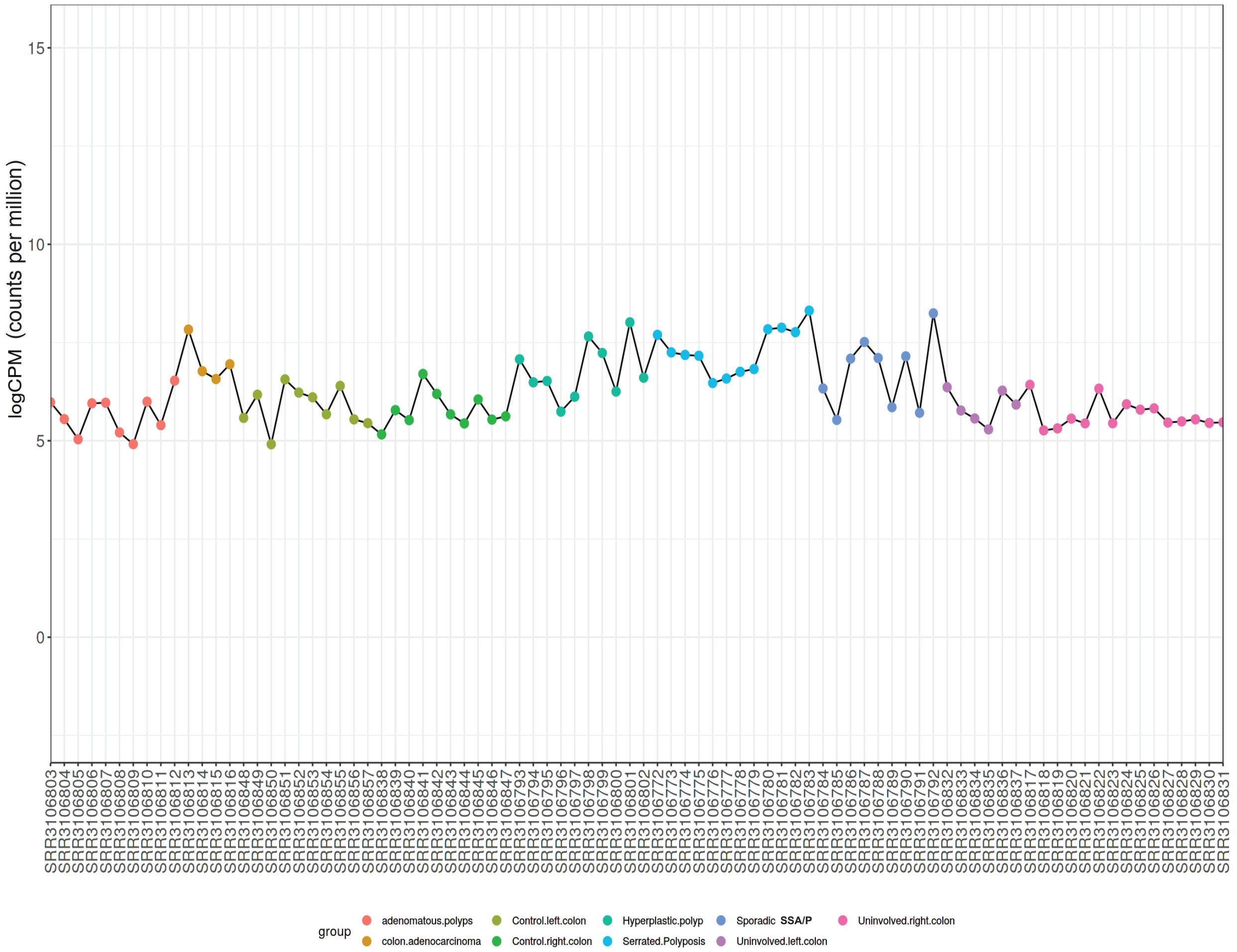

Supplement: Supplementary file 3 — Additional file 3: Supplementary Figure 3. (Panels A through K). RNA-sequencing-based expression profiles of the targets included in this study based on data published by Kanth et al. (reference [38]). Clinical data on the lesions and normal mucosa samples investigated by Kanth et al. are in panel A, while the RNA expression level (i.e., logCPM: log2 counts per million) of the targets in each tissue is graphically shown in the following panels. [file 13000_2020_1064_MOESM3_ESM.pdf]
