# Supplementary material for: An “expressionistic” look at serrated precancerous colorectal lesions
Source: Diagn Pathol. 2021 Jan 10;16:4. doi: 10.1186/s13000-020-01064-1 (PMC7797135; doi:10.1186/s13000-020-01064-1)
Supplement: Supplementary file 19 — Additional file 19: Supplementary Table 1. mRNA targets chosen for ISH based on RNA sequencing data from Parker H. et al (ref. [21]) and Kanth P. et al (ref. [38]). [file 13000_2020_1064_MOESM19_ESM.pdf]

Supplementary Table 1. mRNA targets chosen for ISH based on RNA sequencing data from Parker H. et al (ref. 21) and Kanth P. et al (ref. 38).

gene name	*gene expression pattern (RNA sequencing)*
VSIG1	SSL-specific expression
ANXA10	SSL-specific expression
ZIC2 / ZIC5	SSL-specific expression (neighbouring genes)
SEMG1	SSL-specific expression
FOXD1	SSL-specific expression
KLK8	SSL-specific expression
AQP5	SSL-specific expression
MUC5AC	SSL-specific expression
LINC00520	SSL-specific expression
NKD1	cADNs-specific
APOBEC1	downregulated in cADNs
ACHE	downregulated in cADNs
HOXD13	HP-specific
EVX2	*HOXD13* neighbouring gene
HOXB13	HP-specific
PRAC1	*HOXB13* neighbouring gene
INSL5	HP-specific
OR51E2	HP-specific
CPB1	HP-specific
ST6GAL2	HP-specific
FAM3B	no expression in HPs
PPIB	positive control: expressed in most of the cells
DapB	negative control (bacterial gene)
XIST	positive (female cells)/negative (male cells) control
